# Macroevolutionary Dynamics in Micro-organisms: Generalists Give Rise to Specialists Across Biomes in the Ubiquitous Bacterial Phylum Myxococcota

**DOI:** 10.1093/molbev/msae088

**Published:** 2024-05-08

**Authors:** Daniel Padfield, Suzanne Kay, Rutger Vos, Christopher Quince, Michiel Vos

**Affiliations:** Environment and Sustainability Institute, Penryn Campus, Penryn TR10 9FE, UK; Environment and Sustainability Institute, Penryn Campus, Penryn TR10 9FE, UK; Naturalis Biodiversity Center, P.O. Box 9517, 2300 RA Leiden, The Netherlands; Institute of Biology Leiden, Leiden University, 2333 BE Leiden, The Netherlands; Organisms and Ecosystems, Earlham Institute, Norwich NR4 7UZ, UK; Gut Microbes and Health, Quadram Institute, Norwich NR4 7UQ, UK; Environment and Sustainability Institute, Penryn Campus, Penryn TR10 9FE, UK; European Centre for Environment and Human Health, Penryn Campus, Penryn TR10 9FE, UK

**Keywords:** macroevolution, microbes, prokaryotes, habitat transitions, specialization, diversification, myxobacteria, comparative phylogenetics

## Abstract

Prokaryotes dominate the Tree of Life, but our understanding of the macroevolutionary processes generating this diversity is still limited. Habitat transitions are thought to be a key driver of prokaryote diversity. However, relatively little is known about how prokaryotes successfully transition and persist across environments, and how these processes might vary between biomes and lineages. Here, we investigate biome transitions and specialization in natural populations of a focal bacterial phylum, the Myxococcota, sampled across a range of replicated soils and freshwater and marine sediments in Cornwall (UK). By targeted deep sequencing of the protein-coding gene *rpoB*, we found >2,000 unique Myxococcota lineages, with the majority (77%) classified as biome specialists and with only <5% of lineages distributed across the salt barrier. Discrete character evolution models revealed that specialists in one biome rarely transitioned into specialists in another biome. Instead, evolved generalism mediated transitions between biome specialists. State-dependent diversification models found variation in speciation rates across the tree, but this variation was independent of biome association or specialization. Our findings were robust to phylogenetic uncertainty, different levels of species delineation, and different assumed amounts of unsampled diversity resulting in an incomplete phylogeny. Overall, our results are consistent with a “jack-of-all-trades” tradeoff where generalists suffer a cost in any individual environment, resulting in rapid evolution of niche specialists and shed light on how bacteria could transition between biomes.

## Introduction

Understanding the ecological and evolutionary forces that structure prokaryote diversity across environments is a central objective in microbial ecology (Quince et al. 2008; Fierer and Lennon 2011; Jaffe et al. 2023). The extent to which different taxa are associated with different biomes, the rate at which taxa transition between these biomes, and how this influences their diversification rates are not yet fully understood. One of the most drastic environmental transitions for both macro- and micro-organisms is that between marine and terrestrial (land and freshwater) biomes, the so-called “salt barrier” (Logares et al. 2009). Salinity is a major determinant in structuring microbial diversity, with distinct shifts in community composition observed over salinity gradients (Dupont et al. 2014). Transitions between marine and terrestrial biomes require substantial re-organization of the proteome (Cabello-Yeves and Rodriguez-Valera 2019; Jurdzinski et al. 2023) and often involve gains and losses of genes and metabolic pathways (Wisniewski-Dyé et al. 2011; Dupont et al. 2014; Eiler et al. 2016; Moghaddam et al. 2016; Simon et al. 2017; Jurdzinski et al. 2023; Sereika et al. 2023). Due to these adaptive challenges, microbe transitions across the salt barrier are thought to be rare (Logares et al. 2009; Jurdzinski et al. 2023).

Tradeoffs between ecological specialization strategies may explain the scarcity of successful transitions across the marine–terrestrial divide in prokaryotes. Generalist taxa that can live in both terrestrial and marine environments (and transition between them) are expected to be at a competitive disadvantage in any individual biome according to the classic adage “jack-of-all-trades, master of none” (Vamosi et al. 2014). Apart from this tradeoff, a generalist strategy could have other fitness costs, such as reduced evolvability (Bono et al. 2020). In macro-organisms, evolutionary transitions between generalism and specialism are thought to occur in both directions but are more commonly directed toward specialism (Nosil 2002; Nosil and Mooers 2005). Recent studies on prokaryotes that classified generalists or specialists based on their distribution across environments also found that evolutionary transitions are directed predominantly toward specialism, but additionally, that generalists possessed higher speciation rates (Sriswasdi et al. 2017; Xu et al. 2022). These results highlight the key role generalists may play in colonizing novel environments and generating microbial diversity (Sriswasdi et al. 2017; Xu et al. 2022).

However informative, the available studies on prokaryote biome transitions have not utilized some of the newly developed comparative phylogenetic methods that can better test whether variation in diversification rates is associated with shifts in a focal trait (in this case “specialist” or “generalist”) (Herrera-Alsina et al. 2019), and have not considered the impact of unsampled diversity resulting in incomplete phylogenies. Not accounting for these factors in analyses can result in false positives (Herrera-Alsina et al. 2019; Chang et al. 2020; Mynard et al. 2023). Furthermore, most studies have relied on the 16S rRNA gene marker, which, although representing the “gold standard” in microbial ecology, offers relatively low genetic resolution and occurs in multiple (sometimes different) copies per genome (Louca et al. 2018). The most commonly used alternative to amplicon sequencing is metagenomic sequencing (Jurdzinski et al. 2023), but because this targets all genes in the microbiome, it uncovers fewer genes belonging to any specific taxon, resulting in missing rarer taxa, which will affect the results of diversification analyses (Moen and Morlon 2014). An alternative option is to use single-copy protein-coding genes, which are reliable proxies for whole-genome divergence (Adékambi et al. 2009) and, with a high rate of evolution, enable differentiation between even closely related taxa (Vos et al. 2012; Caro-Quintero and Ochman 2015) while still allowing exhaustive sampling and retrieval of taxa that would otherwise remain hidden.

Here, we use selective amplification of the *rpoB* gene in Myxobacteria (previously classified as the δ-Proteobacterial Order Myxococcales but recently proposed to form the Phylum Myxococcota (Waite et al. 2020; Parks et al. 2022)), best known for their social development into multicellular fruiting bodies, large genomes, and prolific production of secondary metabolites (Velicer and Vos 2009; Dávila-Céspedes et al. 2016). Myxobacteria have long been known to live across a wide range of terrestrial habitats (Mohr et al. 2017). In the last two decades, they have also been shown to be ubiquitous components of marine (Brinkhoff et al. 2012) and freshwater (Li et al. 2012) sediments. We sequenced the *rpoB* gene in sediment and soil samples collected from different marine and terrestrial environments replicated across Cornwall (UK) and, as has been done in a recent study (von Meijenfeldt et al. 2023), classified biomes not based on a set of abiotic measurements (e.g. pH or salinity), but based on 16S rRNA community composition similarity between samples. In this way, community composition is treated as a proxy for the realized niches of a lineage, reasoning that this reflects both the abiotic environment as well as the biotic environment (formed by interactions between prokaryotes). The presence/absence of Myxococcota ASVs (amplicon sequence variants) across biomes was used to classify each as a specialist or generalist, and we used trait-dependent diversification methods to investigate their macroevolution. Models of discrete character evolution revealed that generalism forms “evolutionary stepping stones” between biome specializations and acts as a source of specialist lineages, with transitions predominantly directed toward specialization. Using the state-dependent speciation and extinction (SSE) framework, we found that diversification rates across the phylogeny varied, but were not associated with biomes or degree of specialization. Our results demonstrate that generalists mediated transitions between biome specialists, who rarely transitioned to specialize on another biome. We also found variation in diversification across the tree, but unlike previous work, this variation was found to be independent of biome association or degree of specialization.

## Results

### Extensive Regional Phylogenetic Diversity of the Phylum Myxococcota Structured Across Three Main Biomes

**Fig. 1.**
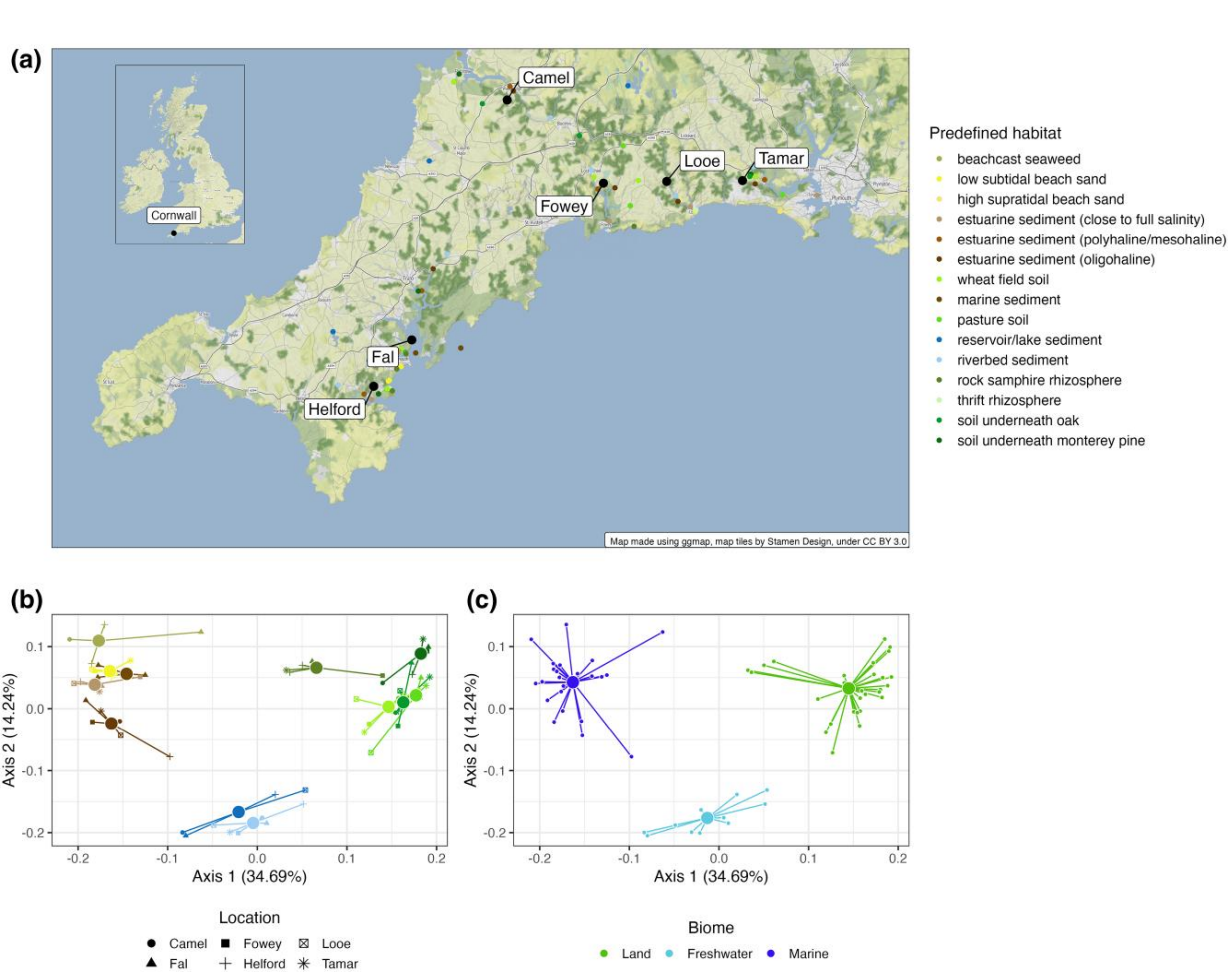
Predefined habitats and biome clusters from our sampling sites across Cornwall, United Kingdom. a) Sampling locations of different predefined habitats across six locations in Cornwall in the southwest of the United Kingdom. b) Principal Coordinate (PCoA) plot of samples based on the weighted-Unifrac distance of the 16S data, with samples colored by their habitat. Samples cluster together based on habitat (different colors), not location (different shapes). c) PCoA plot of samples based on the weighted-Unifrac distance of the 16S data with samples colored by their assignment into a biome cluster based on medoid clustering. The best clustering resulted in three clusters: land (green), marine (dark blue), and freshwater (light blue). In (a), large black points represent broad sampling sites, and small points represent specific sampling sites. In (b) and (c), each small point is an individual sample, large points are the positions of centroids of that group of samples, and lines connect individual samples to the group centroid.

We used targeted sequencing of a ∼225 base pair (bp) region of the *rpoB* gene to uncover Myxobacterial diversity across the county of Cornwall (UK). Fifteen predefined, more or less distinct habitats were sampled across six broad locations with a view to maximizing ecological and phylogenetic diversity, including freshwater, estuarine, and marine sediments, and soils associated with different vegetation or land uses (Fig. 1a; supplementary table S1, Supplementary Material online). After prevalence filtering, 2,621 unique Myxococcota ASVs were identified, compared to a total of only 153 Myxococcota ASVs retrieved from 16S sequencing (a 17-fold increase). The diversity and relative abundance of Myxococcota in individual samples was much higher in the *rpoB* dataset, with an average (mean) of 239 ASVs per sample (minimum = 1, maximum = 789) and a relative abundance of Myxococcota of 0.14 (minimum ≤ 0.001, maximum = 0.47), compared to an average diversity of 42 (one sample had zero Myxococcota) and an average Myxococcota proportion of 0.02 in the 16S dataset, representing a 7-fold increase in Myxococcota sequences. Rarefaction curves demonstrated that diversity was sequenced to sufficient depth across all samples (supplementary fig. S1, Supplementary Material online) and assigning taxonomy using the lowest common ancestor (LCA) method (see Materials and Methods) resulted in 97% of all ASVs being assigned to at least family level. All seven named families in the Myxococcota were retrieved, alongside 16 unidentified families, demonstrating that our primers had phylum-wide coverage.

To determine whether predefined habitats were ecologically distinct, we looked for an overall effect of habitat and location on community composition as quantified by 16S rRNA sequencing (Fig. 1b). Our predefined habitats explained a significant amount of variation in microbial community composition (PERMANOVA, *F*_14,53_ = 13.17, *R*^2^ = 0.76, *P* = 0.001), whereas geographical location, as expected (Vos and Velicer 2008; Louca 2022), did not (PERMANOVA, *F*_5,53_ = 1.14, *R*^2^ = 0.024, *P* = 0.278) (Fig. 1b). To determine which predefined habitats differed significantly in community composition, we ran multiple pairwise permutational ANOVAs (see Materials and Methods) and removed three predefined habitats (high supratidal beach sand, thrift rhizosphere, and estuarine sediment [polyhaline/mesohaline]) that were not significantly different from any of the others. This left us with 12 predefined habitats, each with a significantly different community composition. As having 12 states for our observed trait for comparative analyses is computationally intractable, we used k-medoid clustering to calculate the optimal number of clusters based on the principal coordinate analysis of community composition that corresponded to three main biomes: freshwater (11 samples), marine (25), and land (27) (Fig. 1c).

To assign biome preference to each Myxococcota *rpoB* ASV, we compared their observed prevalence across all biomes to that expected by chance (accounting for the unequal numbers of samples in each biome). Most ASVs (77%) were associated with only one of the three biomes and were designated as either freshwater (738), land (704), or marine (568) specialists. ASVs were designated as generalists when present in multiple biomes at proportions equal to or exceeding those expected by chance. Generalist ASVs were found to be rarer than specialist ASVs (23% of all ASVs). Only six ASVs were found to be associated with all biomes and designated “full” generalists, five ASVs were classified as land + marine generalists, 112 as freshwater + marine generalists, and 488 ASVs as freshwater + land generalists. Therefore, only 123 ASVs (<5%) occurred in both saline and nonsaline environments, which is in line with our expectation that the salinity boundary is challenging to cross (Vermeij and Dudley 2000; Logares et al. 2009; Jurdzinski et al. 2023). This principal finding of biome specialists being most common (and generalists capable of straddling the salt barrier being rare) was consistent across the three OTU cutoffs used (ASVs, 97.7%, and 95% OTU similarity) (supplementary fig. S3, Supplementary Material online).

**Fig. 2.**
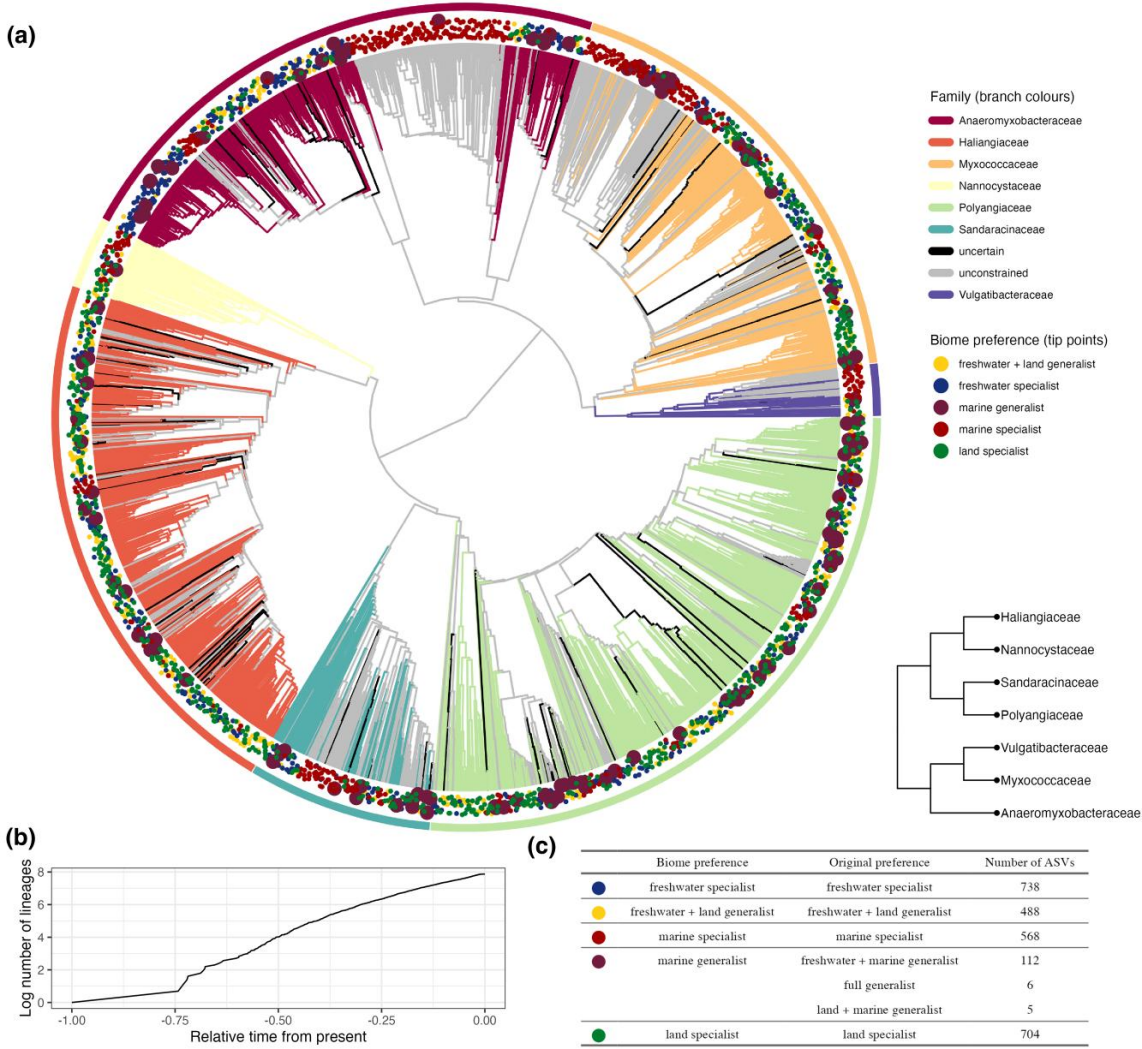
ASV-level constrained phylogeny of Myxococcota sampled in this study. a) Ultrametric phylogenetic tree of Myxococcota based on the rpoB marker. We constrained our phylogenetic tree using a recent Myxococcota multigene phylogeny (bottom right a) and allowed ASVs not assigned to one of the seven families to be unconstrained. Branch colors represent different family taxonomic assignments that we constrained when making the phylogeny; black represents ASVs without a family assignment, and gray represents unconstrained ASVs. Points around the tips of the tree represent biome preference of each ASV. Large points allow easier visualization of marine generalists as they are the least common. b) Lineage-through-time plot for the accumulation of new ASVs through relative time. c) Table showing the classification and abundance of different biome preferences of Myxococcota.

We constructed an ultrametric phylogeny of all Myxococcota ASVs using *raxml-ng* (Kozlov et al. 2019) and *treePL* (Smith and O’Meara 2012) (Fig. 2a), constraining the tree structure based on seven named family-level clades identified in a recent multigene phylogeny (Fig. 2a bottom right) (Waite et al. 2020). Of all ASVs, 73.6% were assigned to these seven families, with the remaining tips being unconstrained during the estimation of the phylogeny. Bootstrapping our phylogenetic tree demonstrated that deeper nodes had relatively high support (values ∼0.75, supplementary figs. S5 and S13, Supplementary Material online), with both low and high bootstrap values found toward the present (supplementary figs. S6 to S13, Supplementary Material online), demonstrating significant phylogenetic uncertainty in parts of the tree (which is not unexpected for a large phylogeny based on a relatively small marker). A lineage-through-time plot (Fig. 2b) demonstrates a steady, near linear accumulation of lineages through evolutionary time on the log scale.

To explore the robustness of our comparative phylogenetics results based on ASVs, we performed the same comparative phylogenetic analyses on nine ASV bootstrap replicates, and the 95% and 97.7% OTU-similarity cutoff trees (hereafter known as 95% tree and 97.7% tree). The nine bootstrapped trees displayed variation in their topology (mean cophenetic distance between best and bootstrap replicate = 0.682, minimum = 0.591, maximum = 0.765) as tips within constrained families could change position and unconstrained tips could move across families. The 95% and 97% trees were much smaller than the ASV tree (1,023 and 1,682 OTUs, respectively), had a similar distribution of bootstrap support values, and smoothing using *treePL* demonstrated a considerable slowdown toward the present, being most pronounced in the 95% tree (supplementary figs. S25 and S26, Supplementary Material online). A slowdown toward the present may be due to our geographically limited sampling meaning we have unsampled Myxococcota diversity resulting in an incomplete phylogeny. After collapsing similar sequences into OTUs this slowdown becomes more exaggerated as clustering removes recent splits from the tree that are mostly generated by the coalescent process, which operates at much smaller timescales and would therefore normally show an acceleration near the present. A further reason for the slowdown is the presence of artifacts introduced by the penalized likelihood method, which is known to underestimate deep node ages when the tree is undersampled, thereby incurring an overall slowdown (Schulte 2013).

### Biome Transitions Are Mediated by Generalists

To explore whether biomes differed in their Myxococcota community composition, we clustered the *rpoB* sequences based on the weighted-Unifrac distance (Lozupone et al. 2011)—which is based on the phylogenetic proximity of species. This demonstrated that freshwater, marine, and land samples had distinct Myxococcota composition (supplementary fig. S4, Supplementary Material online).

**Fig. 3.**
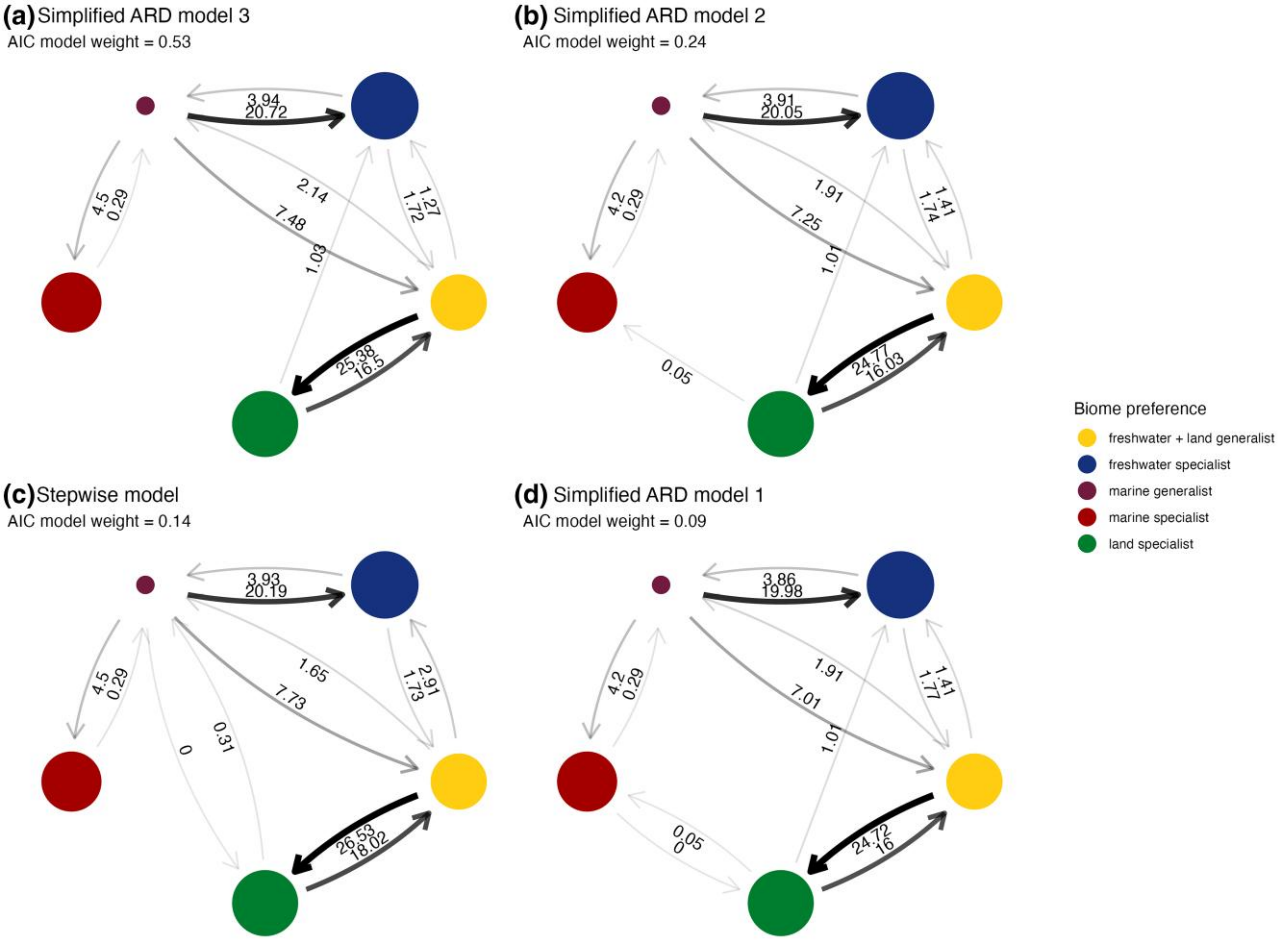
Transition rates between biome preferences for the four best-supported models of discrete character evolution. Three of the best-supported models (a, b, d) were simplifications of the ARD (all-rates-different model) where low transition rates were removed. The third best-supported model (c) was the stepwise model, which did not allow direct transitions between specialist states or marine specialists and freshwater + land generalists. The radius of circles is proportional to the number of ASVs in each biome preference. The size of the arrows is proportional to the transition rate. Transition rates are labeled to two decimal places. All rates are based on a relative (not absolute) time-based phylogeny and should only be interpreted relative to each other.

We next tested whether differences in transition rates between specialists and generalists drove the uneven distribution of ASVs across biome specialists and generalists. As it is difficult to fit comparative phylogenetic models when distributions across states are extremely uneven and when some states have low numbers, we collapsed the three biome preferences with the smallest numbers of ASVs (marine + land generalist, freshwater + marine generalist, and full generalist) into a single preference of “marine generalist” (Fig. 2c). We used Markov models to study discrete character evolution and explore the transitions between biome preferences in the Myxococcota through evolutionary time. We fitted four hypothesis-driven models that restricted some transitional pathways: all-rates-different (ARD), symmetric (SYM), equal rates (ER), and stepwise (SW). The ARD model assumes all transitions are possible and all rates can differ. The SYM model assumes all transitions are possible, but rates to and from any pair of biome preferences are equal, and the ER model assumes all transitions are possible but all occur at the same rate. The SW model assumes that an intermediate generalist state is needed to move to a new specialization (i.e. evolution from marine specialist to land specialist requires a marine + land generalist step first), but all allowed rates can differ. As these are not the only biologically plausible models, we also performed model simplification of the ARD model where we iteratively set transitions with the lowest rates to zero (see Materials and Methods). A custom ARD model of just 11 transitions (of a possible 20) was best supported (Akaike information criterion [AIC] weight = 0.53, Fig. 3a, supplementary table S2, Supplementary Material online), while the estimated transition rates were qualitatively similar among the four best-supported models that cumulatively had an AIC weight of 1 (Fig. 3).

The parameter estimates of the transition models revealed several key patterns in the evolution of biome preference in the Myxococcota. First, it was very rare for specialists of one biome to shift directly to specializing in another biome. The best-supported model only supported one (out of six) specialist to specialist transitions (Fig. 3a), This transition—from land specialist to freshwater specialist—is estimated to occur in three of the four best-supported models (Fig. 3b and c). In two of the best-supported models (Fig. 3b and d), transitions between marine specialist and land specialist are estimated to occur, but at very low rates. In contrast, marine generalists and freshwater + land generalists were the best connected (with six transitions each in the best-supported model, compared to a maximum of five for a biome specialist), acting like stepping stones through which biome specialists evolve (Fig. 3).

Second, generalists are less stable than specialists, with transition rates away from the more generalist state exceeding those towards generalist states (Table 1). For instance, transition rates away from marine generalists are more than five times higher than all rates toward it combined, and freshwater + land generalists have the second highest ratio of rates directed toward compared to away from. In contrast, all specialist states are stable, with transition rates into freshwater, marine, and land specialists being 75%, 94%, and 31% higher than those away from these states (Table 1). Moreover, when looking at individual pairs of transitions, transition rates away from the more generalist state tended to be higher than rates toward it. Although not always the case, this pattern was consistent across all four best-supported models (Fig. 3). Third, marine specialists are extremely stable and the most evolutionarily isolated of all biome preferences, with the fewest connections (a maximum of four connections across the best-supported models) and transition rates both toward and away from this state were the lowest compared to all other biome preferences (Table 1). Fourth, transitions between land specialists and freshwater + land generalists are widespread, indicating that species can easily transition between these biome preferences. We can exclude the possibility that freshwater + land generalists simply represent land specialists transiently present in freshwater sediments due to runoff, as the best-supported Markov model indicates that freshwater + land generalists are more connected to other states than land specialists. Our bootstrapping approaches, where we (i) subsampled 80% of the ASV tree or (ii) subsampled the ASV tree to have the same number of tips within each biome preference followed by re-fitting the best-supported Markov model, gave qualitatively similar results (supplementary figs. S14 and S15, Supplementary Material online). Specifically, transitions away from biome generalists were higher than transitions away from biome specialists, marine generalists were the least stable and marine specialists were the most stable biome preference.

**Table 1:** Total transition rates to and from each biome preference

Biome preference	Away	Into	Source–sink ratio
Marine generalist	32.70	6.37	5.13
Freshwater + land generalist	28.79	25.69	1.12
Land specialist	17.52	25.38	0.69
Freshwater specialist	5.66	23.02	0.25
Marine specialist	0.29	4.50	0.06

To examine the robustness of our results, we redid this analysis on nine ASV bootstrap replicate trees (supplementary figs. S16 to S24, Supplementary Material online), the 95% tree (supplementary fig. S25, Supplementary Material online) and the 97.7% tree (supplementary fig. S26, Supplementary Material online). The best model for all trees was a simplification of the ARD model where low transition rates were removed, but only one estimated exactly the same combination of transitions as in the ASV tree (supplementary table S3, Supplementary Material online and supplementary fig. S24, Supplementary Material online [bootstrap 9]). On average, the best-supported model of bootstrapped trees contained two different transitions compared to the ASV tree (for example, ASV bootstrap 1 supported a low rate from land specialist to marine generalist, and not a transition from land specialist to marine generalist), but qualitatively all patterns remained the same (supplementary fig. S27, Supplementary Material online, supplementary table S3, Supplementary Material online). The bootstrapped trees had an average of 11 transitions, but only ∼1 transition between two specialist states (supplementary table S3, Supplementary Material online). For the 97.7% tree (1,682 tips, supplementary fig. S26, Supplementary Material online), the best-supported model was similar to the ASV-level tree, but with transition rates being estimated to occur from marine specialist to freshwater specialists, and from land specialists to marine specialists. However, these transition rates were low and still resulted in marine specialists being the most stable. For the 95% tree (1,023 tips, supplementary fig. S25, Supplementary Material online), all transitions between freshwater specialist, land specialist, and freshwater + land generalist were supported, and freshwater specialist was the most well connected biome preference. Despite these significant differences in transition rates, marine generalists were the least stable and marine specialists represented the most stable biome preference.

Overall, the results were robust to changes in tree topology and different OTU-similarity cutoffs. Across all trees analyzed, six transitions were estimated to occur across all trees, which were specialist to generalist or generalist to specialist transitions. In contrast, the six specialist to specialist transitions were in the ten (of 20) least prevalent transitions across all trees analyzed (supplementary fig. S27, Supplementary Material online). Two transitions, marine specialist to freshwater + land generalists and marine generalist to land specialist, were never estimated to occur in any of the trees.

### Heterogeneity in Diversification Rates Does Not Vary Between Myxococcota Biome Specialists and Generalists

**Fig. 4.**
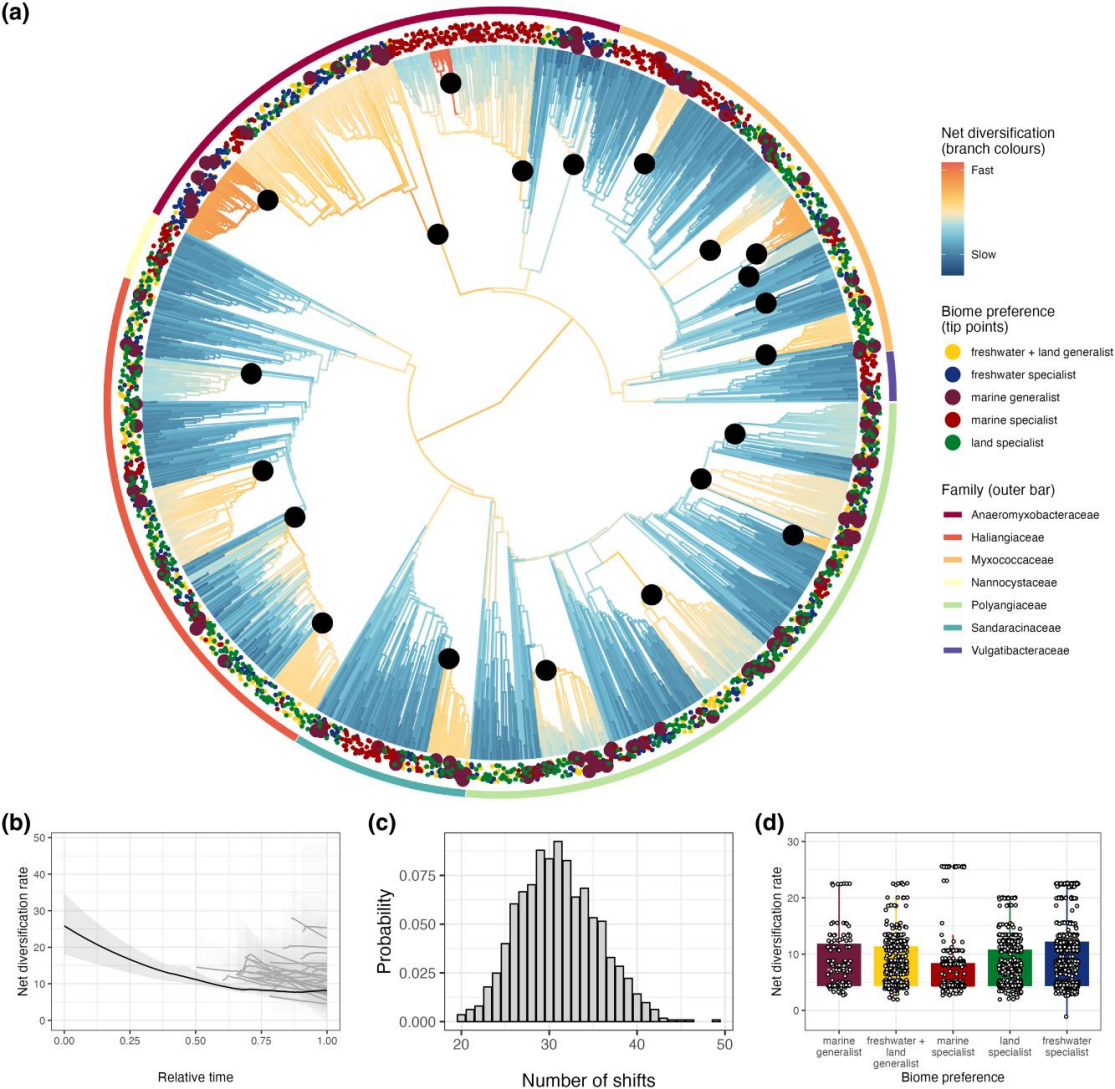
Rate heterogeneity in the diversification of Myxococcota. a) Ultrametric phylogenetic tree of Myxococcota with rates of net diversification inferred using BAMM. Branch colors represent the diversification rate (warmer colors = higher rates). Points around the tips of the tree represent biome preference of each ASV, and bars around the tree represent the family of each clade. Black points represent where rate shifts are estimated to occur based on the best overall shift configuration. b) Rate-through-time plot showing how net diversification decreases over evolutionary time. The black line represents the average across the whole tree; the gray lines represent the rate through time on parts of the tree where core rate shifts were identified. Shaded regions represent 95% confidence intervals. c) Posterior distribution of the number of rate shifts inferred using BAMM. d) Variation in tip-specific diversification rates inferred using BAMM across biome generalists and specialists. All rates are relative (not absolute) and only allow us to look at relative differences between biome preferences and parts of the phylogeny.

We used Bayesian Analysis of Macroevolutionary Mixtures (BAMM) (Rabosky et al. 2014) to detect shifts in diversification rates across the Myxococcota phylogeny (Fig. 4a). Speciation rates across the phylogeny generally decreased over time, while extinction rates remained relatively stable, resulting in a net decrease in diversification rate (Fig. 4b). More important than the average rates across the whole tree, we detected heterogeneity in the diversification rate across the tree, with an average of 31 shifts in the diversification regime (95% credible intervals: 23 to 40) (Fig. 4c). Supporting this, a model with 31 rate shifts in the diversification regime had the highest posterior probability (0.09), and a model with 30 rate shifts had the highest Bayes Factor, with models with 27 to 34 rate shifts all having a Bayes Factor difference within 40. Given the large size of the tree and the range of rate shifts that had similar Bayes Factors, it is unsurprising that the evidence for any one of the 1,070 detected shift configurations was very weak, with the highest percent probability for a single shift configuration being only 0.08%. Consequently, we calculated the best overall shift configuration and estimated the diversification rate through time averaged over the whole tree and for subsets of the tree at the nodes where core shifts occurred during this configuration (see Materials and Methods and Fig. 4a). Immediately after a core shift, diversification rates spiked before receding back toward the global average (Fig. 4a). In summary, we find evidence of heterogeneity in diversification rates across the Myxococcota, but it is impossible to ascertain precisely where, how often, and with what magnitude these shifts are occurring in the phylogeny.

Diversification rate analyses can be sensitive to the unsampled diversity of the phylogeny (Herrera-Alsina et al. 2019; Chang et al. 2020). Due to primer bias and limited geographic sampling, we are unlikely to have sampled the global Myxococcota diversity. To assess how this might impact our results, we ran BAMM on arbitrarily reduced sampling fractions across the whole tree, from the highest value of 100% (assuming a complete phylogeny), to 50% (presented in the main manuscript), 25%, 12.5%, and 6.25%. To test how sensitive our results were to the level of phylogenetic resolution, we also reran the BAMM analysis on the best tree at the 97.7% and 95% OTU cutoff levels. The number of rate shifts inferred by BAMM was not significantly impacted by the changes in sampling fraction (supplementary fig. S28, Supplementary Material online), and the same nodes were regularly identified as being in the best overall shift configuration for each tree (supplementary figs. S29 to S31, Supplementary Material online). However, the number of rate shifts was impacted by the OTU-similarity cutoff, with the 97.7% tree having an average of 14 shifts (supplementary fig. S28, Supplementary Material online), and the majority of models using the 95% tree identifying zero rate shifts (supplementary fig. S28, Supplementary Material online). This indicates that most rate shifts occurred closer to the present, with the 95% clustering resulting in an aggregating of ASVs that masked potential heterogeneity in diversification rate.

To test whether heterogeneity in diversification rate was associated with biome preference, we fitted a set of multistate-dependent speciation and extinction (MuSSE) models that allow diversification rates to vary with biome preference, while accounting for transition rates between these states (FitzJohn 2012). A MuSSE model with only state-dependent speciation rates was selected over models with (i) both state-dependent speciation and extinction, (ii) state-dependent extinction only, and (iii) no state-dependent speciation or extinction (AIC weight = 0.98). This result is consistent with the BAMM analysis that found that heterogeneity in diversification rates was mainly driven by speciation rate. The MuSSE model showed that marine generalists had a higher speciation rate than the other biome preferences. However, SSE analyses must be interpreted with caution as they rest on the assumption that rate heterogeneity is associated with variation in the measured trait (e.g. biome preference) (Rabosky and Goldberg 2015; Herrera-Alsina et al. 2019).

To address this shortcoming, we fitted several models containing “hidden” (or concealed) traits, so-called Hidden State-dependent Speciation and Extinction (HiSSE) models (Caetano et al. 2018; Herrera-Alsina et al. 2019; Nakov et al. 2019). To reduce the number of estimable parameters, we fixed the transition rates between biome preferences to those estimated from the best Markov model. Doing so had little impact on the speciation rate estimates (Pearson's correlation coefficient between speciation rates with and without fixing some transition rates = 0.99), and the correlation between transition rates estimated from the MuSSE model and the Markov model was strong (Pearson's correlation coefficient = 0.93). We refitted the MuSSE model with only state-dependent speciation (supplementary table S7, Supplementary Material online) and compared it to several null models. First, concealed-trait-dependent (CTD) models (Herrera-Alsina et al. 2019) with two, three, or four concealed states (supplementary tables S9 to S11, Supplementary Material online), in which rates of speciation were allowed to vary across lineages modulated by hidden states but not by biome preference. Second, a MuHiSSE model (Nakov et al. 2019) allowing diversification rate variation owing to both hidden variation (two concealed states) and biome preference (supplementary table S8, Supplementary Material online). The CTD4 model (a concealed-trait-dependent model with four hidden states) performed best by far (AIC weight ∼1) with rates broadly similar between all models (supplementary fig. S32a to c, Supplementary Material online).

Again, all models were run on sampling fractions of 100%, 50% (presented here), 25%, 12.5%, and 6.25%. At smaller sampling fractions (12.5% and 6.25%), rates of speciation and extinction increased (supplementary fig. S32a to c, Supplementary Material online), but irrespective of the sampling fraction chosen the concealed-trait-dependent models were favored over MuSSE or MuHiSSE models, with CTD4 performing best at every sampling fraction (supplementary table S4, Supplementary Material online). Repeating the diversification rate analyses on the nine bootstrapped replicate trees revealed the same patterns, with the CTD4 model performing best at every sampling fraction (AIC weight ∼1) (supplementary fig. S32d to f, Supplementary Material online; supplementary table S5, Supplementary Material online), with this pattern also found in the 95% and 97.7% trees (supplementary table S6, Supplementary Material online). In the 95% tree, the CTD4 model performs best despite the BAMM analysis not identifying many rate shifts. Interestingly, the same pattern of rates of speciation and extinction increased at small sampling fractions was not seen at these levels of phylogenetic clustering (supplementary fig. S32g to l, Supplementary Material online). In summary, after accounting for unmeasured biological variation (“hidden states”), there was no evidence for differences in diversification rates between biome specialists or generalists.

## Discussion

In this study, we used an ecologically and geographically explicit, replicated sampling design to explore biome transitions and specialization in the macroevolutionary history of the Myxococcota. Specifically, we used 16S sequencing to cluster predefined habitats into three groups corresponding to the freshwater, land, and marine biomes (Fig. 1c). Most Myxococcota ASVs were biome specialists, with less than 5% of ASVs able to live across the salt barrier (Fig. 2c). We used models of discrete character evolution to investigate the evolution of biome preference in our Myxococcota dataset. Generalists mediated transitions between biomes and then rapidly evolved into specialists, which, while not evolutionary dead ends, generally displayed much lower transition rates “into” rather than “away” compared to generalists (Fig. 3, Table 1). Finally, we performed analyses investigating variation in diversification rates across the Myxococcota and found shifts in diversification rate (Fig. 4), but these shifts were not attributable to biome preference or specific *Myxococcota* clades (Table 2).

**Table 2:** Model comparison of multistate and concealed trait diversification rate models

Model	Number of estimated parameters	Log likelihood	AIC	AIC weight
CTD4	17	190.03	−346.05	1
CTD3	10	174.85	−329.70	0
MuHiSSE	13	137.54	−249.08	0
CTD2	5	121.63	−233.27	0
MuSSE	6	−221.84	455.67	0

The results that rare biome generalists mediate transitions between biomes, with transition rates substantially higher away from, rather than into, generalist states, are consistent with previous findings based on 16S rRNA data (Sriswasdi et al. 2017; Xu et al. 2022; He et al. 2023). There was limited support for transitions between biome specialists, suggesting that biome generalists are able to successfully transition between biomes, after which they rapidly evolve to specialize on one specific biome. Crucially, this result was replicated across all biomes, with transitions into freshwater, land, and marine specialists generally higher than rates in the opposite direction (Fig. 3a). In this way, generalists act like “stepping stone” lineages through which microbes transition between biomes before evolving specialization, somewhat similar to work that found that brackish water biomes act like “stepping stone” environments mediating marine–terrestrial transitions (Jurdzinski et al. 2023).

Not all biome specialists evolve in the same way, with marine specialists being the most evolutionarily isolated, with transition rates into and away from marine specialists the lowest of any state (Table 1). This might reflect more constrained pathways of adaptation from saline to nonsaline environments or possibly more constrained dispersal routes: migration into the marine environment might be more frequent than the other way around, thereby offering more potential for colonizing taxa to adapt to this environment. Our result is consistent with previous work on specific bacterial taxa which uncovered transitions across the salt barrier (Eiler et al. 2016; Simon et al. 2017; Zhang et al. 2019; Ren and Wang 2022; Sereika et al. 2023), where the majority of transitions (∼10) were from the marine to the terrestrial environment, with only two inferred going in the opposite direction. In contrast, dispersal between land and freshwater environments is routine and differences in salinity are much smaller between terrestrial habitats, allowing transitions to happen more readily.

Our analyses did not uncover differences in diversification rates between generalists and specialists associated with different biomes (Table 2). Historically, specialization has been considered an evolutionary dead end, which may result in lower speciation rates and higher extinction rates (McKinney 1997). More recent studies in macro-organisms however have demonstrated that specialists are capable of transitioning back to generalists, and surviving where they do not (Colles et al. 2009). In microbes, two studies found that generalists had (much) higher diversification and speciation rates than specialists (Sriswasdi et al. 2017; Xu et al. 2022), while another study demonstrated the opposite pattern (He et al. 2023). However, none of these studies used Hidden State Speciation and Extinction models, which can account for unknown (hidden) traits that may affect diversification rate (Rabosky and Goldberg 2015; Herrera-Alsina et al. 2019), meaning there is a high risk of false positives in these analyses. In line with this, our MuSSE analysis found that marine generalists had higher speciation rates than other biome preferences, but after using HiSSE models, the best-supported model was one where the diversification rate was independent of biome preference (Table 2).

Similar to previous work investigating bursts in diversification rate in prokaryotes (Morlon et al. 2012; O’Dwyer et al. 2015), we found evidence of rate shifts in diversification (speciation) rates across the phylogeny, but it was not possible to assign such bursts to specific taxa, biomes, or generalist/specialist strategies. Bursts in diversification rate are often interpreted as adaptive radiations, which occur when a single ancestral type encounters (or evolves a key innovation that generates) broad ecological opportunity, enabling diversification into a multitude of specialized types (Schluter 2000). Although their capacity for dispersal means that adaptive radiations in prokaryotes are unlikely to arise via colonization events of novel ecosystems, it may be that the uptake of novel traits through horizontal gene transfer (HGT) allows the colonization of new niche space, which subsequently can be partitioned into different specialists (Vos et al. 2023).

Despite the exponential increase in microbial sequencing data, comparative phylogenetic approaches are still rarely applied to these datasets, and studying the macroevolution of microbes remains challenging both technically and conceptually (Perez-Lamarque et al. 2022). First, the lack of a universally accepted (operational) species definition in prokaryotes impedes the estimation of global diversity and inferring phylogenetic species trees (Vos et al. 2023). Second, while targeted short amplicon sequencing allows for deeper sequencing of microbial diversity, it can be hard to estimate robust phylogenetic trees, whereas the opposite is true for metagenomic data. Both phylogenetic uncertainty and poor estimates of global diversity can affect the results of diversification rate analyses (Moen and Morlon 2014; Perez-Lamarque et al. 2022). Our approach of targeted sequencing of a relatively high resolution gene was able to uncover unprecedented diversity of the prokaryotic Phylum Myxococcota. However, our geographically limited sampling resulted in only partial retrieval of total Myxococcota diversity. The incompleteness of the resulting phylogeny means that some transitions may have been missed and others misidentified. To address this issue, we tested the robustness of our results to uncertainty in tree topology, different assumed levels of unsampled diversity, and different levels of phylogenetic similarity. This revealed that, while specific results and parameter values changed, the overall conclusions remained qualitatively similar. Future research efforts should attempt to better capture global diversity of sampled prokaryote groups.

In summary, we present the first work—to our knowledge—that investigates the macroevolution of both biome transitions and specialization in prokaryotes simultaneously and is amongst the first to apply SSE methods to an observed trait (biome preference) with more than two states. Going forward, combining targeted amplicon sequencing data with (metagenome-assisted) whole-genome data (Jurdzinski et al. 2023) is needed to characterize the role of HGT in evolutionary transitions and its mechanistic impact on ecological specialization (Jaffe et al. 2023). Moreover, increased collaboration between comparative phylogeneticists and microbial ecologists is paramount to ensuring development of methods able to manage the size of microbial sequencing datasets and research macroevolutionary dynamics in prokaryotes.

## Materials and Methods

### Environmental Sampling

We sampled 15 predefined habitats in August 2020 replicated across six drowned river valleys (“rias”) in Cornwall (UK): Helford, Fal, Fowey, Looe, Tamar, and Camel (Fig. 1a). Predefined habitats were riverbed sediment, reservoir/lake sediment, pasture soil, wheat field soil, soil underneath oak, soil underneath monterey pine, rock samphire rhizosphere, marine sediment, low subtidal beach sand, high supratidal beach sand, beachcast seaweed, estuarine sediment (close to full salinity), estuarine sediment (polyhaline/mesohaline), estuarine sediment (oligohaline), and thrift rhizosphere. The number of replicates differed between habitats due to practical limitations, resulting in 73 samples in total (supplementary table S1, Supplementary Material online). Each of the soil or sediment samples consisted of multiple subsamples taken from an area of approximately 0.25 m^2^ to minimize stochastic variation (as it is likely that individual subsamples will contain a variety of (micro)niches (Vos et al. 2013)). Soil samples were taken as shallow as possible after removing leaf litter and were sieved to remove debris. Each sample was stored in two 50 mL falcon tubes and frozen upon return to the lab at −70 °C.

### DNA Extraction and 16S Sequencing

DNA extractions were carried out according to the Qiagen DNeasy PowerSoil kit handbook (1104560 HB-2257-001). A 10-min incubation at 70 °C after the lysis step was included to increase DNA yield. DNA quantity was verified using a picogreen assay (qubit HS DNA kit) (Invitrogen), purity was assessed using nanodrop 260:280 ratios, and integrity was evaluated using a 1% agarose gel. A 251 base pair (bp) conserved fragment in the V4 hypervariable region of the 16S rRNA gene was targeted using N515f and N806r primers (Caporaso et al. 2018) with a pool of indexed primers suitable for multiplex sequencing with Illumina technology. Sequencing was performed using an Illumina MiSeq 500-cycle V2 Kit by the University of Exeter Sequencing Service. After the first sequencing run, four samples had very low depth and were resequenced. Sequencing adapters and any bases below a phred score of Q22 were removed, alongside any reads < 150 bp, using “*Cutadapt*” (v4.4) (Martin 2011). Reads were processed in R (v4.2.2) using the packages “*dada2*” (Callahan et al. 2016) and “*phyloseq*” (McMurdie and Holmes 2013). As error rates differ between sequencing runs, we estimated trimmed reads, estimated error rates, and inferred and merged sequences separately. While processing the first sequencing run, we trimmed the first 10 bp off and truncated all reads at 225 bp for both the forward and reverse samples. For the four resequenced samples, we trimmed the first 10 bp off the forward and reverse reads and then truncated forward reads at 265 bp and reverse reads at 225 bp. We then merged the two sequence tables (which joined together any ASVs present across sequencing runs), removed chimeric sequences, and assigned taxonomies to ASVs using the SILVA database (v138.1) (Quast et al. 2012). We estimated a phylogeny using “*fasttree*” using the Jukes–Cantor + CAT model on the nucleotide alignment (Price et al. 2010). Any ASVs that (i) were over 250 bp in length, (ii) had not been assigned to at least Phylum level, (iii) appeared in <5% of all samples, and (iv) had a total abundance of <200 across the whole dataset were removed. Overall, this left 6,030 individual ASVs across 73 samples encompassing the 15 habitats that were included in downstream analyses, with an average of 58,681 reads per sample, a minimum of 12,570 reads and a maximum of 174,902 reads.

### rpoB Amplicon Primer Design and Sequencing

Group-specific primers targeting the Myxobacteria (GTDB Phylum Myxococcota) were designed using the R package DECIPHER (Wright 2016). Firstly, all genomes assigned to the phylum Myxococcota from the NCBI (Kitts et al. 2016) and GTDB (r202) (Parks et al. 2022) databases were downloaded using “*ncbi-genome-download*” (Blin 2023) to extract the *rpoB* gene sequence. We removed identical sequences, kept only sequences between 3,900 and 4,400 bp in length, and ensured there was only a single copy of *rpoB* per genome (keeping the sequence closest to the median length of the gene). Finally, we manually removed five sequences that aligned especially poorly to the others. The remaining 158 sequences were aligned using “*DECIPHER::AlignTranslation()*,” resulting in a 4,641 bp alignment (Wright 2015). Outgroup sequences were chosen by re-rooting the GTDB phylogeny (r202: https://data.gtdb.ecogenomic.org/releases/release202/202.0/bac120_r202.tree) to the origin of the Myxococcota and selecting the 3,000 accessions that had the shortest distance to this node (i.e. the bacteria most closely related to the Myxococcota). The genomes for these accessions were downloaded, and the *rpoB* gene sequence aligned as described above, but in addition, we removed sequences that had a median distance (from the other outgroup sequences) of over 0.4 and a distance from a reference *Myxococcus xanthus* DK 1622 sequence of over 0.35. This resulted in 164 non-Myxococcota sequences and a 4,689 bp alignment.

Both alignments were combined using “*DECIPHER::AlignProfiles()*” to create a 322 sequence, 5,060 bp alignment of Myxococcota and non-Myxococcota sequences. Primers were designed using “*DECIPHER::DesignPrimers()*.” No selective primers for long amplicons could be designed, so we limited our search to a predicted product size between 200 and 400 bp. Several candidate primers were tested on genomic DNA of *Nannocystis exedens*, *Bradymonas sediminis*, and *Corallococcus coralloides* (purified gDNAs purchased from the Leibniz Institute DSMZ Braunschweig, Germany). We also tested these primers on gDNA extracted from a random sample of river sediment using our chosen purification method. The primer pair producing a single strong product for all test samples was selected from the candidate list. Our final primers for targeted Myxococcota *rpoB* sequencing were GCGATCAAGGAGCGCATG-F and CAGATGCGGCCGTAGTG-R. This primer set had a predicted amplicon size of ∼260 bp, and was predicted to amplify 78% of the Myxococcota sequences in our alignment and only 5% of the non-Myxococcota sequences. We created phased primer pairs to sequence 63 samples; samples from high supratidal beach sand, thrift rhizosphere, and estuarine sediment (polyhaline/mesohaline) were removed as they did not differ in composition from the majority of other predefined habitats. Sequencing was done on an Illumina Novaseq on 28/09/2021 with paired-end 250 bp reads by the Exeter Sequencing service. Primers were removed, and reads were dephased before being processed using “*dada2*” and “*phyloseq*.”

First, forward and reverse reads were truncated at 200 bp. The Novaseq sequencing run returned binned quality scores, which meant the estimated error rates at the highest quality score were higher than those at intermediate quality scores. To overcome this, we enforced monotonicity to the error model by changing the arguments of the loess model to have a span equal to 2 and weights equal to the log-transformed total counts of nucleotides (https://github.com/benjjneb/dada2/issues/1307). We then inferred and merged sequences, constructed a sequence table, and assigned taxonomy using a reference database of all *rpoB* sequences in the GTDB database (r202). This pipeline resulted in a 222 bp *rpoB* amplicon, 494,114 unique ASVs and a mean read number of 1,570,304 (minimum = 220,231, maximum = 7,427,083). We filtered this dataset to solely retain ASVs assigned to any of the Myxococcota phyla in the r202 GTDB database (*Myxococcota*, *Myxococcota_A*, and *Myxococcota_B*).

To cross-validate the naive Bayesian classifier implemented in *dada2*, the taxonomy of all sequences identified as Myxococcota was also assigned using LCA algorithms as implemented by “*MMSeqs2*” (Steinegger and Söding 2017). After building a custom database from the GTDB *rpoB* fasta file, taxonomy was assigned using the default LCA algorithm (*mmseqs taxonomy --lca-mode 3*), selecting the most specific taxonomic label that had at least 95% support (*--majority 0.95*) of the −log(*E*-value) weights (*--vote-mode 1*). Additional arguments set were: assigning taxonomy to nucleotide sequences (*--search-type 3*), returning all lineage information in the output (*--tax-lineage 1*), and disabling pre-filtering query ORFs (*--orf-filter 0*). This resulted in the removal of 76 ASVs (0.17%) not assigned to Myxococcota. There was very good congruence in the assignments between methods, but the LCA method assigned more ASVs down to family level. Consequently, we used the LCA taxonomic assignment in downstream analyses. Prevalence filtering removed all *rpoB* ASVs occurring in fewer than four samples and with a total abundance of fewer than 100 reads. After these filtering steps, there were 2,621 individual ASVs, and samples had a mean read number of 87,948 (minimum = 28, maximum = 340,355).

To check whether patterns were different at different levels of phylogenetic relatedness, we clustered our ASV dataset at two levels of OTU similarity: 97.7% (previously identified as a suitable species boundary cutoff for *rpoB* (Vos et al. 2012)), and 95% (the commonly used cutoff for the species boundary using 16S amplicon sequencing). The 2,621 unique sequences were aligned using “*DECIPHER::AlignSeqs()*” using a guide tree, and the distance matrix was calculated using “*DECIPHER::DistanceMatrix()*,” which calculates the Hamming distance between each of the sequences in the alignment. For each OTU-similarity cutoff, we clustered the sequences from the distance matrix using “*DECIPHER::TreeLine()*” and used “*speedyseq::merge_taxa_vec()*” (McLaren 2020) to merge clusters into single OTUs (using the name and sequence for the most abundant ASV in the cluster to represent the new clustered OTU).

For each dataset, we estimated a phylogenetic tree using *raxml-ng* (v1.1.0) (Kozlov et al. 2019). We used a recent multigene phylogenetic tree of the Myxococcota (Waite et al. 2020) to create a constraint tree, ensuring that any ASVs assigned to the families Myxococcaceae, Vulgatibacteraceae, Anaeromyxobacteraceae, Polyangiaceae, Sandaracinaceae, Nannocystaceae, and Haliangiaceae were placed within the same clade, and relationships between families were fixed based on the topology of the multigene tree. ASVs not assigned to one of these families were left unconstrained. We used the GTR + gamma model and ran 20 tree searches (ten random and ten parsimony-based starting trees), and the best tree was chosen based on the maximum likelihood topology. The best tree for each level of phylogenetic relatedness (ASV, 97.7%, and 95%) was rooted manually in FigTree (Rambaut 2012) by finding the split between the two Classes (Myxococcia or Polyangia) specified in the constraint tree. Trees were bootstrapped with *raxml-ng* to convergence using the “*autoMRE*” convergence test with a maximum of 500 replicates (Pattengale et al. 2010), and the transfer bootstrap expectation (Lemoine et al. 2018) was calculated. Trees were made ultrametric using *treePL* using cross validation (Smith and O’Meara 2012).

### Statistical Analyses

#### Analyzing Microbial Community Composition and Clustering Samples Into Biomes

To test whether our predefined habitats differed in community composition, we employed both supervised and unsupervised clustering analyses on the relative abundances of the 16S ASVs using weighted-Unifrac distance (Lozupone et al. 2011). First, we ran a permutational ANOVA to test whether habitat or location had significant impacts on community composition using “*vegan::adonis2()*” (Oksanen et al. 2007) with 9,999 permutations. Following this, we ran pairwise permutational ANOVAs between all pairs of predefined habitats to test which were significantly different from each other. This was done by subsetting the data into pairwise combinations of habitats, running a permutational ANOVA on each subset, and extracting the *R*^2^ value and *P*-value, which was adjusted using the false discovery rate (FDR) method (Benjamini and Hochberg 1995). The only nonsignificant contrasts involved samples from high supratidal beach sand, thrift rhizosphere, and estuarine sediment (polyhaline/mesohaline), which were removed from subsequent analyses.

Predefined habitats were then clustered into broad biomes using unsupervised learning, with the dissimilarity matrix created from multidimensional scaling of the weighted-Unifrac distance matrix used as the input and limiting the maximum dimensions of the space of the matrix to only include positive eigenvalues. k-medoid and hierarchical clustering methods were used to estimate the number of clusters that best grouped the data using two approaches. First, we used k-medoid clustering using “*cluster::clusGap()*” (Maechler et al. 2012) at every level of possible clustering (from 1 to 12—the number of predefined habitats). The optimal number of clusters was calculated using the gap statistic and their standard deviations, using Tibshirani's recommendation (Hastie et al. 2001; Maechler et al. 2012). Second, we used k-means clustering and “*NbClust::NbClust()*” (Charrad et al. 2014), which calculates 30 indices and recommends the optimal number of clusters using the majority rule. We also used the gap statistic and the majority rule approaches to determine the optimal number of clusters using hierarchical clustering, where we used the ward method in “*clusGap()*.”

All four combinations of clustering (k-medoid and hierarchical) and methods to determine optimal cluster numbers (gap statistic and majority rule) assigned samples to three clusters (freshwater, land, and marine). The single difference was that both hierarchical clustering methods assigned one sample of beachcast seaweed to the land cluster, whereas all beachcast seaweed samples were assigned to the marine cluster using k-medoid clustering methods. As it makes sense for all samples within a predefined habitat to be clustered within the same biome, the samples were assigned to clusters using the k-medoid clustering method, with the gap statistic and majority rule approaches giving identical results.

#### Assigning Biome Preference to Myxococcota ASVs

The presence/absence of each Myxococcota ASV across the three biomes (freshwater, marine, or land) was used to assign biome preference. Any ASV that was only present in a single biome was designated as a biome specialist. For any ASV present in two or three biomes, we employed a bootstrapping approach to assign biome preference. Specifically, we created a bootstrapped presence dataset for each ASV by sampling their observed presence across samples 100 times with replacement to calculate their proportional presence across biomes. This process was repeated 1,000 times for each ASV to create a distribution of observed biome preference proportions. We then compared these observed proportions to those based on the number of samples in each biome (land = 0.44, marine = 0.38, freshwater = 0.18), akin to habitat availability. For every ASV, if just 2.5% of the observed use estimates in any given biome were above the expected proportion given its availability, we assumed it had an affinity for that biome (supplementary fig. S2, Supplementary Material online). Consequently, ASV biome preference consists of all the biomes where the ASV was present at a level at least as high as expected by each biome's availability (supplementary fig. S2, Supplementary Material online). This approach meant that seven different biome preferences were possible (freshwater specialist, marine specialist, land specialist, freshwater + marine generalist, marine + land generalist, freshwater + land generalist, and full generalist [i.e. land, freshwater, and marine]). Biome preference was assigned separately to each ASV at each OTU-similarity cutoff.

#### Investigating the Evolution of Biome Preference Using Models of Discrete Character Evolution

We modeled the evolution of biome preference using Markov models. We used “*diversitree::fit_mk()*” (FitzJohn 2012) which can handle multistate traits and estimate transition rates among different states. As the numbers of ASVs that were full generalists or marine + land generalists were extremely low, we merged these with freshwater + marine generalists to create a marine generalist group. We fitted four hypothesis-driven models that restricted some transitional pathways: all-rates-different (ARD), symmetric (SYM), equal rates (ER), and stepwise (SW). As our trait is simply an association, these are not the only biologically plausible models. Consequently, we also performed iterative model simplification on the ARD model to set the lowest transition rate to zero until AIC stopped decreasing. For the first simplified model, we set four transitions that were less than 0.001 to 0 and then set the single smallest transition rate to zero for each subsequent model simplification. We then compared all models using AIC weights (Burnham and Anderson 2002). For the best-supported model, we calculated a “source–sink ratio” by dividing the sum of the transition rates into a biome preference by the sum of the transition rates away from the same biome preference. We estimated the uncertainty in transition rates of the ASV tree using two bootstrap approaches. First, we subsampled the tree to 80% of its full size and refitted the best-supported model. Second, we subsampled the number of tips assigned to each biome preference to have the same number of ASVs (123) and refitted the best model. We did both approaches for 1,000 iterations and then calculated mean estimates and 95% confidence intervals for transition rates and “source–sink ratios”.

#### Exploring Heterogeneity in Diversification Rates of Myxococcota Using BAMM

We used BAMM to estimate speciation and extinction rates and identify rate shifts in net diversification across our Myxococcota phylogeny (Rabosky et al. 2014). BAMM uses reversible-jump Markov chain Monte Carlo sampling to explore shifts in macroevolutionary regimes, assuming they occur across branches of a phylogeny under a compound Poisson process. It explicitly explores diversification rate variation through time and among lineages. Priors for BAMM were generated using the R package “*BAMMtools*” (Rabosky et al. 2014) and the expected number of transitions was set to 500 to aid convergence (Mitchell and Rabosky 2017). We ran four MCMC chains for at least 30,000,000 generations, allowing chain swaps every 1,000 generations and saving output every 20,000 generations. We assessed convergence by calculating the effective sample size (ESS) of the log likelihood and the number of shifts of the results after a burn-in period of 25% (ESS values > 200 are indicative of good convergence). We also checked that the posterior of the number of transitions differed from the prior by using “*BAMMtools::plotPrior()*.” Diversification rate analyses require an estimate of the completeness of the phylogeny. We used five different sampling fractions spanning a wide range to test how different amounts of assumed unsampled Myxococcota diversity impacted results. We ran BAMM with sampling fractions of 1 (assuming we had no missing extant tips), 0.5, 0.25, 0.125, and 0.0625.

For each BAMM run, the best overall model (number of rate shifts) was chosen by selecting the model with the highest Bayes Factor relative to the null model, which has zero rate shifts. We calculated the credible shift set—the ranked set of distinct shift configurations that accounts for 95% of the posterior probability of the data—for our BAMM analysis. This returns the number of core shifts, defined as those that contribute appreciably to our ability to model the data. In contrast, noncore shifts are simply shifts we would expect to sometimes happen under the prior distribution for rate shifts across the tree. In our case, all shift configurations had very low probability (the best having a posterior probability of 0.0019). This is expected in some datasets with large numbers of taxa as there are simply too many parameters in the model to allow a single shift configuration to dominate the credible set. Consequently, we extracted the shift configuration using “*maximumShiftCredibility*” that maximizes the marginal probability of rate shifts along individual branches, similar to the maximum clade credibility tree in phylogenetic analysis.

#### State-Dependent Diversification Analysis and Parameterization

We used MuSSE models (FitzJohn 2012) to determine whether rate heterogeneity is associated with biome preference. In these models, a lineage's speciation or extinction rate depends on biome preference, and transitions between biome preferences were limited to those from the best-supported transition matrix from the Markov models. We first used “*diversitree::fit_mk()*” to compare models where (i) both speciation and extinction were associated with biome preference, (ii) only speciation was associated with biome preference, (iii) only extinction was associated with biome preference, and (iv) neither speciation nor extinction was associated with biome preference (constant-rate model). The sampling fraction was 50% and models were compared using AIC weights.

It is possible that the SSE model could be supported over a constant-rate model just because it allows for variation in speciation (or extinction) rate across the tree (Rabosky and Goldberg 2015; Herrera-Alsina et al. 2019). Consequently, we fitted models where diversification rates depend on an unknown (hidden or concealed) trait using the R package “*secsse*” (Herrera-Alsina et al. 2019). For all models, we estimated a single extinction rate and fixed transitions between biome preferences to those estimated from the best Markov model to limit the number of estimable parameters. The correlation between the transition rates of a MuSSE model with free transition rates and fixed rates using *diversitree* was 0.93. The correlation between speciation rates of a MuSSE model with free transition rates and fixed rates using *diversitree* was 0.98. The correlation between speciation rates of the same MuSSE model with fixed transition rates in *diversitree* and *secsse* was 0.99. We fitted five different models: (i) a MuSSE model with no hidden states (supplementary table S7, Supplementary Material online), (ii) a MuHiSSE model that allowed for both state-dependent and hidden state speciation rates (supplementary table S8, Supplementary Material online), (iii) a concealed trait diversification model (CTD) with two (CTD2, supplementary table S9, Supplementary Material online), (iv) three (CTD3, supplementary table S10, Supplementary Material online), or (v) four hidden states (CTD4, supplementary table S11, Supplementary Material online). For models including hidden states, transitions to and from the same hidden state were allowed to differ (i.e. 1A → 1B ≄ 1B → 1A), and dual transitions were disallowed (i.e. could not move hidden and measured traits at once). Models were compared using AIC scores and AIC weights. Statistical support for biome preference affecting diversification rates was found when the AIC score of the model in which speciation (or extinction) differs across biome preferences was higher than that in which rates depend on an unknown (CTD model) and a constant-rate model.

We used several different initial parameter sets to circumvent local optima during likelihood optimization with “*secsse*.” The first set of parameters were the estimates of speciation and extinction from a birth–death model fit to the branching times and transition rates from the best-supported Markov model using “*DDD::bd_ml()*” (Etienne et al. 2023). For transitions between hidden states, the initial start value was the mean of the transition rates from the best-supported Markov model. We then created a grid of all combinations of starting values for half and double these initial values (27 different combinations) and calculated the log likelihood of the model using these estimates given the data using “*secsse::secsse_loglik()*.” We then chose starting parameters with the six highest initial log-likelihood values to fit to the data using “*secsse::secsse_ml()*,” retaining the model with the highest likelihood. We reran the model-fitting process at five different levels of sampling fraction (1, 0.5, and 0.25, 0.125, and 0.0625) to determine how sensitive our conclusions are to the assumption that we have sampled all the Myxococcota diversity in the samples. All *secsse* models were fitted with 75 optimization cycles, the *simplex* algorithm, and the default *bulirsch_stoer* algorithm, with a log-likelihood penalty of 0.1 to prevent unrealistically high parameter estimates and to aid in model fitting.

#### Exploring the Robustness of Results

To investigate how robust our results were to changes in tree topology (e.g. phylogenetic uncertainty) and choice of phylogenetic clustering (e.g. species delineation), we tested how our analyses changed when using (i) nine different bootstrapped replicates of the ASV-level tree and (ii) the best tree of the 95% (the cutoff traditionally used to assign “species” at the microbial level) and 97.7% (which we previously identified as a cutoff to assign species using the rpoB gene (Vos et al. 2012)) OTU-similarity cutoffs. Nine bootstrapped replicates were randomly chosen from the ASV tree, rooted using FigTree, and made ultrametric using *treePL* as above. For the bootstrapped trees, we reran the Markov models to investigate discrete character evolution, and the state-dependent diversification analyses looking at whether variation in speciation rate was best supported by biome preference, hidden/concealed traits, or a combination of the two. For the 97.7% and 95% trees, we redid the same analyses as for the ASV bootstraps, but also redid the diversification rate analyses using BAMM. When doing the BAMM analysis, no bootstrap replicate trees of the 95% and 97.7% OTU-similarity trees were used.

### Overview of Open Source Software Used

All R scripts used elements of the suite of packages known as the “*tidyverse*” (Wickham et al. 2019), all phylogenetic trees were plotted using “*ggtree*” (Yu et al. 2017), all figures were made using “*ggplot2*” (Wickham 2016), and all tables were created using “*flextable*” (Gohel et al. 2021). Specific R packages are referenced in their relevant section, and scripts for most parts of the analysis and to recreate all the plots created in the manuscript are available.

## Supplementary Material

msae088_Supplementary_Data

## Data Availability

All raw sequencing data has been deposited on the European Nucleotide Archive (Study Accession number: PRJEB73761). Processed data and code to recreate all analyses are publicly available on GitHub (https://github.com/padpadpadpad/myxo_diversification) and is archived on Zenodo (https://zenodo.org/doi/10.5281/zenodo.11210843). The analysis code starts with the processed *phyloseq* objects created after the *dada2* workflow. We thank two reviewers for their extensive and detailed comments that significantly improved the analyses.
