# Supplementary material for: Macroevolutionary Dynamics in Micro-organisms: Generalists Give Rise to Specialists Across Biomes in the Ubiquitous Bacterial Phylum Myxococcota
Source: Mol Biol Evol. 2024 May 8;41(5):msae088. doi: 10.1093/molbev/msae088 (PMC11127111; doi:10.1093/molbev/msae088)
Supplement: msae088_Supplementary_Data [file msae088_supplementary_data.pdf]

Supplementary Information for: Macroevolutionary dynamics in micro-organisms: generalists give rise to specialists across biomes in the ubiquitous bacterial phylum *Myxococcota*.

Daniel Padfield[1], Suzanne Kay[1], Rutger Vos[2,3], Christopher Quince[4,5], and Michiel Vos[1,6]

[1]Environment and Sustainability Institute, Penryn Campus, TR10 9FE, UK
[2]Naturalis Biodiversity Center, P.O. Box 9517, 2300 RA, Leiden, The Netherlands
[3]Institute of Biology Leiden, Leiden University, Sylviusweg 72, 2333 BE, Leiden, The Netherlands
[4]Organisms and Ecosystems, Earlham Institute, Norwich, Norwich, NR4 7UZ, UK
[5]Gut Microbes and Health, Quadram Institute, Norwich, NR4 7UQ, UK
[6]European Centre for Environment and Human Health, Penryn Campus, TR10 9FE, UK

Corresponding Author: Daniel Padfield

Table S1. Summary of samples, sites, predefined habitats, and sequencing.

sample number	site	location	latitude	longitude	predefined habitat	16s sequencing	rpoB sequencing
60	Camel	Doom Bar by Hawker's Cove	50.56247	-4.944736	beachcast seaweed	yes	yes
28	Fal	Swanpool Beach	50.14070	-5.076021	beachcast seaweed	yes	yes
70	Fal	Flushing	50.16126	-5.056420	beachcast seaweed	yes	yes
72	Helford	beach near Rosemullion Head	50.10760	-5.087167	beachcast seaweed	yes	yes
30	Fal	Penryn river Flushing	50.16445	-5.071440	estuarine sediment (close to full salinity)	yes	yes
38	Fowey	Bodinnick ferry	50.33983	-4.630327	estuarine sediment (close to full salinity)	yes	yes
16	Helford	Helford Passage Beach	50.09993	-5.128802	estuarine sediment (close to full salinity)	yes	yes
23	Looe	Millpool pond	50.35870	-4.459996	estuarine sediment (close to full salinity)	yes	yes
52	Tamar	The Old Rowing Club Torpoint	50.37396	-4.193850	estuarine sediment (close to full salinity)	yes	yes
64	Camel	Wadebridge, Guineaport Road	50.51337	-4.831025	estuarine sediment (oligohaline)	yes	yes
55	Fal	Tresillian River, Audi Tresillian	50.27536	-4.999346	estuarine sediment (oligohaline)	yes	yes
37	Fowey	Lerryn	50.38409	-4.617869	estuarine sediment (oligohaline)	yes	yes
14	Helford	Gweek Bridge	50.09638	-5.208347	estuarine sediment (oligohaline)	yes	yes
20	Looe	Watergate camping West Looe	50.36583	-4.485670	estuarine sediment (oligohaline)	yes	yes
53	Tamar	field near Polbathic Village Hall	50.38930	-4.323641	estuarine sediment (oligohaline)	yes	yes
63	Camel	Wadebridge, Bridge Bike hire	50.51930	-4.838130	estuarine sediment (polyhaline/mesohaline)	yes	no
10	Fal	Malpas Road pontoon	50.24595	-5.023255	estuarine sediment (polyhaline/mesohaline)	yes	no
41	Fowey	Saint Winnow	50.38258	-4.653494	estuarine sediment (polyhaline/mesohaline)	yes	no
15	Helford	Port Navas	50.10716	-5.144270	estuarine sediment (polyhaline/mesohaline)	yes	no
47	Tamar	end of Old Quay Lane	50.39482	-4.303952	estuarine sediment (polyhaline/mesohaline)	yes	no
19	Helford	Maenporth	50.12460	-5.093505	high supratidal beach sand	yes	no
44	Tamar	Tregantle Beach/Whitsand Bay	50.35302	-4.270939	high supratidal beach sand	yes	no
59	Camel	Doom Bar by Hawker's Cove	50.56247	-4.944736	low subtidal beach sand	yes	yes
27	Fal	Gylly Beach	50.14412	-5.066652	low subtidal beach sand	yes	yes
18	Helford	Maenporth	50.12570	-5.091337	low subtidal beach sand	yes	yes
24	Looe	East Looe Beach	50.35282	-4.449455	low subtidal beach sand	yes	yes
42	Tamar	Tregantle Beach/Whitsand Bay	50.35249	-4.271751	low subtidal beach sand	yes	yes
65b	Fal	Outer Bizzies (West)	50.16904	-4.941416	marine sediment	yes	yes
66	Fal	Outer Bizzies (East)	50.16904	-4.941416	marine sediment	yes	yes
67	Fal	East Narrows	50.16247	-5.036010	marine sediment	yes	yes
68	Fal	East Narrows	50.16247	-5.036010	marine sediment	yes	yes
57	Camel	road to Tregella Farm	50.52576	-4.955687	pasture soil	yes	yes
2	Fal	Trefusis Head	50.16199	-5.057825	pasture soil	yes	yes
40	Fowey	near Valleybrook	50.36016	-4.585863	pasture soil	yes	yes
5	Helford	Maenporth Road	50.11345	-5.096796	pasture soil	yes	yes
34	Looe	Wayton Barn	50.44024	-4.600625	pasture soil	yes	yes
46	Tamar	Wacken Quay	50.37494	-4.266530	pasture soil	yes	yes
65	Camel	Porth Reservoir	50.42000	-5.007679	reservoir/lake sediment	yes	yes
69	Fal	College Reservoir	50.15516	-5.129523	reservoir/lake sediment	yes	yes
9	Fal	Argal Reservoir	50.15207	-5.133878	reservoir/lake sediment	yes	yes
12	Helford	Stithians Reservoir	50.19099	-5.209554	reservoir/lake sediment	yes	yes
33	Looe	Colliford Lake	50.52035	-4.590757	reservoir/lake sediment	yes	yes
56	Camel	Camel River Dunmere Bridge	50.47749	-4.753194	riverbed sediment	yes	yes
54	Fal	Tresillian River	50.29501	-4.970236	riverbed sediment	yes	yes
35	Fowey	Tudor Bridge	50.40752	-4.666920	riverbed sediment	yes	yes
13	Helford	Helford Stream	50.11960	-5.198810	riverbed sediment	yes	yes
22	Looe	West Looe River	50.37369	-4.490573	riverbed sediment	yes	yes
51	Tamar	River Tiddy	50.41212	-4.327172	riverbed sediment	yes	yes
71	Fal	Flushing	50.16126	-5.056420	rock samphire rhizosphere	yes	yes
29	Fal	Swanpool Beach	50.14104	-5.075885	rock samphire rhizosphere	yes	yes
39	Fowey	Lansallos Cove	50.33249	-4.577888	rock samphire rhizosphere	yes	yes
73	Helford	Rosemullion Head	50.11167	-5.085058	rock samphire rhizosphere	yes	yes
43	Tamar	Tregantle Beach/Whitsand Bay	50.35302	-4.270939	rock samphire rhizosphere	yes	yes
62	Camel	Padstow School sports field	50.53603	-4.945018	soil underneath monterey pine	yes	yes
11	Fal	St. Clement/Malpas	50.24548	-5.030602	soil underneath monterey pine	yes	yes
31	Fowey	Lanhydrock	50.45368	-4.692297	soil underneath monterey pine	yes	yes
17	Helford	Bosveal car park	50.10749	-5.114093	soil underneath monterey pine	yes	yes
49	Tamar	Saltash	50.40236	-4.333044	soil underneath monterey pine	yes	yes
61	Camel	Atlantic Highway lane	50.49642	-4.896110	soil underneath oak	yes	yes
1	Fal	Kilnquay Wood	50.16315	-5.058057	soil underneath oak	yes	yes
32	Fowey	Lanhydrock	50.45405	-4.692600	soil underneath oak	yes	yes
6	Helford	Maenporth Road	50.11400	-5.093901	soil underneath oak	yes	yes
21	Looe	West Looe river bank	50.37369	-4.490573	soil underneath oak	yes	yes
50	Tamar	Saltash, Bag Lane	50.39924	-4.335000	soil underneath oak	yes	yes
3	Fal	Flushing Beach	50.16126	-5.056420	thrift rhizosphere	yes	no
7	Helford	Nansidwell	50.11475	-5.091429	thrift rhizosphere	yes	no
25	Looe	East Looe Beach (rocks on the east side)	50.35444	-4.448103	thrift rhizosphere	yes	no
48	Camel	Saint Germans	50.39948	-4.323441	wheat field soil	yes	yes
4	Fal	Flushing	50.16777	-5.066961	wheat field soil	yes	yes
36	Fowey	near Moorfield	50.39811	-4.662053	wheat field soil	yes	yes
8	Helford	Maenporth Road	50.11407	-5.095674	wheat field soil	yes	yes
26	Looe	Lanreath	50.39391	-4.568772	wheat field soil	yes	yes
58	Tamar	road to Tregella Farm	50.52539	-4.956342	wheat field soil	yes	yes

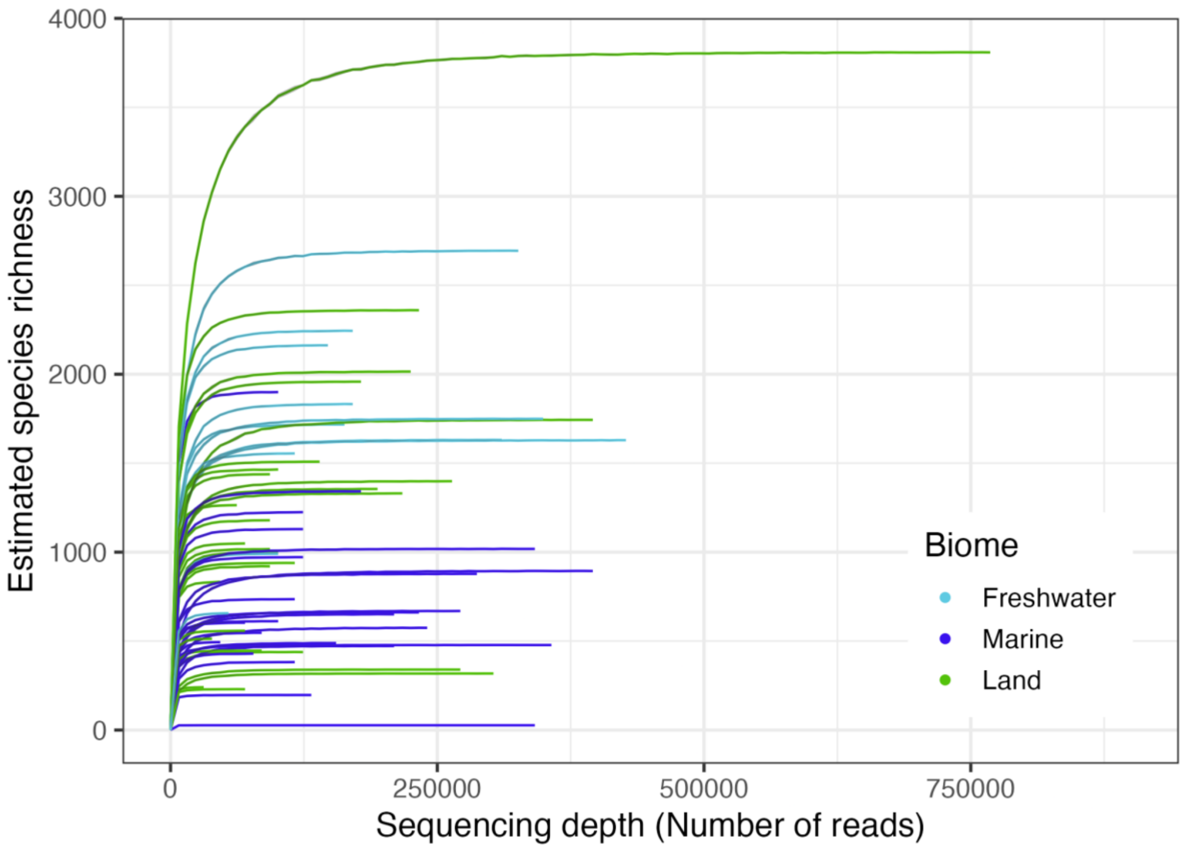

Figure S1. Rarefaction curves of rpoB sequencing. Rarefaction curves to check sufficient sequencing depth were estimated for each rpoB sequencing sample after filtering to keep only ASVs assigned to the *Myxococcota*. For each sample, rarefaction was constrained to the number of reads in each sample. The relationship between sequencing depth and estimated species richness has plateaued in all samples, indicating we have captured all the *Myxococcota* diversity in the sample (given the primers).

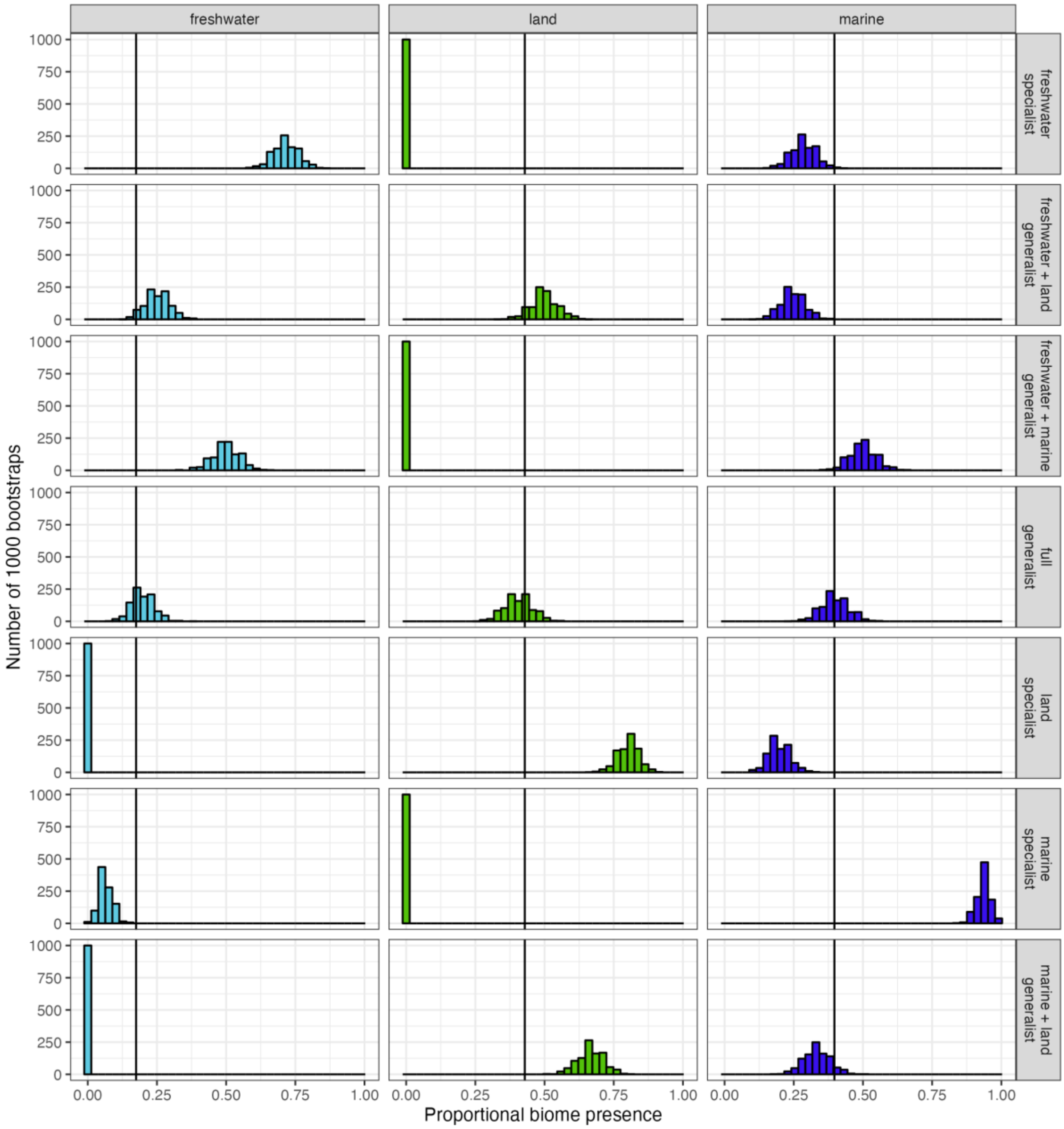

Figure S2. Visualisation of the bootstrap method used for assigning biome preference. Each row represents a single ASV that was assigned into each of the seven biome preferences. Each column shows the proportional presence across all 1000 bootstrapped replicates for each biome. The black lines indicate the proportion of samples that were assigned to each biome. If a lineage is associated with all biomes, we would predict the distributions of proportional presence to overlap the proportions of the samples assigned to each biome. This can be seen in the 4th row. If just 2.5% of the distribution was above the value expected by the number of samples, we classified the ASV as having an affinity with that biome. An example of this can be seen in the 7th row, where only a small proportion of the distribution crossed the proportion of marine-assigned samples, yet the ASV was assigned as a marine + land generalist. This does mean there are some instances where lineages are present in a biome, but are not assigned to have affinity with it. An example of this can be seen in the first row, where the ASV occurs in marine-assigned samples, but below the proportion expected by the proportion of marine-assigned samples, so is classified as a freshwater specialist.

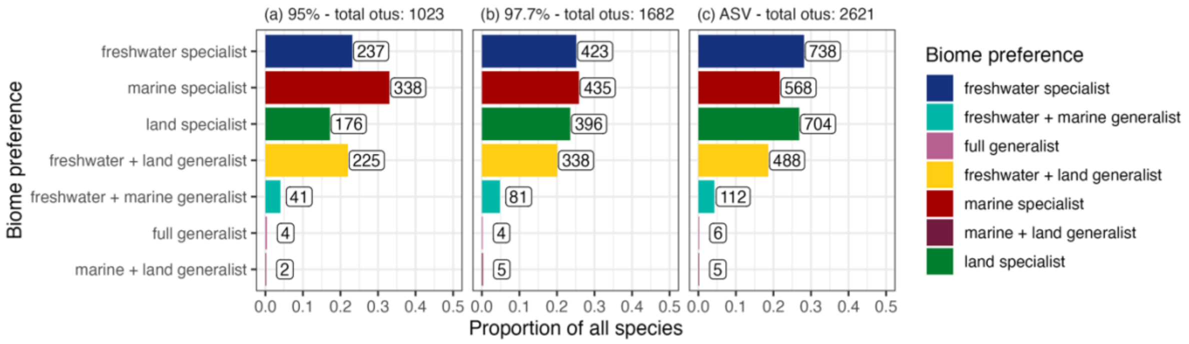

Figure S3. Total diversity and numbers of ASVs in each biome preference at the ASV-level, 97.7% OTU level, and 95% OTU level. To look at the impact of different species delineation definitions on our results, we clustered the data at three levels of phylogenetic similarity (ASV (100%), 97.7% and 95% OTU similarity. We then performed prevalence filtering and assigned biome preference to each sequence variant. Proportions of sequence variants in each biome preference stay stable across levels of phylogenetic similarity: there are very few full generalists or marine + land generalists, and the dataset is mostly biome specialists.

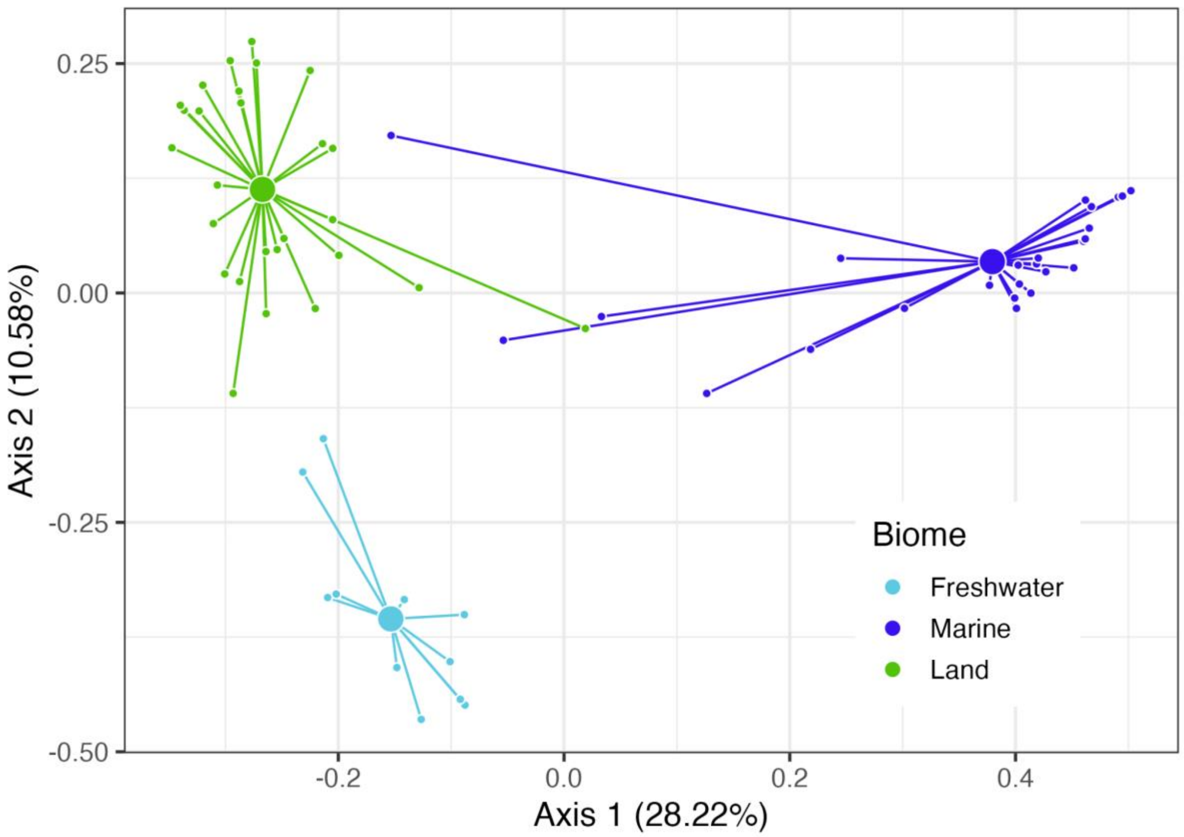

Figure S4. Principal Coordinate (PCoA) plot of samples based on weighted-Unifrac distance of the *Myxococcota* communities based on rpoB sequencing. There is significant clustering of *Myxococcota* communities, indicating that there the community composition is different in the different biomes. Each small point is an individual sample, large points are the positions of centroids of that group of samples, and lines connect individual samples to the group centroids.

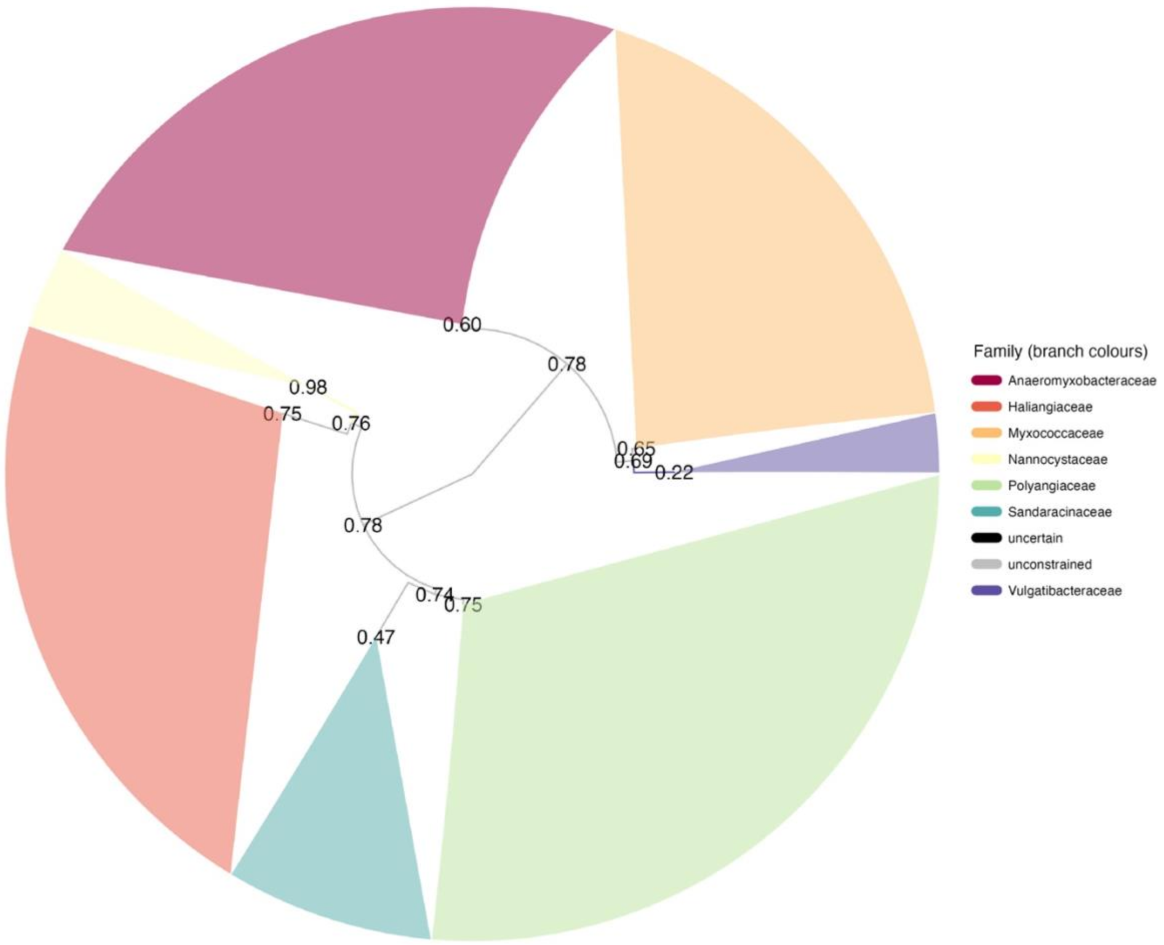

Figure S5. Ultrametric phylogenetic tree of *Myxococcotta* from the *rpoB* sequencing at the ASV level with bootstrapped uncertainty. To make the bootstrapped values of deeper nodes visible, families that were used to constrain the topology of the tree have been collapsed. The bootstrapped tbe values are calculated from bootstrapping done using *raxml-ng*, and the tbe values were calculated. Values closer to 1 indicate more support. The support values for nodes in each family are presented in Figures S6-S12.

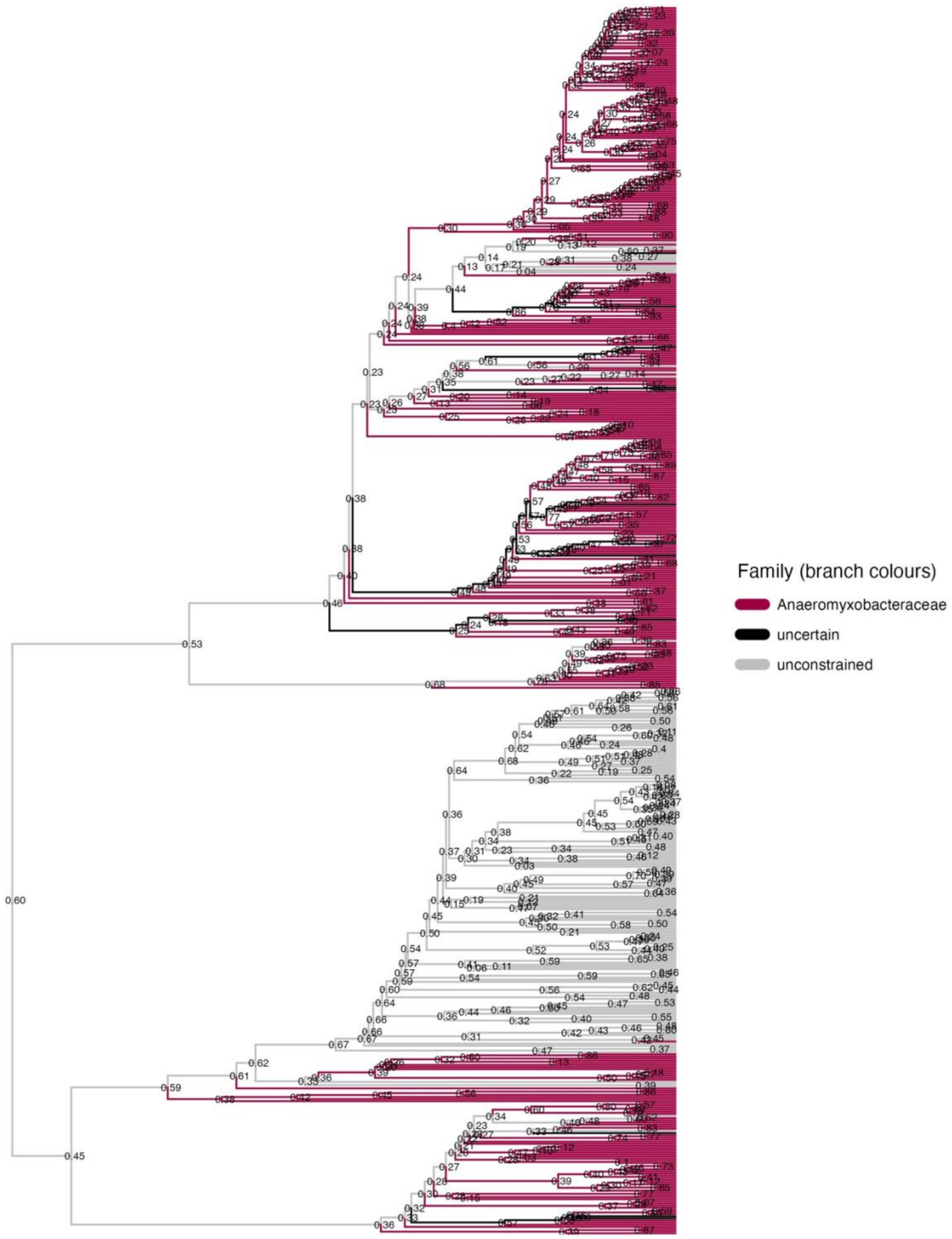

Figure S6. Ultrametric phylogenetic tree of the family *Anaeromyxobacteraceae* from the *rpoB* sequencing at the ASV level with bootstrapped uncertainty. The bootstrapped tbe values are calculated from bootstrapping done using *raxml-ng*, and the tbe values were calculated. Values closer to 1 indicate more support. Branch colours represent different taxonomic assignments; black represents ASVs without a family assignment, and grey represents unconstrained ASVs.

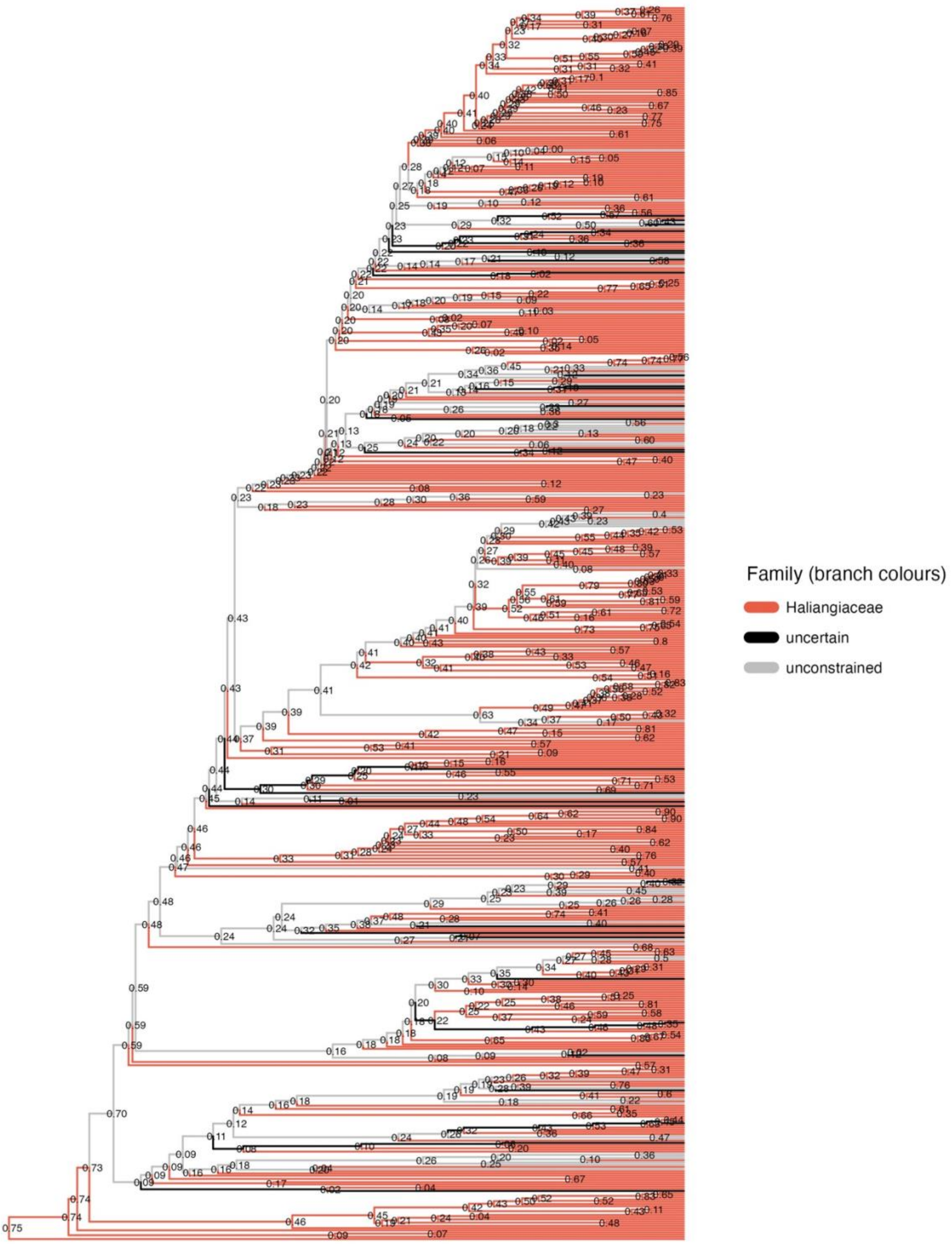

Figure S7. Ultrametric phylogenetic tree of the family *Haliangiaceae* from the *rpoB* sequencing at the ASV level with bootstrapped uncertainty. The bootstrapped tbe values are calculated from bootstrapping done using *raxml-ng*, and the tbe values were calculated. Values closer to 1 indicate more support. Branch colours represent different taxonomic assignments; black represents ASVs without a family assignment, and grey represents unconstrained ASVs.

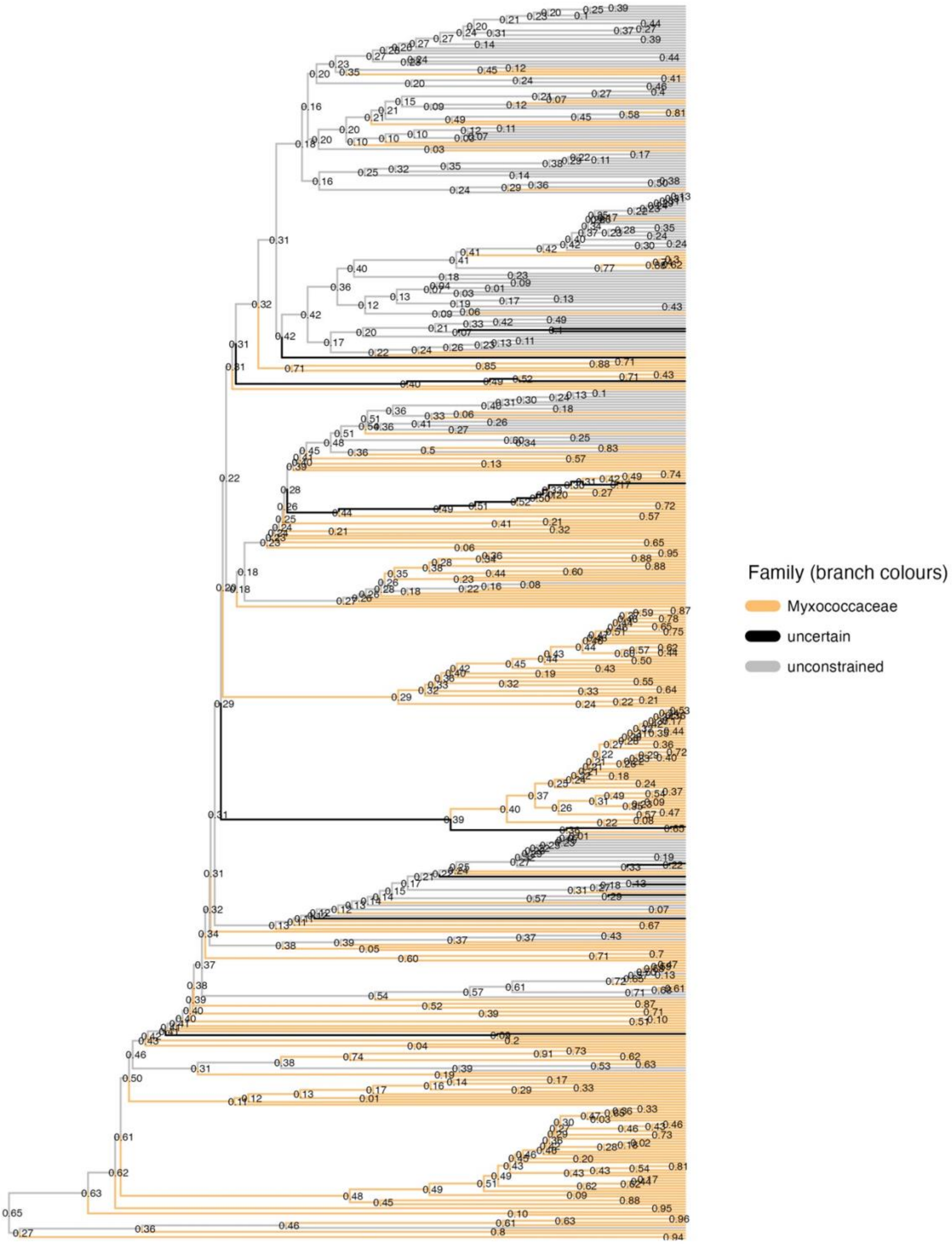

Figure S8. Ultrametric phylogenetic tree of the family *Myxococcaceae* from the *rpoB* sequencing at the ASV level with bootstrapped uncertainty. The bootstrapped tbe values are calculated from bootstrapping done using *raxml-ng*, and the tbe values were calculated. Values closer to 1 indicate more support. Branch colours represent different taxonomic assignments; black represents ASVs without a family assignment, and grey represents unconstrained ASVs.

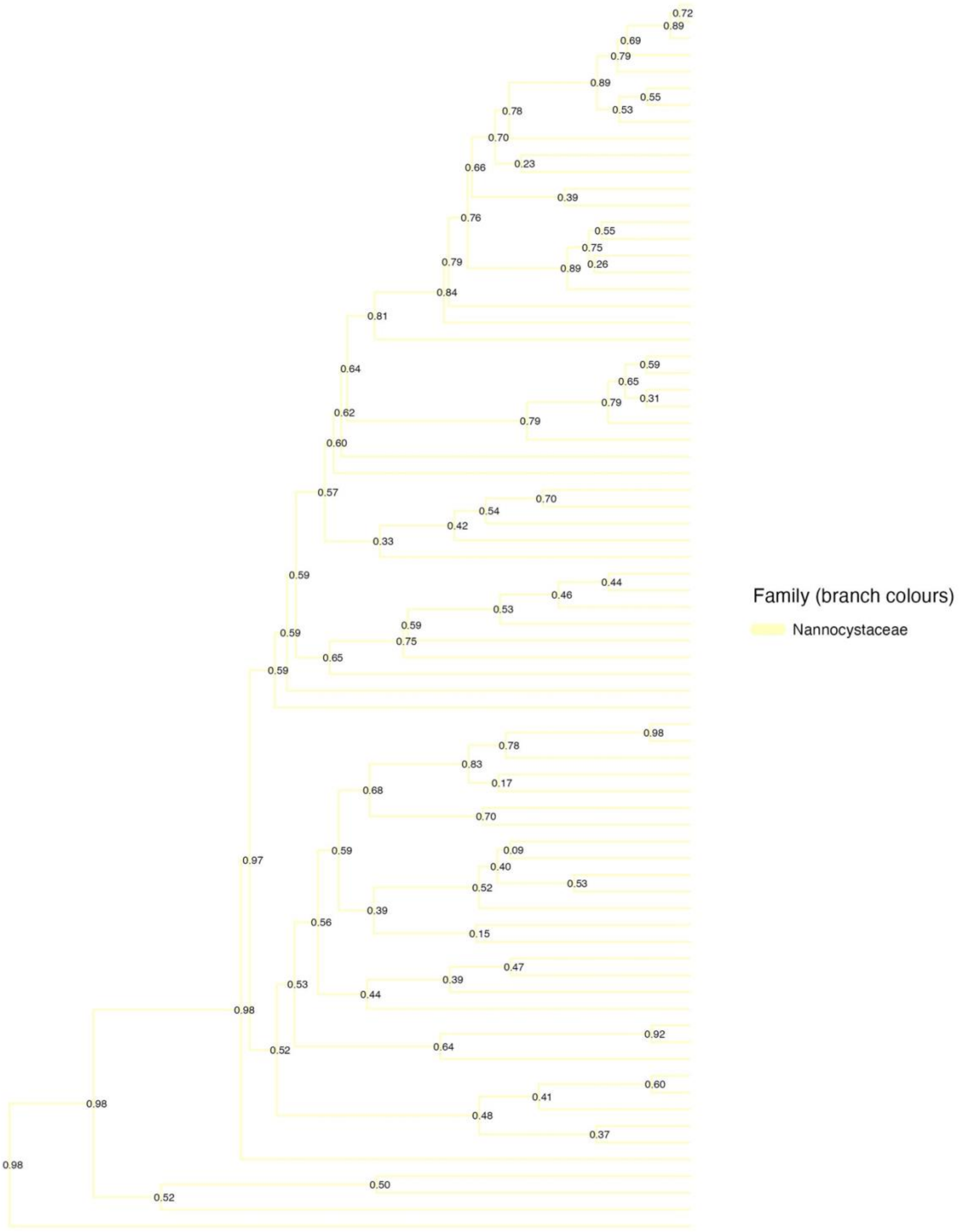

Figure S9. Ultrametric phylogenetic tree of the family *Nannocystaceae* from the *rpoB* sequencing at the ASV level with bootstrapped uncertainty. The bootstrapped tbe values are calculated from bootstrapping done using *raxml-ng*, and the tbe values were calculated. Values closer to 1 indicate more support. Branch colours represent different taxonomic assignments.

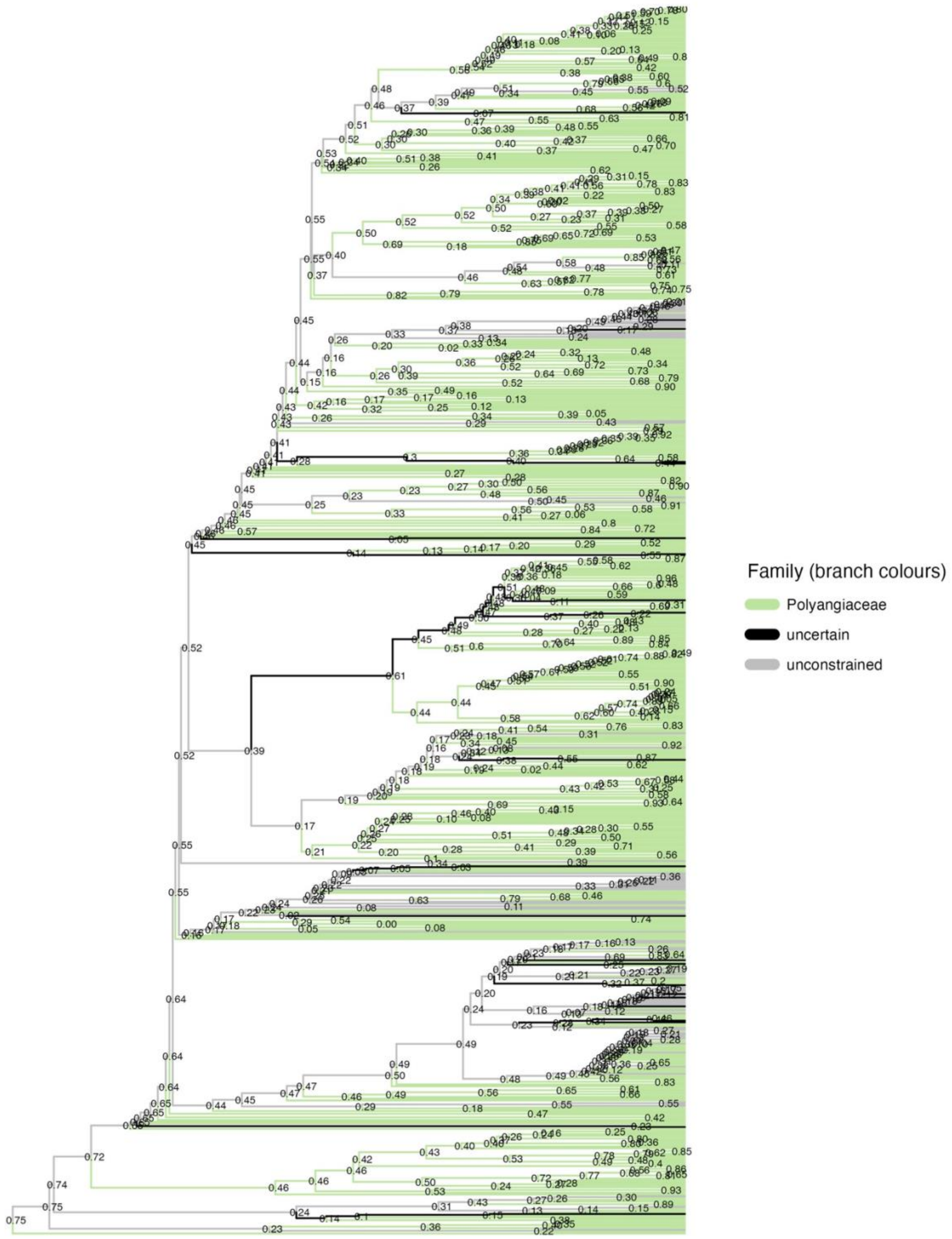

Figure S10. Ultrametric phylogenetic tree of the family *Polyangiaceae* from the *rpoB* sequencing at the ASV level with bootstrapped uncertainty. The bootstrapped tbe values are calculated from bootstrapping done using *raxml-ng*, and the tbe values were calculated. Values closer to 1 indicate more support. Branch colours represent different taxonomic assignments; black represents ASVs without a family assignment, and grey represents unconstrained ASVs.

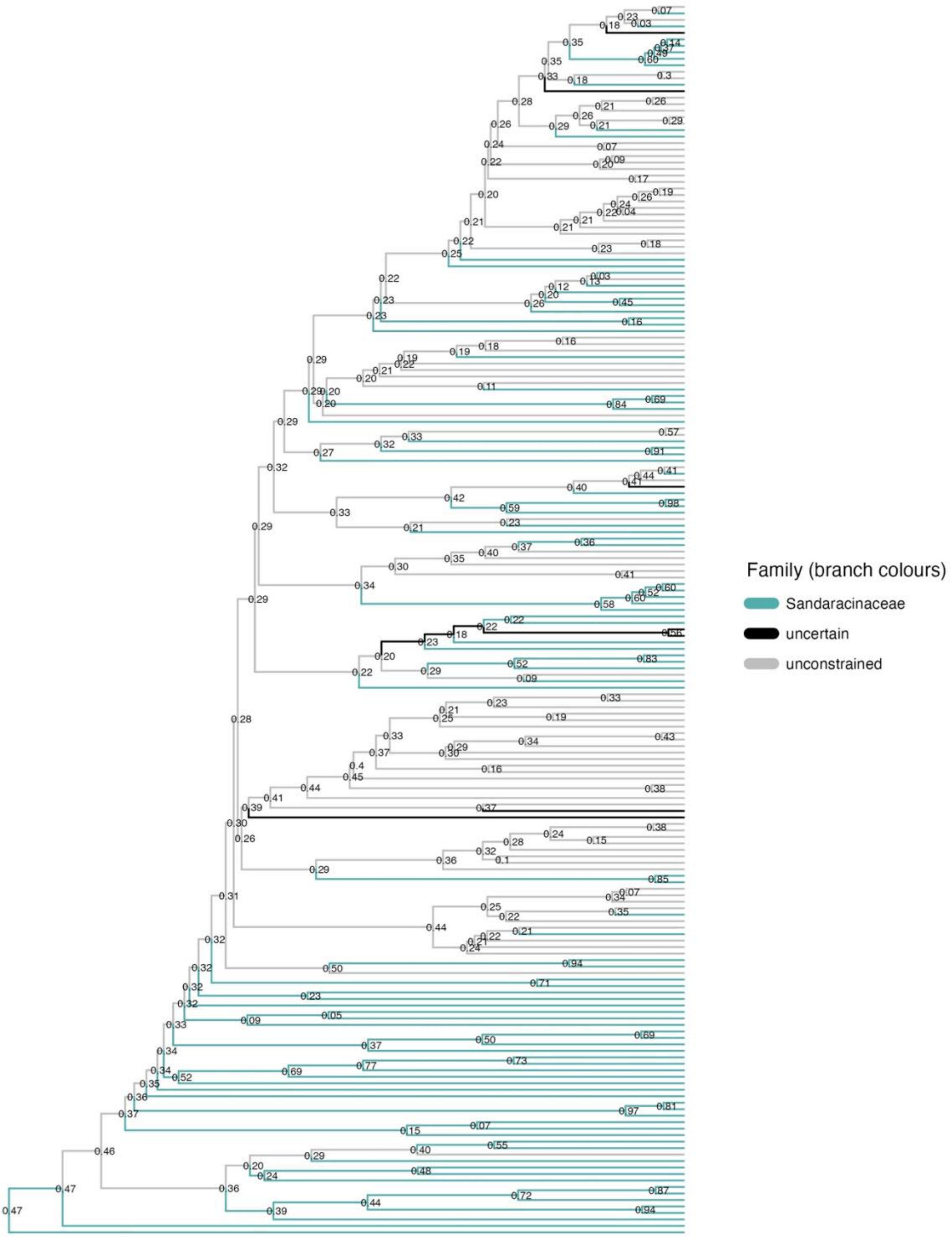

Figure S11. Ultrametric phylogenetic tree of the family *Sandaracinaceae* from the *rpoB* sequencing at the ASV level with bootstrapped uncertainty. The bootstrapped tbe values are calculated from bootstrapping done using *raxml-ng*, and the tbe values were calculated. Values closer to 1 indicate more support. Branch colours represent different taxonomic assignments; black represents ASVs without a family assignment, and grey represents unconstrained ASVs.

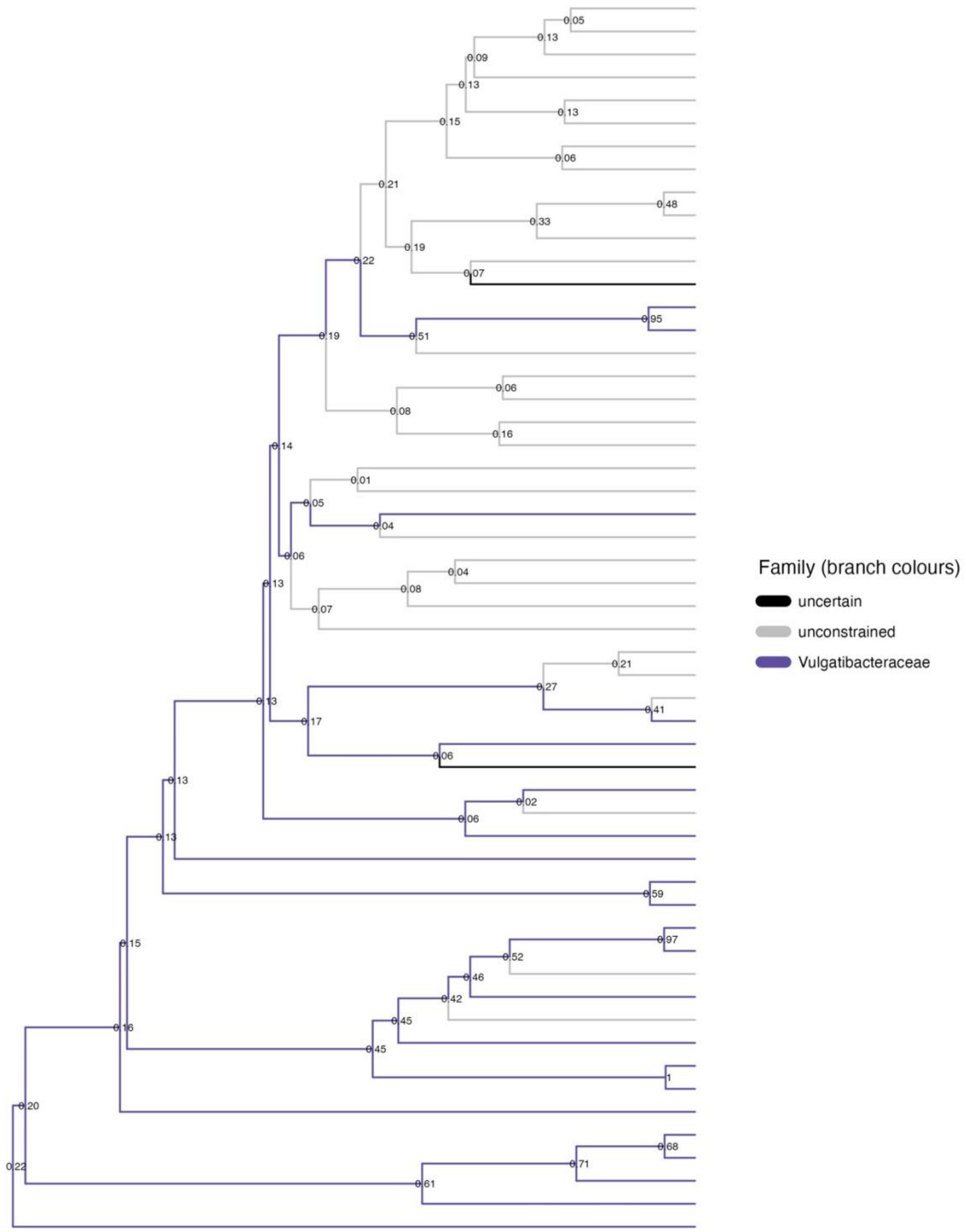

Figure S12. Ultrametric phylogenetic tree of the family *Vulgatibacteraceae* from the *rpoB* sequencing at the ASV level with bootstrapped uncertainty. The bootstrapped tbe values are calculated from bootstrapping done using *raxml-ng*, and the tbe values were calculated. Values closer to 1 indicate more support. Branch colours represent different taxonomic assignments; black represents ASVs without a family assignment, and grey represents unconstrained ASVs.

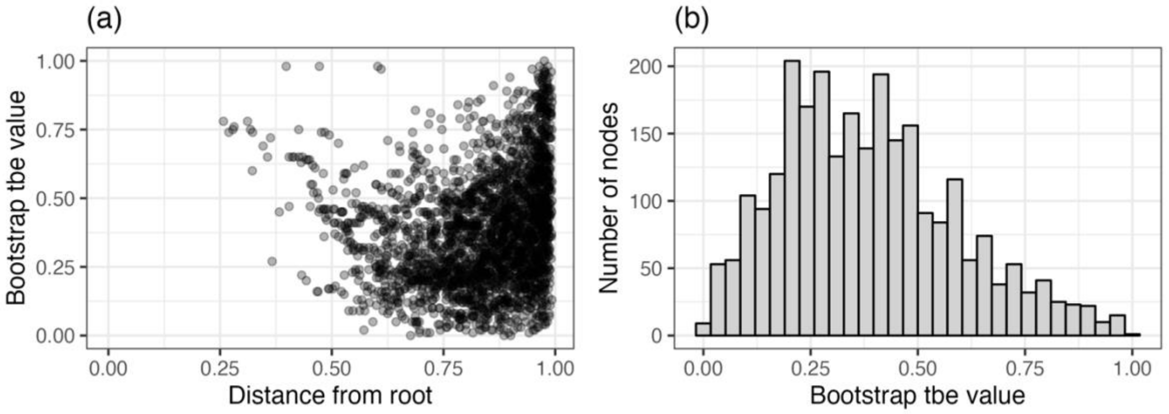

Figure S13. Phylogenetic uncertainty of the Myxococcota ASV phylogeny from the rpoB sequencing. (a) The relationship between distance from the root and the bootstrap tbe value – a measure of phylogenetic uncertainty – at each node of the tree. The deepest nodes have relatively low uncertainty (high tbe values), with values becoming lower further away from the root. We also see very high variation in tbe values close to the present. (b) The distribution of the bootstrap tbe values across the whole tree.

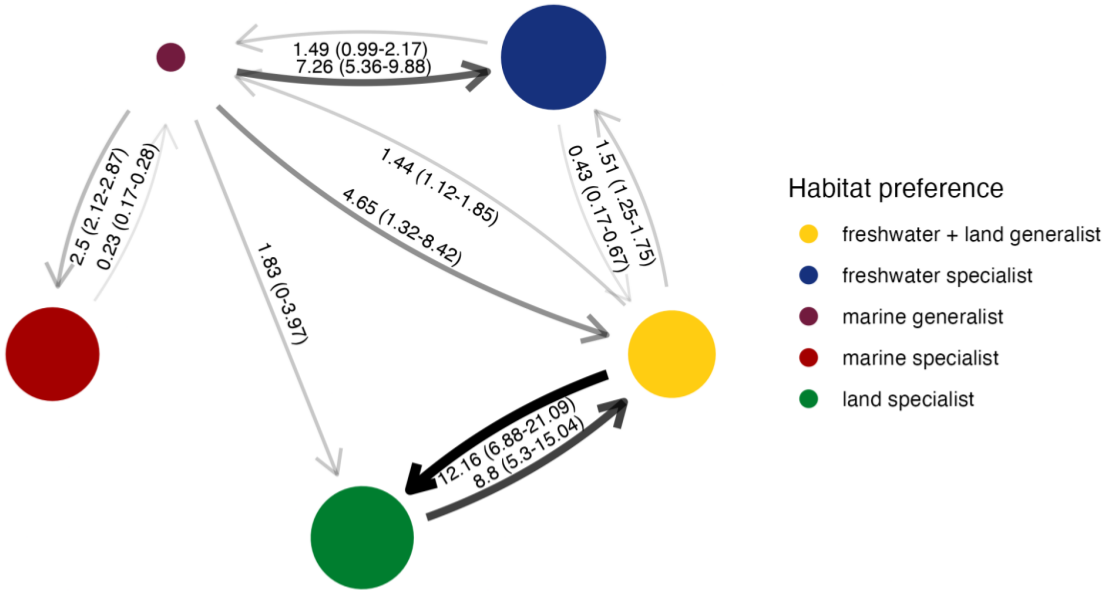

habitat preference	away	into	source sink ratio
marine generalist	5.38 (4.14-8.07)	4.57 (3.16-7.52)	1.19 (1.07-1.34)
freshwater + land generalist	13.42 (3.79-26.59)	13.32 (3.7-26.69)	1.02 (0.94-1.11)
land specialist	11.18 (2.36-24.22)	11.55 (2.76-24.57)	0.93 (0.79-1.04)
freshwater specialist	2.86 (1.63-6.35)	3.08 (2.07-6.39)	0.91 (0.73-1.1)
marine specialist	0.37 (0.19-0.57)	0.69 (0.49-1.02)	0.56 (0.21-0.96)

Figure S14. Bootstrapped (using random sampling) transition rates between biome preferences for the best-supported model of discrete character evolution. The best-supported model was a simplification of the ARD where low transition rates were removed. The table shows the total rates into and away from each biome preference. Bootstrapping was done by randomly sampling 80% of the tips of the tree and re-fitting the best-supported model. Mean estimates are presented, and values in brackets represent 95% confidence intervals. The radius of circles is proportional to the number of ASVs in each biome preference. The size of the arrows is proportional to the transition rate. All values are labelled to two decimal places.

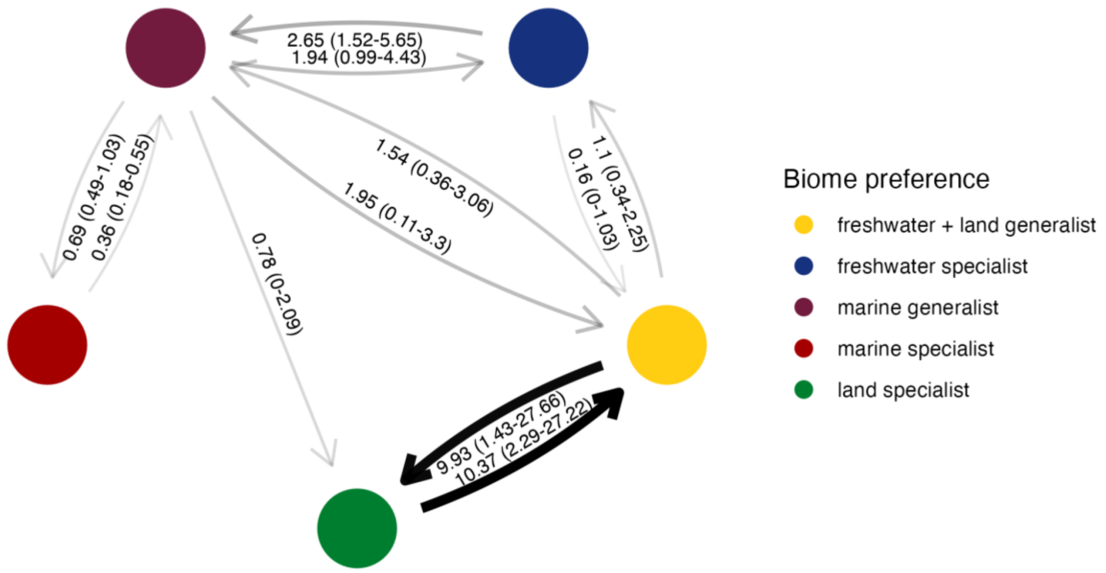

biome preference	away	into	source sink ratio
marine generalist	5.36 (4.19-7.82)	4.55 (3.23-7.23)	1.19 (1.06-1.34)
freshwater + land generalist	12.58 (3.67-30.56)	12.48 (3.59-30.73)	1.02 (0.94-1.13)
land specialist	10.37 (2.29-27.22)	10.71 (2.67-27.66)	0.93 (0.77-1.03)
freshwater specialist	2.81 (1.67-6.53)	3.04 (2.05-6.24)	0.91 (0.75-1.09)
marine specialist	0.36 (0.18-0.55)	0.69 (0.49-1.03)	0.56 (0.19-0.92)

Figure S15. Bootstrapped (using stratified) transition rates between biome preferences for the best-supported model of discrete character evolution. The best-supported model was a simplification of the ARD where low transition rates were removed. The table shows the total rates into and away from each biome preference. Bootstrapping was done by randomly sampling tips within each biome preference such that each had the same number of tips in the tree. Mean estimates are presented, and values in brackets represent 95% confidence intervals. The size of the arrows is proportional to the transition rate. All values are labelled to two decimal places.

Table S2. Model comparison of the Markov models exploring transition rates between biome preferences for the ASV tree.

Model	*d.f.*	log_lik	AIC	AIC weight
Simplified ARD 3	11	-2,674.37	5,370.74	0.53
Simplified ARD 2	12	-2,674.15	5,372.29	0.24
Stepwise	12	-2,674.71	5,373.42	0.14
Simplified ARD 1	13	-2,674.14	5,374.29	0.09
ARD	20	-2,674.14	5,388.29	0.00
Simplified ARD 4	10	-2,710.26	5,440.51	0.00
SYM	10	-2,780.02	5,580.03	0.00
ER	1	-3,256.93	6,515.85	0.00

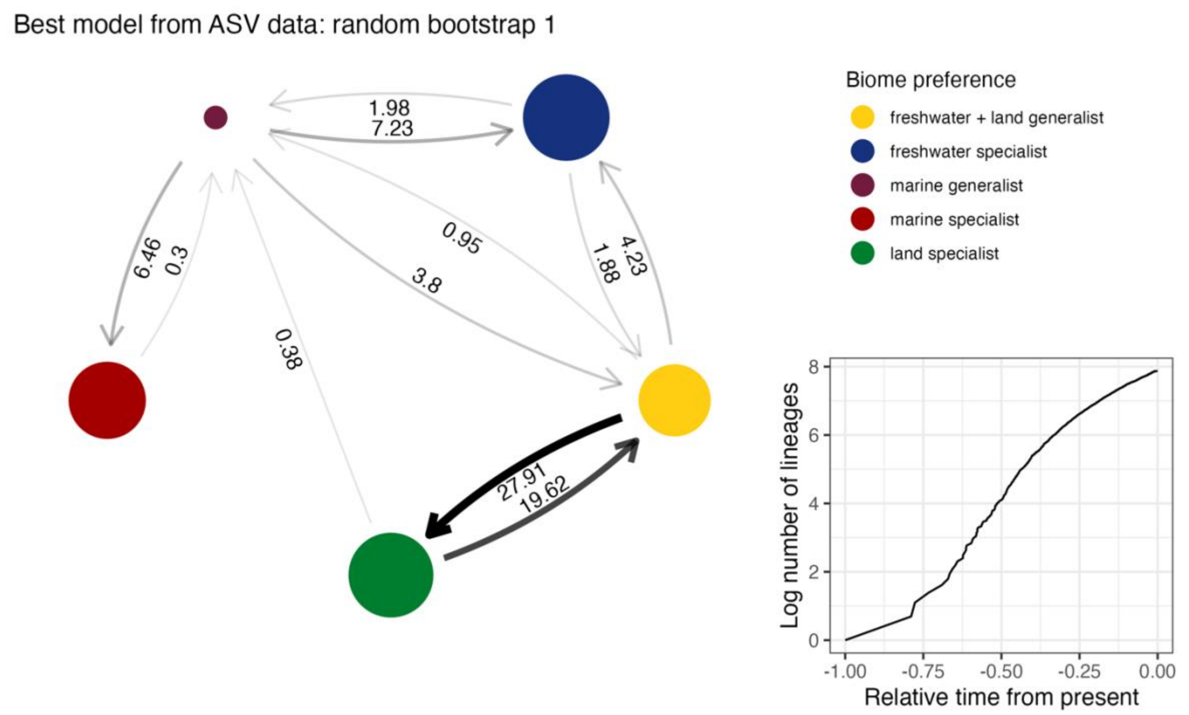

Model	*d.f.*	Log Likelihood	AIC	AIC weight
Simplified ARD 2	11	-2,891.29	5,804.57	0.53
Simplified ARD 1	12	-2,890.96	5,805.92	0.27
Stepwise	12	-2,891.29	5,806.57	0.20
ARD	20	-2,890.96	5,821.92	0.00
Simplified ARD 3	10	-2,911.87	5,843.73	0.00
SYM	10	-3,005.86	6,031.72	0.00
ER	1	-3,418.92	6,839.83	0.00

biome preference	away	into	source sink ratio
marine mud generalist	17.49	3.61	4.85
freshwater + land generalist	33.09	25.30	1.31
land specialist	20.00	27.91	0.72
freshwater specialist	3.86	11.46	0.34
marine mud specialist	0.30	6.46	0.05

Figure S16. Summary of Markov modelling of transition rates between biome preferences for a random ASV bootstrap replicate tree (number 1). The best-supported model (top left) was a simplification of the ARD where low transition rates were removed. The lineage through time plot (middle right) demonstrates that the number of lineages increases linearly through time, with a slight slowdown towards the present. The bottom-left table shows the model comparison of the Markov models exploring transition rates between biome preferences. The bottom-right table shows the total rates into and away from each biome preference calculated from the best-supported model. In the top left figure, the size of the arrows is proportional to the transition rate, and the radius of circles is proportional to the number of ASVs in each biome preference. All values are labelled to two decimal places.

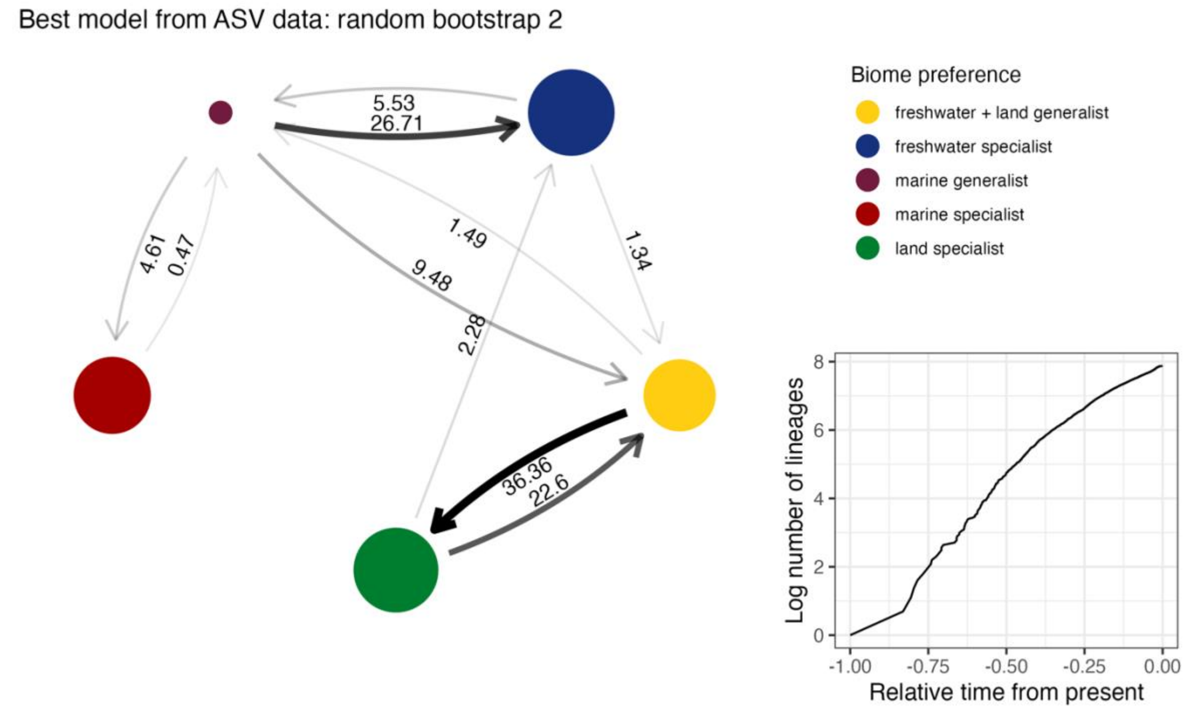

Model	*d.f.*	Log Likelihood	AIC	AIC weight
Simplified ARD 2	10	-2,850.28	5,720.57	0.71
Simplified ARD 1	11	-2,850.28	5,722.56	0.26
Stepwise	12	-2,851.61	5,727.23	0.03
ARD	20	-2,850.28	5,740.56	0.00
Simplified ARD 3	9	-2,883.83	5,785.66	0.00
SYM	10	-2,945.45	5,910.90	0.00
ER	1	-3,415.28	6,832.56	0.00

biome preference	away	into	source sink ratio
marine mud generalist	40.80	7.49	5.44
freshwater + land generalist	37.85	33.42	1.13
land specialist	24.88	36.36	0.68
freshwater specialist	6.88	28.98	0.24
marine mud specialist	0.47	4.61	0.10

Figure S17. Summary of Markov modelling of transition rates between biome preferences for a random ASV bootstrap replicate tree (number 2). The best-supported model (top left) was a simplification of the ARD where low transition rates were removed. The lineage through time plot (middle right) demonstrates that the number of lineages increases linearly through time, with a slight slowdown towards the present. The bottom-left table shows the model comparison of the Markov models exploring transition rates between biome preferences. The bottom-right table shows the total rates into and away from each biome preference calculated from the best-supported model. In the top left figure, the size of the arrows is proportional to the transition rate, and the radius of circles is proportional to the number of ASVs in each biome preference. All values are labelled to two decimal places.

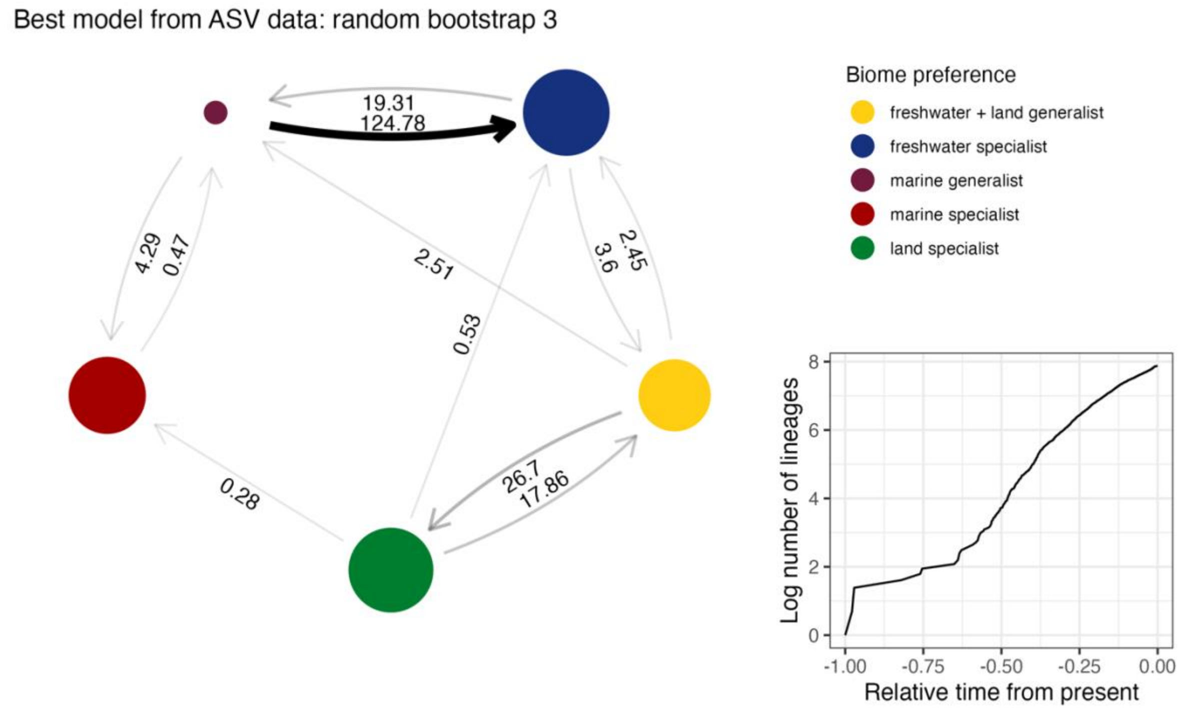

Model	*d.f.*	Log Likelihood	AIC	AIC weight
Simplified ARD 2	11	-2,858.72	5,739.45	0.51
Simplified ARD 1	12	-2,857.92	5,739.85	0.42
Simplified ARD 3	10	-2,861.97	5,743.94	0.05
Stepwise	12	-2,861.43	5,746.86	0.01
ARD	20	-2,857.92	5,755.85	0.00
SYM	10	-2,972.75	5,965.49	0.00
ER	1	-3,424.68	6,851.35	0.00

biome preference	away	into	source sink ratio
marine mud generalist	129.07	22.30	5.79
freshwater + land generalist	31.66	21.46	1.48
land specialist	18.67	26.70	0.70
freshwater specialist	22.91	127.76	0.18
marine mud specialist	0.47	4.57	0.10

Figure S18. Summary of Markov modelling of transition rates between biome preferences for a random ASV bootstrap replicate tree (number 3). The best-supported model (top left) was a simplification of the ARD where low transition rates were removed. The lineage through time plot (middle right) demonstrates that the number of lineages increases linearly through time, with a slight slowdown towards the present. The bottom-left table shows the model comparison of the Markov models exploring transition rates between biome preferences. The bottom-right table shows the total rates into and away from each biome preference calculated from the best-supported model. In the top left figure, the size of the arrows is proportional to the transition rate, and the radius of circles is proportional to the number of ASVs in each biome preference. All values are labelled to two decimal places.

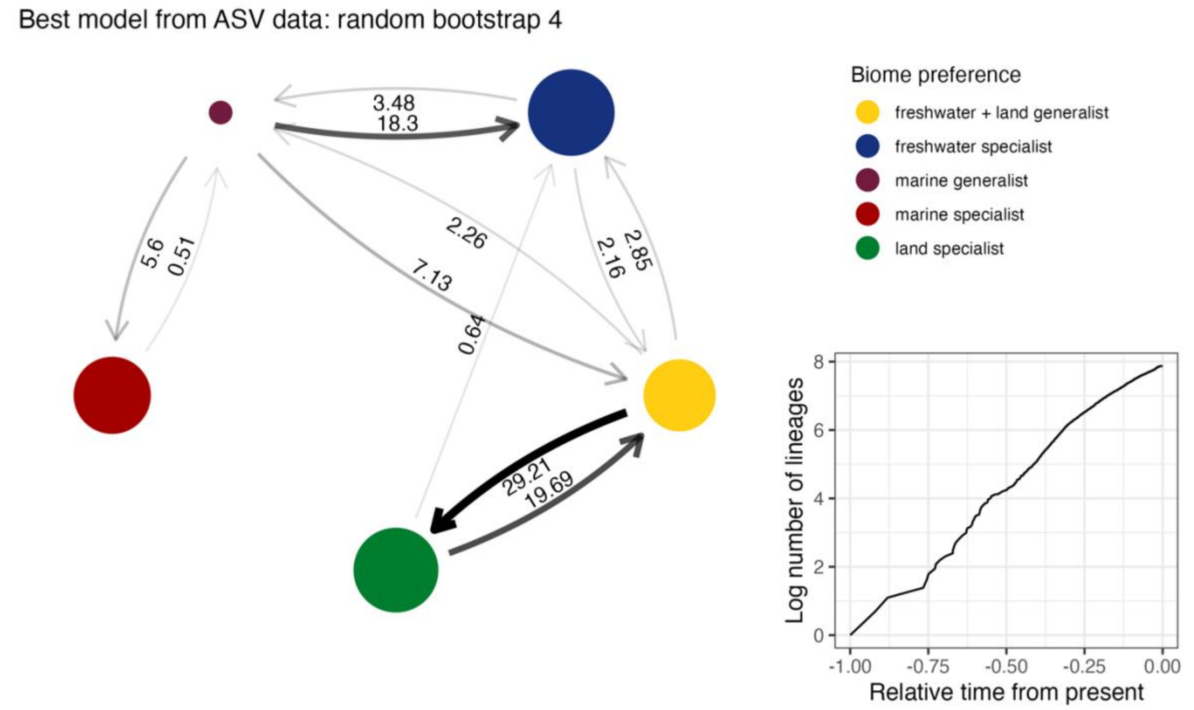

Model	*d.f.*	Log Likelihood	AIC	AIC weight
Simplified ARD 3	11	-2,885.65	5,793.29	0.45
Simplified ARD 2	12	-2,885.05	5,794.10	0.30
Stepwise	12	-2,885.75	5,795.51	0.15
Simplified ARD 1	13	-2,885.05	5,796.10	0.11
ARD	20	-2,885.05	5,810.10	0.00
Simplified ARD 4	10	-2,907.25	5,834.49	0.00
SYM	10	-2,996.42	6,012.84	0.00
ER	1	-3,443.02	6,888.05	0.00

biome preference	away	into	source sink ratio
marine mud generalist	31.02	6.26	4.96
freshwater + land generalist	34.32	28.97	1.18
land specialist	20.33	29.21	0.70
freshwater specialist	5.64	21.79	0.26
marine mud specialist	0.51	5.60	0.09

Figure S19. Summary of Markov modelling of transition rates between biome preferences for a random ASV bootstrap replicate tree (number 4). The best-supported model (top left) was a simplification of the ARD where low transition rates were removed. The lineage through time plot (middle right) demonstrates that the number of lineages increases linearly through time, with a slight slowdown towards the present. The bottom-left table shows the model comparison of the Markov models exploring transition rates between biome preferences. The bottom-right table shows the total rates into and away from each biome preference calculated from the best-supported model. In the top left figure, the size of the arrows is proportional to the transition rate, and the radius of circles is proportional to the number of ASVs in each biome preference. All values are labelled to two decimal places.

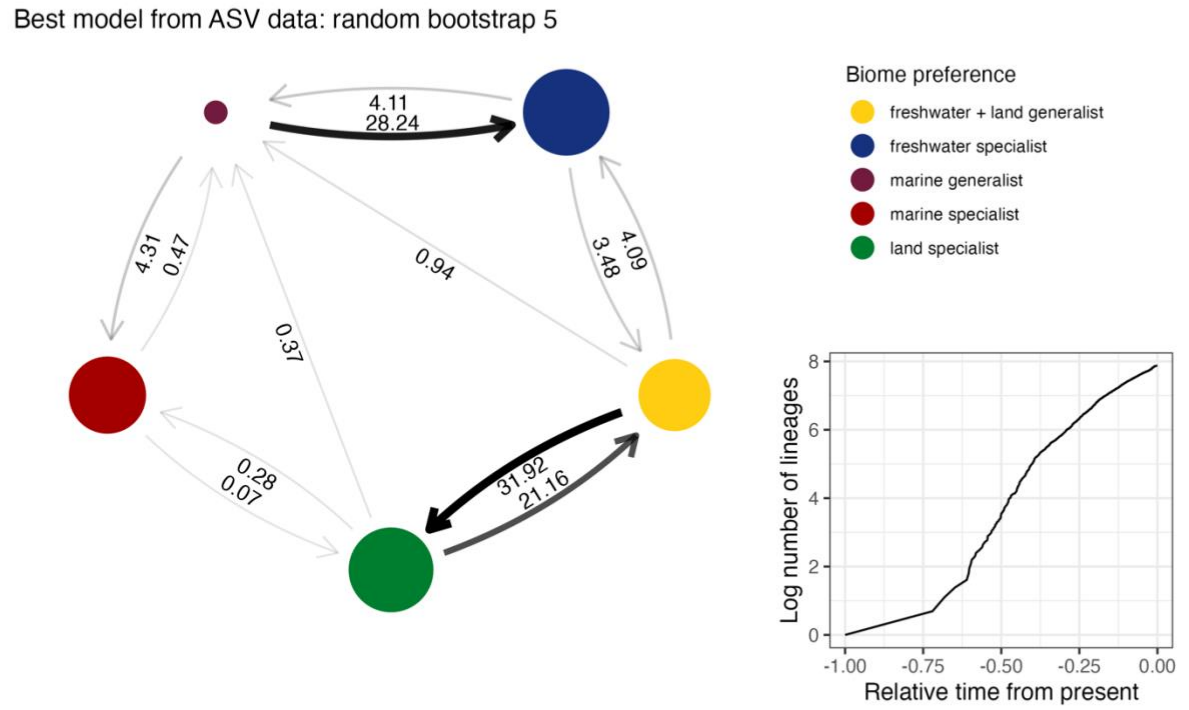

Model	*d.f.*	Log Likelihood	AIC	AIC weight
Simplified ARD 1	12	-2,865.72	5,755.44	0.81
Simplified ARD 2	11	-2,868.35	5,758.70	0.16
Stepwise	12	-2,868.86	5,761.73	0.03
ARD	20	-2,865.72	5,771.44	0.00
SYM	10	-2,969.12	5,958.24	0.00
ER	1	-3,424.44	6,850.89	0.00

biome preference	away	into	source sink ratio
marine mud generalist	32.55	5.89	5.53
freshwater + land generalist	36.96	24.64	1.50
land specialist	21.81	31.99	0.68
freshwater specialist	7.59	32.33	0.23
marine mud specialist	0.54	4.59	0.12

Figure S20. Summary of Markov modelling of transition rates between biome preferences for a random ASV bootstrap replicate tree (number 5). The best-supported model (top left) was a simplification of the ARD where low transition rates were removed. The lineage through time plot (middle right) demonstrates that the number of lineages increases linearly through time, with a slight slowdown towards the present. The bottom-left table shows the model comparison of the Markov models exploring transition rates between biome preferences. The bottom-right table shows the total rates into and away from each biome preference calculated from the best-supported model. In the top left figure, the size of the arrows is proportional to the transition rate, and the radius of circles is proportional to the number of ASVs in each biome preference. All values are labelled to two decimal places.

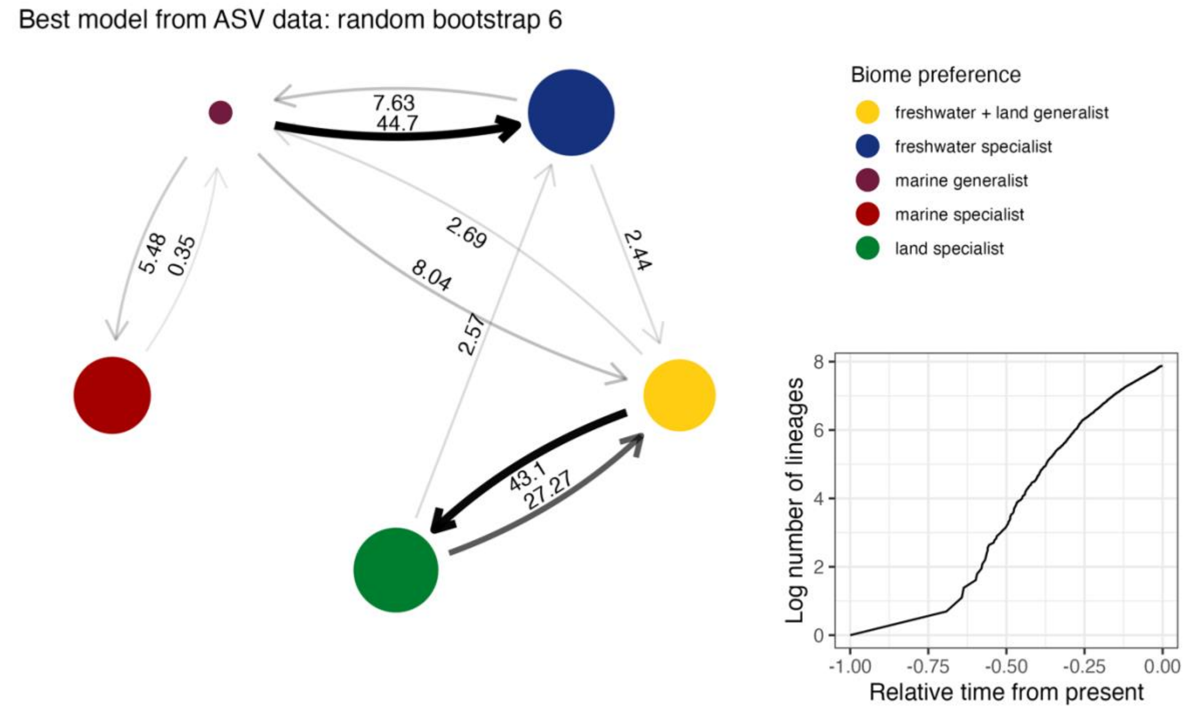

Model	*d.f.*	Log Likelihood	AIC	AIC weight
Simplified ARD 2	10	-2,818.80	5,657.61	0.68
Simplified ARD 1	11	-2,818.76	5,659.52	0.26
Stepwise	12	-2,819.17	5,662.35	0.06
ARD	20	-2,818.76	5,677.52	0.00
Simplified ARD 3	9	-2,847.16	5,712.32	0.00
SYM	10	-2,934.72	5,889.43	0.00
ER	1	-3,423.98	6,849.97	0.00

biome preference	away	into	source sink ratio
marine mud generalist	58.22	10.67	5.46
freshwater + land generalist	45.79	37.75	1.21
land specialist	29.84	43.10	0.69
freshwater specialist	10.07	47.27	0.21
marine mud specialist	0.35	5.48	0.06

Figure S21. Summary of Markov modelling of transition rates between biome preferences for a random ASV bootstrap replicate tree (number 6). The best-supported model (top left) was a simplification of the ARD where low transition rates were removed. The lineage through time plot (middle right) demonstrates that the number of lineages increases linearly through time, with a slight slowdown towards the present. The bottom-left table shows the model comparison of the Markov models exploring transition rates between biome preferences. The bottom-right table shows the total rates into and away from each biome preference calculated from the best-supported model. In the top left figure, the size of the arrows is proportional to the transition rate, and the radius of circles is proportional to the number of ASVs in each biome preference. All values are labelled to two decimal places.

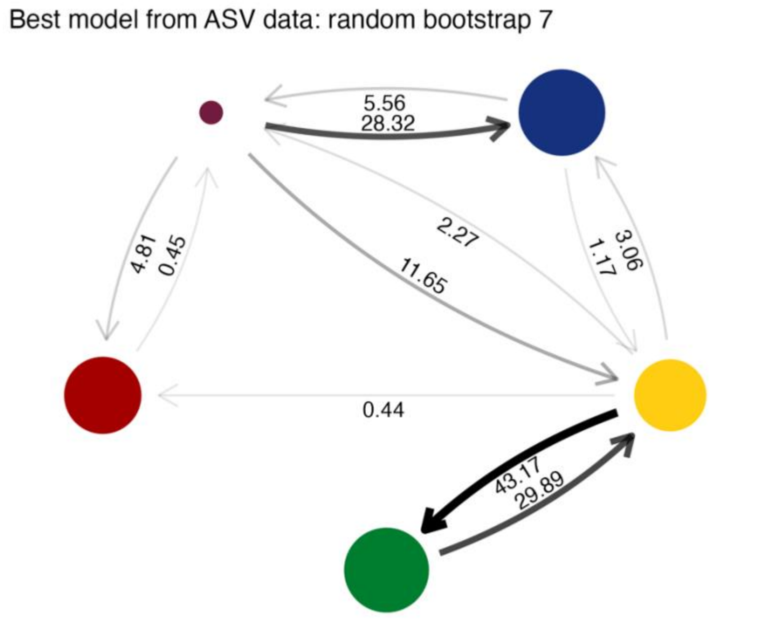

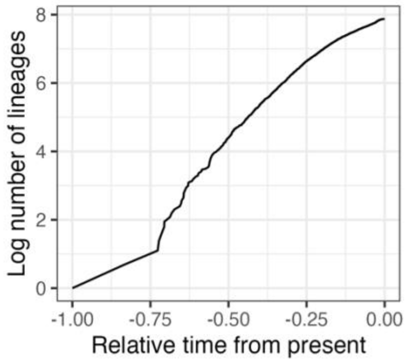

Model	d.f.	Log Likelihood	AIC	AIC weight
Simplified ARD 2	11	-2,963.99	5,949.98	0.58
Simplified ARD 1	12	-2,963.65	5,951.31	0.30
Stepwise	12	-2,964.80	5,953.59	0.10
Simplified ARD 3	11	-2,967.02	5,956.04	0.03
ARD	20	-2,963.65	5,967.31	0.00
SYM	10	-3,068.98	6,157.96	0.00
ER	1	-3,486.79	6,975.57	0.00

biome preference	away	into	source sink ratio
marine mud generalist	44.79	8.27	5.41
freshwater + land generalist	48.93	42.72	1.15
land specialist	29.89	43.17	0.69
freshwater specialist	6.73	31.38	0.21
marine mud specialist	0.45	5.25	0.09

Figure S22. Summary of Markov modelling of transition rates between biome preferences for a random ASV bootstrap replicate tree (number 7). The best-supported model (top left) was a simplification of the ARD where low transition rates were removed. The lineage through time plot (middle right) demonstrates that the number of lineages increases linearly through time, with a slight slowdown towards the present. The bottom-left table shows the model comparison of the Markov models exploring transition rates between biome preferences. The bottom-right table shows the total rates into and away from each biome preference calculated from the best-supported model. In the top left figure, the size of the arrows is proportional to the transition rate, and the radius of circles is proportional to the number of ASVs in each biome preference. All values are labelled to two decimal places.

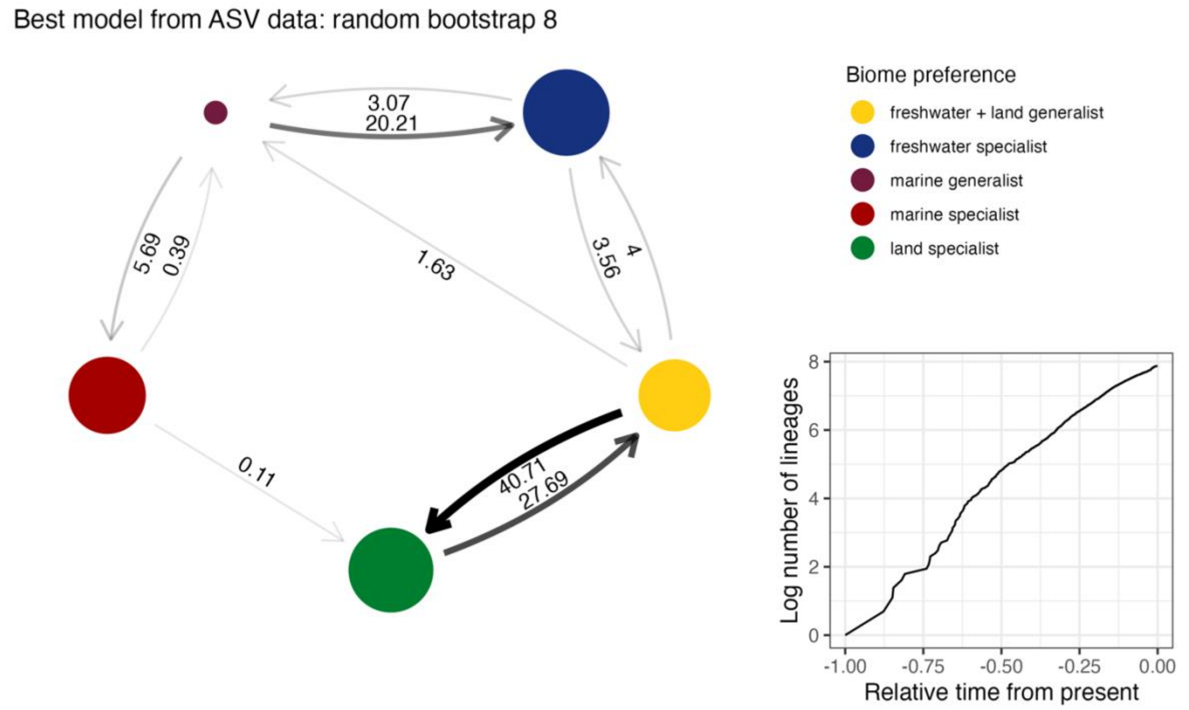

Model	*d.f.*	Log Likelihood	AIC	AIC weight
Simplified ARD 2	10	-2,915.78	5,851.57	0.62
Simplified ARD 1	11	-2,915.44	5,852.88	0.32
Simplified ARD 3	9	-2,919.48	5,856.97	0.04
Stepwise	12	-2,917.42	5,858.85	0.02
ARD	20	-2,915.44	5,870.88	0.00
SYM	10	-3,017.92	6,055.84	0.00
ER	1	-3,482.18	6,966.36	0.00

biome preference	away	into	source sink ratio
marine mud generalist	25.90	5.09	5.09
freshwater + land generalist	46.34	31.25	1.48
land specialist	27.69	40.81	0.68
freshwater specialist	6.63	24.21	0.27
marine mud specialist	0.50	5.69	0.09

Figure S23. Summary of Markov modelling of transition rates between biome preferences for a random ASV bootstrap replicate tree (number 8). The best-supported model (top left) was a simplification of the ARD where low transition rates were removed. The lineage through time plot (middle right) demonstrates that the number of lineages increases linearly through time, with a slight slowdown towards the present. The bottom-left table shows the model comparison of the Markov models exploring transition rates between biome preferences. The bottom-right table shows the total rates into and away from each biome preference calculated from the best-supported model. In the top left figure, the size of the arrows is proportional to the transition rate, and the radius of circles is proportional to the number of ASVs in each biome preference. All values are labelled to two decimal places.

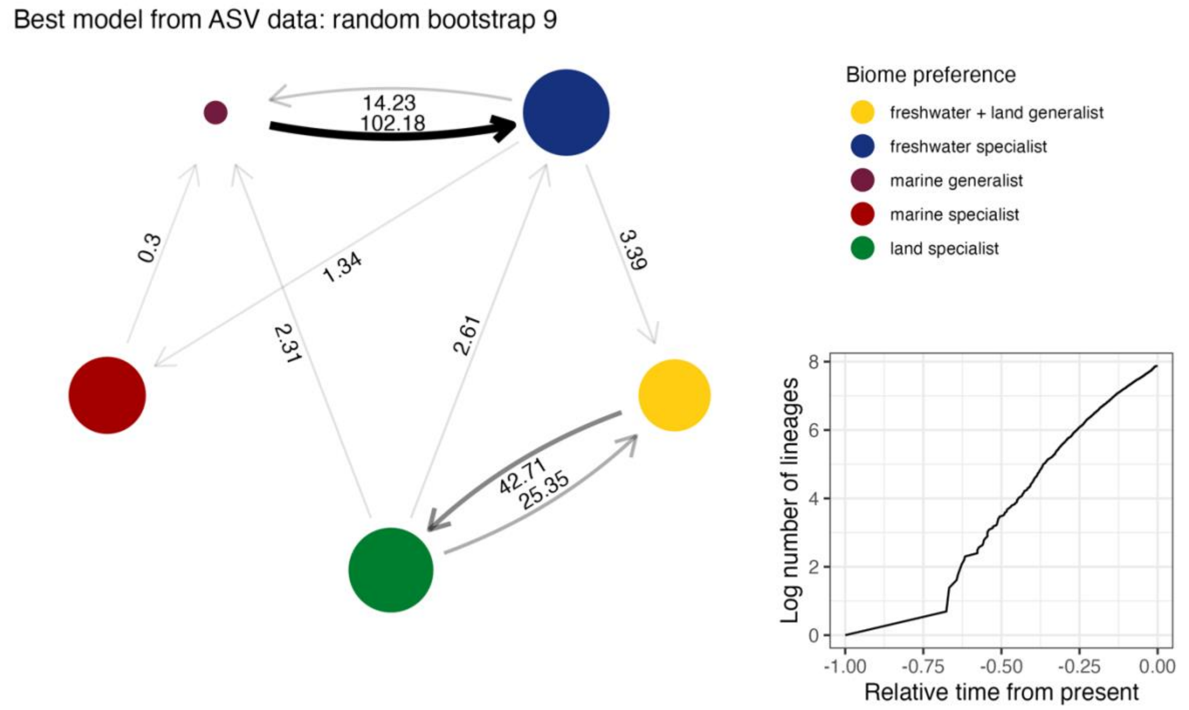

Model	*d.f.*	Log Likelihood	AIC	AIC weight
Simplified ARD 2	9	-2,845.06	5,708.13	0.71
Simplified ARD 1	10	-2,844.94	5,709.88	0.29
Stepwise	12	-2,848.01	5,720.01	0.00
ARD	20	-2,844.94	5,729.88	0.00
Simplified ARD 3	8	-2,912.36	5,840.72	0.00
SYM	10	-2,969.17	5,958.33	0.00
ER	1	-3,448.51	6,899.01	0.00

biome preference	away	into	source sink ratio
marine mud generalist	102.18	16.83	6.07
freshwater + land generalist	42.71	28.74	1.49
land specialist	30.26	42.71	0.71
marine mud specialist	0.30	1.34	0.22
freshwater specialist	18.96	104.79	0.18

Figure S24. Summary of Markov modelling of transition rates between biome preferences for a random ASV bootstrap replicate tree (number 9). The best-supported model (top left) was a simplification of the ARD where low transition rates were removed. The lineage through time plot (middle right) demonstrates that the number of lineages increases linearly through time, with a slight slowdown towards the present. The bottom-left table shows the model comparison of the Markov models exploring transition rates between biome preferences. The bottom-right table shows the total rates into and away from each biome preference calculated from the best-supported model. In the top left figure, the size of the arrows is proportional to the transition rate, and the radius of circles is proportional to the number of ASVs in each biome preference. All values are labelled to two decimal places.

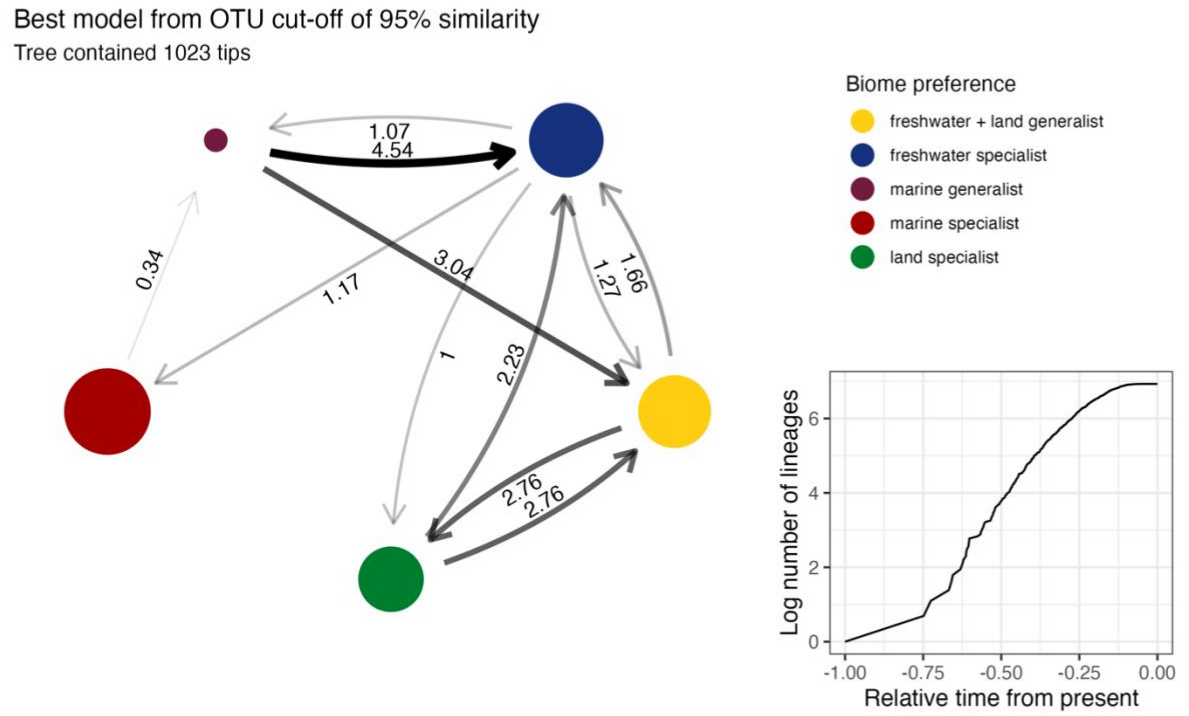

Model	d.f.	Log Likelihood	AIC	AIC weight
Simplified ARD 6	11	-1,183.53	2,389.07	0.43
Simplified ARD 4	13	-1,182.34	2,390.69	0.19
Simplified ARD 5	12	-1,183.47	2,390.93	0.17
Simplified ARD 3	14	-1,182.08	2,392.15	0.09
Simplified ARD 2	15	-1,181.42	2,392.84	0.07
Stepwise	12	-1,184.96	2,393.91	0.04
Simplified ARD 1	16	-1,181.75	2,395.51	0.02
ARD	20	-1,181.75	2,403.51	0.00
SYM	10	-1,207.48	2,434.95	0.00
Simplified ARD 7	10	-1,239.90	2,499.80	0.00
ER	1	-1,350.21	2,702.42	0.00

biome preference	away	into	source sink ratio
marine mud generalist	7.58	1.41	5.37
land specialist	5.00	3.76	1.33
freshwater + land generalist	4.42	7.08	0.62
freshwater specialist	4.51	8.43	0.54
marine mud specialist	0.34	1.17	0.29

Figure S25. Summary of Markov modelling of transition rates between biome preferences for the 95% OTU tree. The best-supported model (top left) was a simplification of the ARD where low transition rates were removed. The lineage through time plot (middle right) demonstrates that the number of lineages increases linearly through time, but with a slowdown of lineages in the in the present. The bottom-left table shows the model comparison of the Markov models exploring transition rates between biome preferences. The bottom-right table shows the total rates into and away from each biome preference calculated from the best-supported model. In the top left figure, the size of the arrows is proportional to the transition rate, and the radius of circles is proportional to the number of ASVs in each biome preference. All values are labelled to two decimal places.

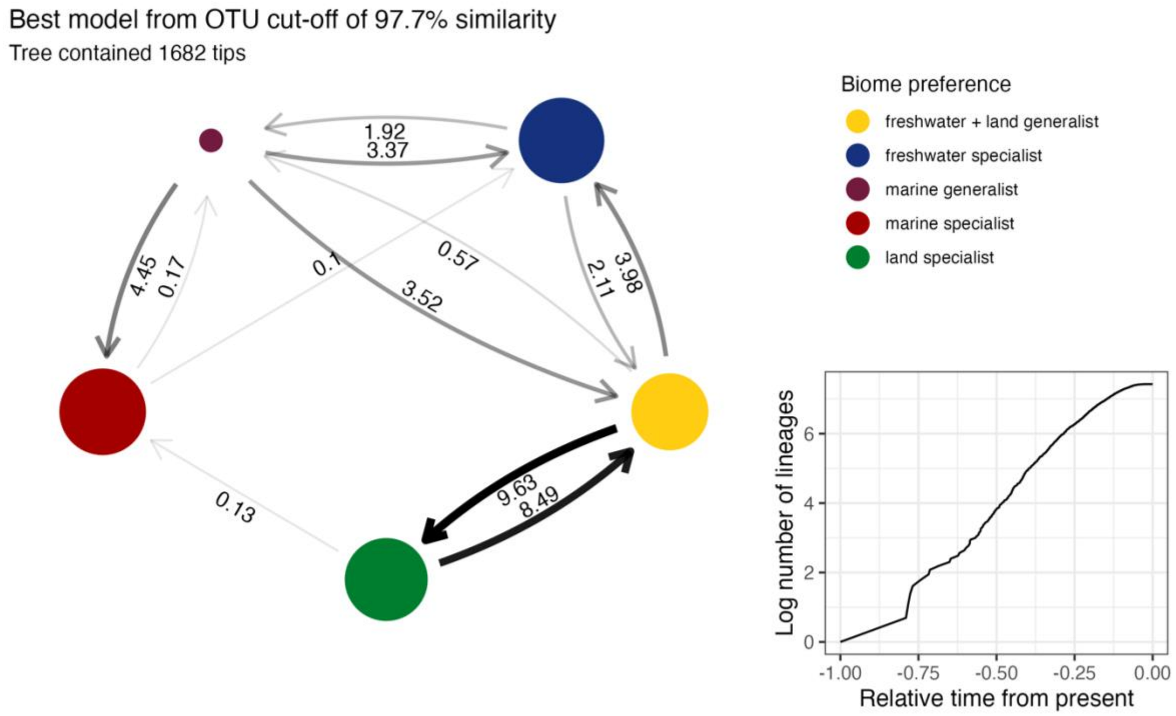

Model	d.f.	log_lik	AIC	AIC weight
Simplified ARD 3	12	-1,901.95	3,827.89	0.30
Simplified ARD 4	11	-1,902.96	3,827.91	0.29
Simplified ARD 5	10	-1,904.27	3,828.54	0.22
Simplified ARD 2	13	-1,901.95	3,829.89	0.11
Simplified ARD 1	14	-1,901.81	3,831.62	0.05
Stepwise	12	-1,904.07	3,832.13	0.04
ARD	20	-1,901.81	3,843.62	0.00
SYM	10	-1,951.90	3,923.80	0.00
ER	1	-2,231.01	4,464.02	0.00

biome preference	away	into	source sink ratio
marine mud generalist	11.34	2.67	4.26
freshwater + land generalist	14.18	14.12	1.00
land specialist	8.62	9.63	0.89
freshwater specialist	4.04	7.45	0.54
marine mud specialist	0.27	4.58	0.06

Figure S26. Summary of Markov modelling of transition rates between biome preferences for the 97.7% OTU tree. The best-supported model (top left) was a simplification of the ARD where low transition rates were removed. The lineage through time plot (middle right) demonstrates that the number of lineages increases linearly through time, but with a slowdown of lineages in the in the present. The bottom-left table shows the model comparison of the Markov models exploring transition rates between biome preferences. The bottom-right table shows the total rates into and away from each biome preference calculated from the best-supported model. In the top left figure, the size of the arrows is proportional to the transition rate, and the radius of circles is proportional to the number of ASVs in each biome preference. All values are labelled to two decimal places.

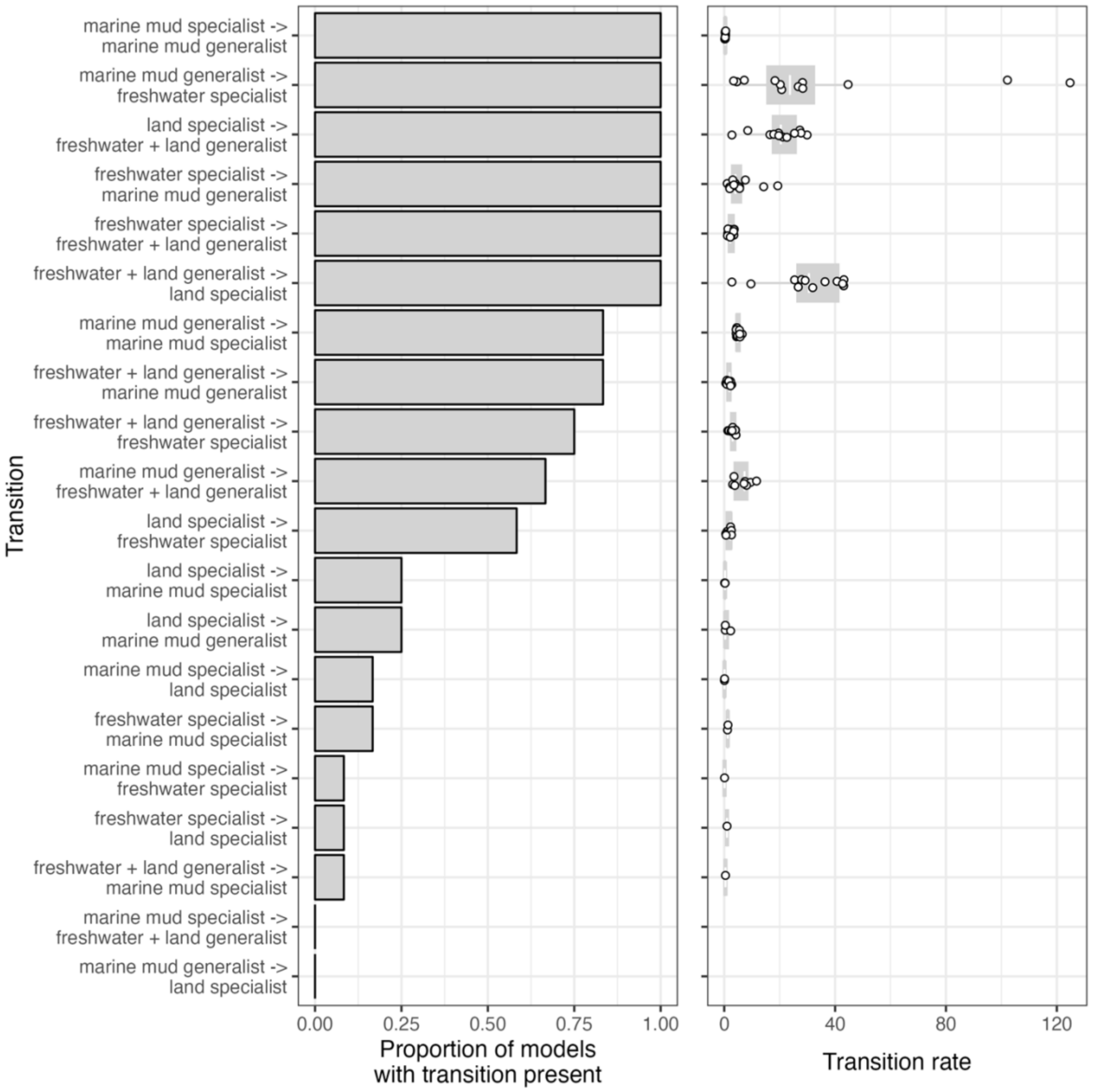

Figure S27. Comparison of transition rates between biome preferences for the ASV tree, 9 ASV bootstrap replicate trees, the 95% OTU tree, and the 97.7% OTU tree. The left-side plot demonstrates the prevalence of each transition in the best Markov model in each tree. The right-side plot shows the rates of each transition in the best Markov model in each tree. In the right-side plot, points represent the transition rate from the best Markov model from a tree, transitions that are not supported are removed before plotting.

Table S3. Comparison of transition rates between biome preferences for the best ASV tree, 9 ASV bootstrap replicate trees, the 95% OTU tree, and the 97.7% OTU tree. All transitions are present, a dash represents where the transition is not present in the best Markov model for that tree, values are round to two decimal places.

from	to	ASV tree	ASV bootstrap 1	ASV bootstrap 2	ASV bootstrap 3	ASV bootstrap 4	ASV bootstrap 5	ASV bootstrap 6	ASV bootstrap 7	ASV bootstrap 8	ASV bootstrap 9	OTU 95% tree	OTU 97.7% tree
freshwater + land generalist	freshwater specialist	1.27	4.23	-	2.45	2.85	4.09	-	3.06	4	-	1.66	3.98
freshwater + land generalist	marine mud generalist	2.14	0.95	1.49	2.51	2.26	0.94	2.69	2.27	1.63	-	-	0.57
freshwater + land generalist	marine mud specialist	-	-	-	-	-	-	-	0.44	-	-	-	-
freshwater + land generalist	land specialist	25.38	27.91	36.36	26.7	29.21	31.92	43.1	43.17	40.71	42.71	2.76	9.63
freshwater specialist	freshwater + land generalist	1.72	1.88	1.34	3.6	2.16	3.48	2.44	1.17	3.56	3.39	1.27	2.11
freshwater specialist	marine mud generalist	3.94	1.98	5.53	19.31	3.48	4.11	7.63	5.56	3.07	14.23	1.07	1.92
freshwater specialist	marine mud specialist	-	-	-	-	-	-	-	-	-	1.34	1.17	-
freshwater specialist	land specialist	-	-	-	-	-	-	-	-	-	-	1	-
marine mud generalist	freshwater + land generalist	7.48	3.8	9.48	-	7.13	-	8.04	11.65	-	-	3.04	3.52
marine mud generalist	freshwater specialist	20.72	7.23	26.71	124.78	18.3	28.24	44.7	28.32	20.21	102.18	4.54	3.37
marine mud generalist	marine mud specialist	4.5	6.46	4.61	4.29	5.6	4.31	5.48	4.81	5.69	-	-	4.45
marine mud generalist	land specialist	-	-	-	-	-	-	-	-	-	-	-	-
marine mud specialist	freshwater + land generalist	-	-	-	-	-	-	-	-	-	-	-	-
marine mud specialist	freshwater specialist	-	-	-	-	-	-	-	-	-	-	-	0.1
marine mud specialist	marine mud generalist	0.29	0.3	0.47	0.47	0.51	0.47	0.35	0.45	0.39	0.3	0.34	0.17
marine mud specialist	land specialist	-	-	-	-	-	0.07	-	-	0.11	-	-	-
land specialist	freshwater + land generalist	16.5	19.62	22.6	17.86	19.69	21.16	27.27	29.89	27.69	25.35	2.76	8.49
land specialist	freshwater specialist	1.03	-	2.28	0.53	0.64	-	2.57	-	-	2.61	2.23	-
land specialist	marine mud generalist	-	0.38	-	-	-	0.37	-	-	-	2.31	-	-
land specialist	marine mud specialist	-	-	-	0.28	-	0.28	-	-	-	-	-	0.13

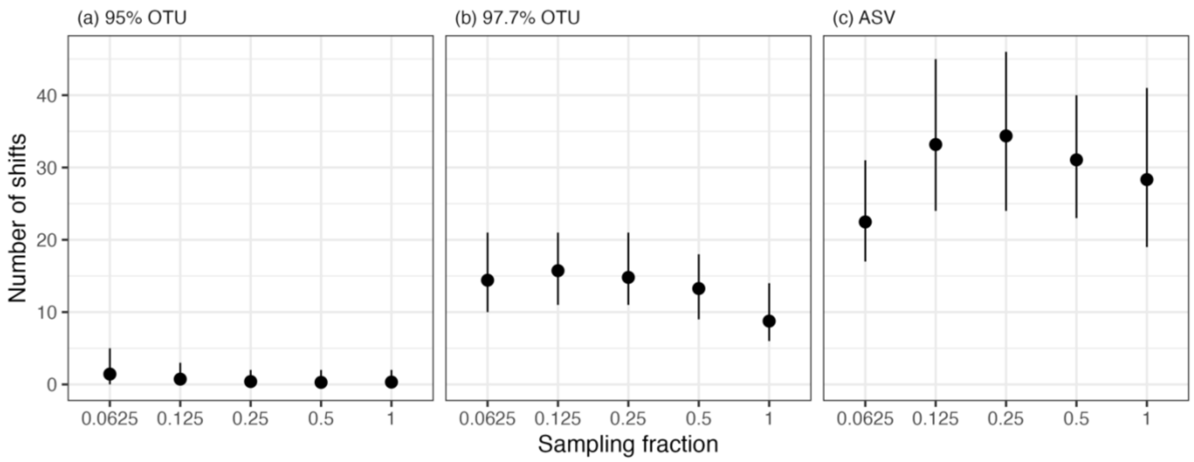

Figure S28. Number of rate shifts in diversification rate identified by BAMM at different sampling fractions on (a) the 95% OTU tree (b) the 97.7% OTU tree and (c) the ASV tree. The number of rate shifts identified decreases sequences are clustered at lower levels of phylogenetic similarity. There is little effect on sampling fraction on the number of rate shifts identified by BAMM. Points are the mean estimate of the number of rate shifts inferred, lines represent 95% credible intervals.

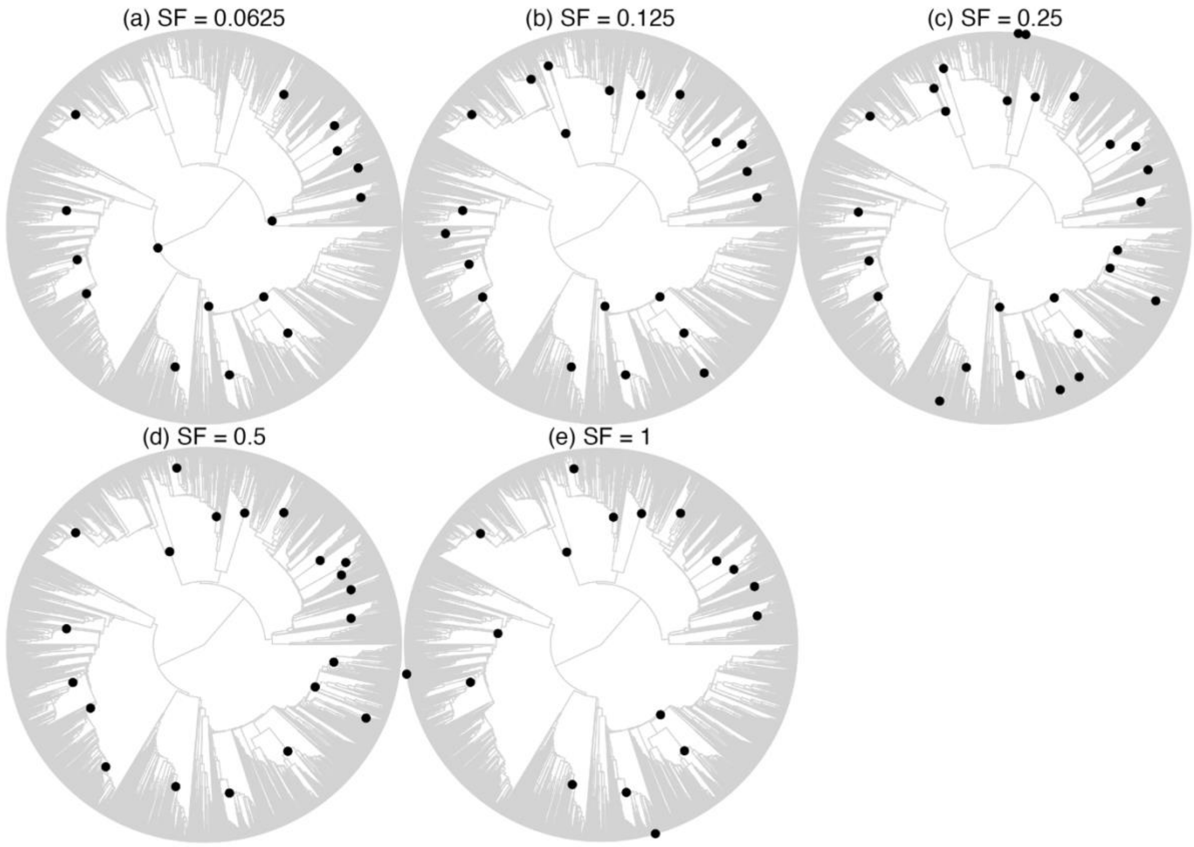

Figure S29. Position of nodes where core rate shifts have been inferred by BAMM in the ASV *Myxococcota* phylogeny. The different panels show the core rate shifts identified by the shift configuration with the highest marginal probability at different sampling fractions (SF). There is some similarity between sampling fractions.

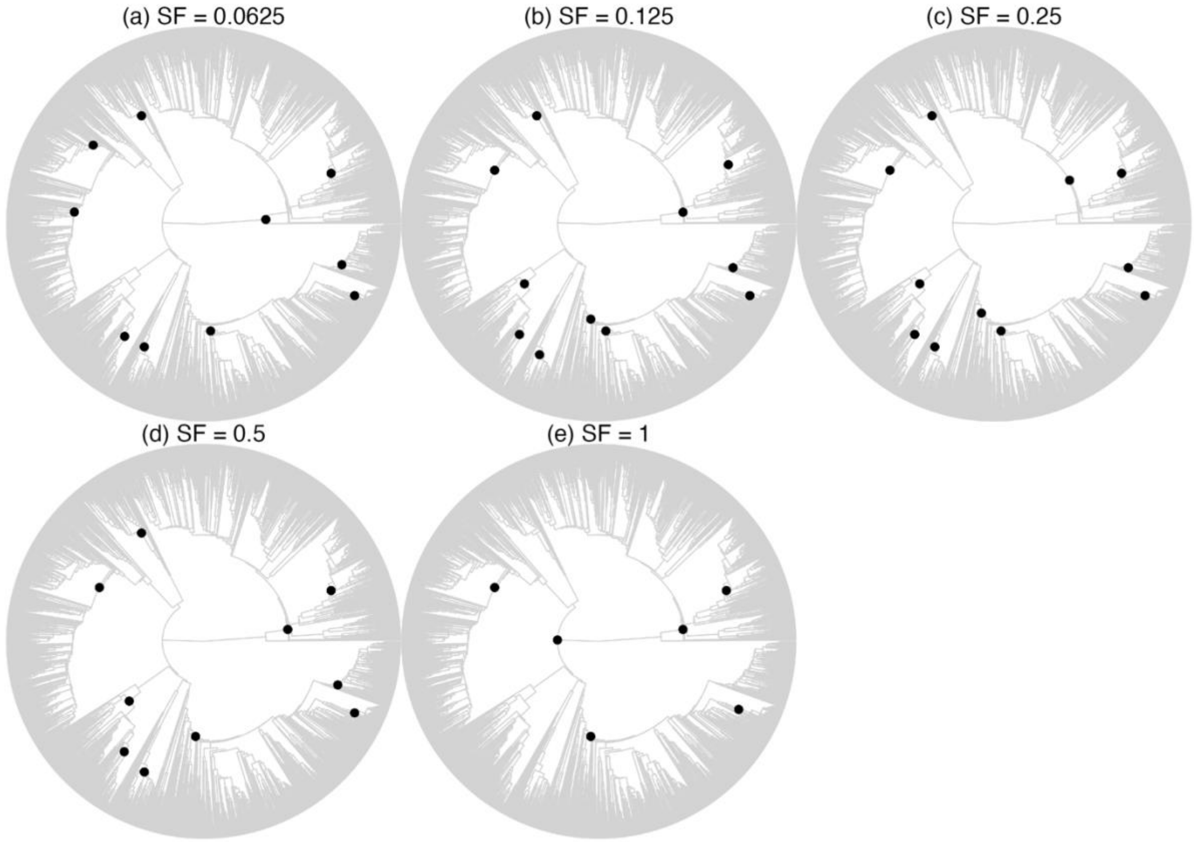

Figure S30. Position of nodes where core rate shifts have been inferred by BAMM in the 97.7% OTU *Myxococcota* phylogeny at different sampling fractions. The different panels show the core rate shifts identified by the shift configuration with the highest marginal probability at different sampling fractions (SF). There is some similarity between sampling fractions.

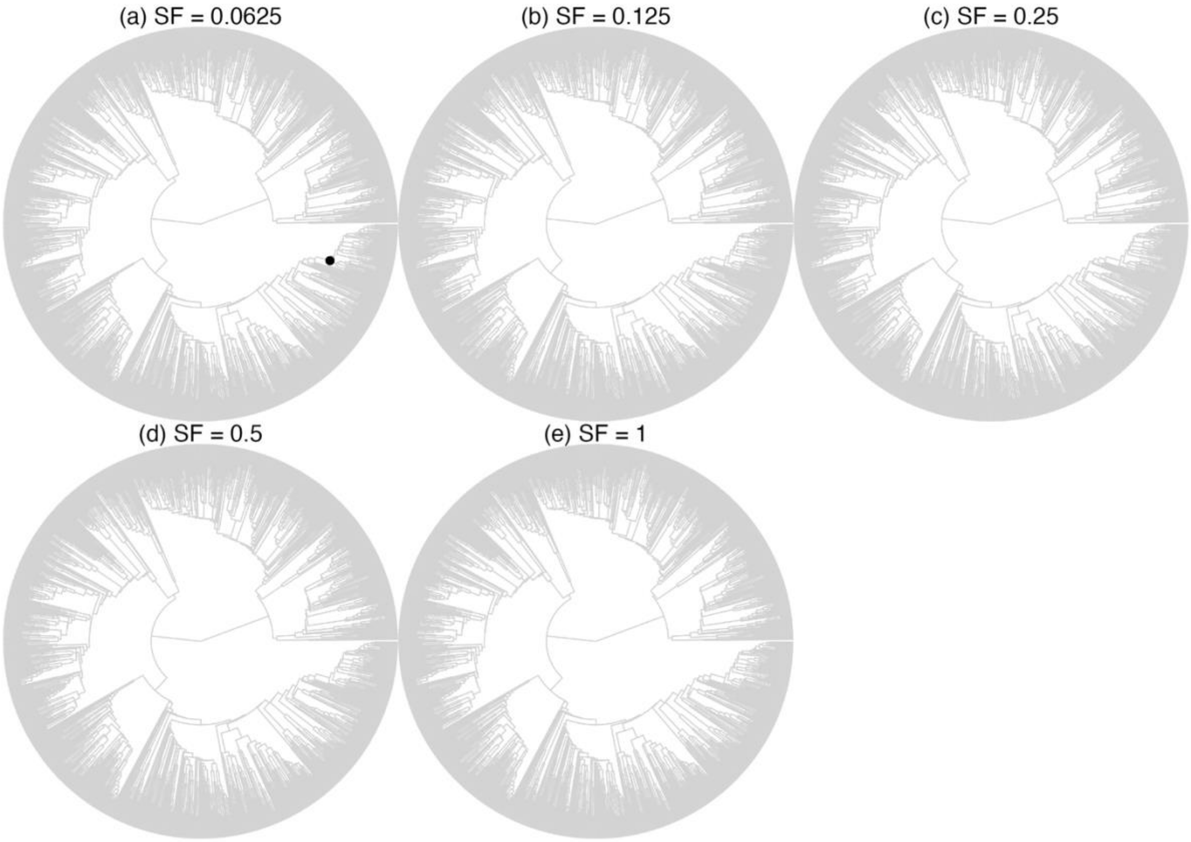

Figure S31. Position of nodes where core rate shifts have been inferred by BAMM in the 95% OTU *Myxococcota* phylogeny. The different panels show the core rate shifts identified by the shift configuration with the highest marginal probability at different sampling fractions (SF). The BAMM model does not find evidence of any rate shifts, indicating that rate shifts have happened closer to the present than deep in the past.

Table S4. Model comparison of multi-state and concealed trait diversification rate models at different assumed sampling fractions in the ASV tree.

Sampled fraction	Model	Number of estimated parameters	Log Likelihood	AIC	AIC weight
0.0625	CTD4	17	170.59	-307.19	1
0.0625	CTD3	10	148.40	-276.79	0
0.0625	MuHiSSE	13	104.45	-182.91	0
0.0625	CTD2	5	89.75	-169.50	0
0.0625	MuSSE	6	-560.92	1,133.84	0
0.125	CTD4	17	181.52	-329.05	1
0.125	CTD3	10	168.15	-316.31	0
0.125	MuHiSSE	13	124.96	-223.91	0
0.125	CTD2	5	114.66	-219.31	0
0.125	MuSSE	6	-423.29	858.58	0
0.25	CTD4	17	190.68	-347.36	1
0.25	CTD3	10	176.60	-333.19	0
0.25	MuHiSSE	13	138.83	-251.67	0
0.25	CTD2	5	123.73	-237.46	0
0.25	MuSSE	6	-355.95	723.90	0
1	CTD4	17	172.71	-311.42	1
1	CTD3	10	158.51	-297.03	0
1	MuHiSSE	13	119.96	-213.92	0
1	CTD2	5	99.37	-188.75	0
1	MuSSE	6	-237.20	486.39	0

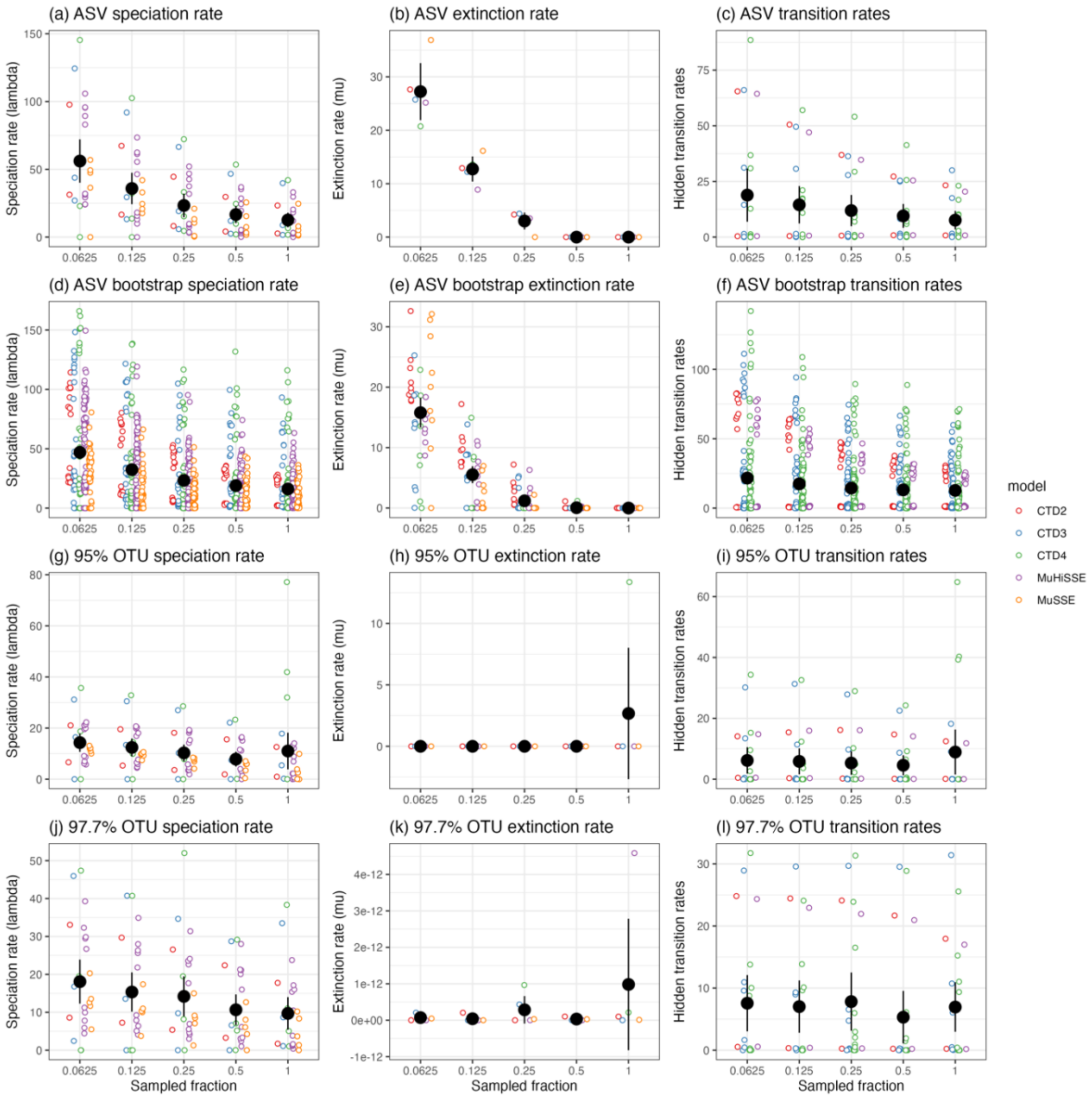

Figure S32. Model estimates of the multi-state and concealed trait diversification rate models for (a-c) the ASV tree (d-f) the ASV bootstrap replicates (g-i) the 95% OTU tree and (j-l) the 97.7% tree across different sampling fractions. Small points represent estimates from individual models, large points represent the mean across all models at that sampling fraction and lines represent confidence intervals across all models (2*standard error).

Table S5. Summary of the best model after model selection of multi-state and concealed trait diversification rate models at different estimated sampling fractions for 9 ASV bootstrap replicate trees.

Bootstrap	Sampled fraction	Model	Number of estimated parameters	Log Likelihood	AIC	AIC weight
ASV boot 1	0.0625	CTD4	17	-254.04	542.07	1.00
ASV boot 1	0.1250	CTD4	17	-235.98	505.96	1.00
ASV boot 1	0.2500	CTD4	17	-217.53	469.07	1.00
ASV boot 1	0.5000	CTD4	17	-211.96	457.92	1.00
ASV boot 1	1.0000	CTD4	17	-226.20	486.39	1.00
ASV boot 2	0.0625	CTD4	17	-386.57	807.15	1.00
ASV boot 2	0.1250	CTD4	17	-376.80	787.60	0.99
ASV boot 2	0.2500	CTD4	17	-361.92	757.85	1.00
ASV boot 2	0.5000	CTD4	17	-353.72	741.44	1.00
ASV boot 2	1.0000	CTD4	17	-363.37	760.74	1.00
ASV boot 3	0.0625	CTD4	17	151.32	-268.64	1.00
ASV boot 3	0.1250	CTD4	17	188.68	-343.37	1.00
ASV boot 3	0.2500	CTD4	17	211.07	-388.14	1.00
ASV boot 3	0.5000	CTD4	17	237.32	-440.63	1.00
ASV boot 3	1.0000	CTD4	17	237.94	-441.89	1.00
ASV boot 4	0.0625	CTD4	17	-130.51	295.02	1.00
ASV boot 4	0.1250	CTD4	17	-112.41	258.81	1.00
ASV boot 4	0.2500	CTD4	17	-98.92	231.83	1.00
ASV boot 4	0.5000	CTD4	17	-96.47	226.93	1.00
ASV boot 4	1.0000	CTD4	17	-105.81	245.61	1.00
ASV boot 5	0.0625	CTD4	17	98.49	-162.98	1.00
ASV boot 5	0.1250	CTD4	17	116.34	-198.67	1.00
ASV boot 5	0.2500	CTD4	17	138.60	-243.20	1.00
ASV boot 5	0.5000	CTD4	17	151.52	-269.04	1.00
ASV boot 5	1.0000	CTD4	17	146.27	-258.54	1.00
ASV boot 6	0.0625	CTD4	17	190.00	-346.01	1.00
ASV boot 6	0.1250	CTD4	17	216.95	-399.89	1.00
ASV boot 6	0.2500	CTD4	17	232.82	-431.65	1.00
ASV boot 6	0.5000	CTD4	17	234.71	-435.42	1.00
ASV boot 6	1.0000	CTD4	17	231.46	-428.92	1.00
ASV boot 7	0.0625	CTD4	17	-380.35	794.70	1.00
ASV boot 7	0.1250	CTD4	17	-362.82	759.64	1.00
ASV boot 7	0.2500	CTD4	17	-351.20	736.41	1.00
ASV boot 7	0.5000	CTD4	17	-351.95	737.89	1.00
ASV boot 7	1.0000	CTD4	17	-367.08	768.16	1.00
ASV boot 8	0.0625	CTD4	17	-181.12	396.23	1.00
ASV boot 8	0.1250	CTD4	17	-154.70	343.41	1.00
ASV boot 8	0.2500	CTD4	17	-125.21	284.43	1.00
ASV boot 8	0.5000	CTD4	17	-108.84	251.68	1.00
ASV boot 8	1.0000	CTD4	17	-110.88	255.77	1.00
ASV boot 9	0.0625	CTD4	17	350.70	-667.40	1.00
ASV boot 9	0.1250	CTD4	17	372.52	-711.04	1.00
ASV boot 9	0.2500	CTD4	17	385.09	-736.18	0.96
ASV boot 9	0.5000	CTD4	17	392.30	-750.60	0.77
ASV boot 9	1.0000	CTD4	17	391.65	-749.30	1.00

Table S6. Summary of the best model after model selection of multi-state and concealed trait diversification rate models at different estimated sampling fractions for the 95% OTU and 97.7% OTU trees.

OTU cut-off (%)	Sampled fraction	Model	Number of estimated parameters	Log Likelihood	AIC	AIC weight
95	0.0625	CTD4	17	-335.91	705.82	0.81
95	0.1250	CTD4	17	-356.66	747.32	0.99
95	0.2500	CTD4	17	-381.40	796.80	1.00
95	0.5000	CTD4	17	-407.16	848.32	1.00
95	1.0000	CTD4	17	-396.16	826.32	1.00
97.7	0.0625	CTD4	17	-264.79	563.58	1.00
97.7	0.1250	CTD4	17	-295.32	624.63	0.99
97.7	0.2500	CTD4	17	-332.80	699.59	1.00
97.7	0.5000	CTD4	17	-376.28	786.57	1.00
97.7	1.0000	CTD4	17	-419.09	872.17	1.00

Table S7. Model specification for the character dependent diversification rate (MuSSE) model. Numbers represent the five biome preferences (1 = freshwater + land generalist, 2 = freshwater specialist, 3 = marine generalist, 4 = marine specialist, 5 = land specialist). Letters represent hidden states (there are no hidden states in this model). The first table demonstrates speciation rate parameters, the second table extinction rate parameters, and the bottom table shows the parameters between states. Parameters that were fixed during the fitting process are in bold (all transitions between biome preferences).

Speciation rate:

1A	2A	3A	4A	5A
1	2	3	4	5

Extinction rate:

1A	2A	3A	4A	5A
6	6	6	6	6

Transition rates:

	1A	2A	3A	4A	5A
1A	-	**10**	**13**	-	**17**
2A	**7**	-	**14**	-	-
3A	**8**	**11**	-	**16**	-
4A	-	-	**15**	-	-
5A	**9**	**12**	-	-	-

Table S8. Model specification for the MuHiSSE model. Numbers represent the five biome preferences (1 = freshwater + land generalist, 2 = freshwater specialist, 3 = marine generalist, 4 = marine specialist, 5 = land specialist). Letters represent hidden states (there are 2 hidden states in this model). The first table demonstrates speciation rate parameters, the second table extinction rate parameters, and the bottom table shows the parameters between states. Parameters that were fixed during the fitting process are in bold (all transitions between biome preferences).

Speciation rate:

1A	2A	3A	4A	5A	1B	2B	3B	4B	5B
1	2	3	4	5	6	7	8	9	10

Extinction rate:

1A	2A	3A	4A	5A	1B	2B	3B	4B	5B
11	11	11	11	11	11	11	11	11	11

Transition rates:

	1A	2A	3A	4A	5A	1B	2B	3B	4B	5B
1A	-	**16**	**19**	-	**23**	24	-	-	-	-
2A	**12**	-	**20**	-	-	-	24	-	-	-
3A	**13**	**17**	-	**22**	-	-	-	24	-	-
4A	-	-	**21**	-	-	-	-	-	24	-
5A	**14**	**18**	-	-	-	-	-	-	-	24
1B	15	-	-	-	-	-	**16**	**19**	-	**23**
2B	-	15	-	-	-	**12**	-	**20**	-	-
3B	-	-	15	-	-	**13**	**17**	-	**22**	-
4B	-	-	-	15	-	-	-	**21**	-	-
5B	-	-	-	-	15	**14**	**18**	-	-	-

Table S9. Model specification for the character independent diversification (CTD2) rate model. Numbers represent the five biome preferences (1 = freshwater + land generalist, 2 = freshwater specialist, 3 = marine generalist, 4 = marine specialist, 5 = land specialist). Letters represent hidden states (there are 2 hidden states in this model). The first table demonstrates speciation rate parameters, the second table extinction rate parameters, and the bottom table shows the parameters between states. Parameters that were fixed during the fitting process are in bold (all transitions between biome preferences).

Speciation rate:

1A	2A	3A	4A	5A	1B	2B	3B	4B	5B
1	1	1	1	1	2	2	2	2	2

Extinction rate:

1A	2A	3A	4A	5A	1B	2B	3B	4B	5B
3	3	3	3	3	3	3	3	3	3

Transition rates:

	1A	2A	3A	4A	5A	1B	2B	3B	4B	5B
1A	-	**8**	**11**	-	**15**	16	-	-	-	-
2A	**4**	-	**12**	-	-	-	16	-	-	-
3A	**5**	**9**	-	**14**	-	-	-	16	-	-
4A	-	-	**13**	-	-	-	-	-	16	-
5A	**6**	**10**	-	-	-	-	-	-	-	16
1B	7	-	-	-	-	-	**8**	**11**	-	**15**
2B	-	7	-	-	-	**4**	-	**12**	-	-
3B	-	-	7	-	-	**5**	**9**	-	**14**	-
4B	-	-	-	7	-	-	-	**13**	-	-
5B	-	-	-	-	7	**6**	**10**	-	-	-

Table S10. Model specification for the character independent diversification rate (CTD3) model. Numbers represent the five biome preferences (1 = freshwater + land generalist, 2 = freshwater specialist, 3 = marine generalist, 4 = marine specialist, 5 = land specialist). Letters represent hidden states (there are 3 hidden states in this model). The first table demonstrates speciation rate parameters, the second table extinction rate parameters, and the bottom table shows the parameters between states. Parameters that were fixed during the fitting process are in bold (all transitions between biome preferences).

Speciation rate:

1A	2A	3A	4A	5A	1B	2B	3B	4B	5B	1C	2C	3C	4C	5C
1	1	1	1	1	2	2	2	2	2	3	3	3	3	3

Extinction rate:

1A	2A	3A	4A	5A	1B	2B	3B	4B	5B	1C	2C	3C	4C	5C
4	4	4	4	4	4	4	4	4	4	4	4	4	4	4

Transition rates:

	1A	2A	3A	4A	5A	1B	2B	3B	4B	5B	1C	2C	3C	4C	5C
1A	-	**10**	**13**	-	**17**	18	-	-	-	-	20	-	-	-	-
2A	**5**	-	**14**	-	-	-	18	-	-	-	-	20	-	-	-
3A	**6**	**11**	-	**16**	-	-	-	18	-	-	-	-	20	-	-
4A	-	-	**15**	-	-	-	-	-	18	-	-	-	-	20	-
5A	**7**	**12**	-	-	-	-	-	-	-	18	-	-	-	-	20
1B	8	-	-	-	-	-	**10**	**13**	-	**17**	21	-	-	-	-
2B	-	8	-	-	-	**5**	-	**14**	-	-	-	21	-	-	-
3B	-	-	8	-	-	**6**	**11**	-	**16**	-	-	-	21	-	-
4B	-	-	-	8	-	-	-	**15**	-	-	-	-	-	21	-
5B	-	-	-	-	8	**7**	**12**	-	-	-	-	-	-	-	21
1C	9	-	-	-	-	19	-	-	-	-	-	**10**	**13**	-	**17**
2C	-	9	-	-	-	-	19	-	-	-	**5**	-	**14**	-	-
3C	-	-	9	-	-	-	-	19	-	-	**6**	**11**	-	**16**	-
4C	-	-	-	9	-	-	-	-	19	-	-	-	**15**	-	-
5C	-	-	-	-	9	-	-	-	-	19	**7**	**12**	-	-	-

Table S11. Model specification for the character independent diversification rate (CTD4) model. Numbers represent the five biome preferences (1 = freshwater + land generalist, 2 = freshwater specialist, 3 = marine generalist, 4 = marine specialist, 5 = land specialist). Letters represent hidden states (there are 4 hidden states in this model). The first table demonstrates speciation rate parameters, the second table extinction rate parameters, and the bottom table shows the parameters between states. Parameters that were fixed during the fitting process are in bold (all transitions between biome preferences).

Speciation rate:

1A	2A	3A	4A	5A	1B	2B	3B	4B	5B	1C	2C	3C	4C	5C	1D	2D	3D	4D	5D
1	1	1	1	1	2	2	2	2	2	3	3	3	3	3	4	4	4	4	4

Extinction rate:

1A	2A	3A	4A	5A	1B	2B	3B	4B	5B	1C	2C	3C	4C	5C	1D	2D	3D	4D	5D
5	5	5	5	5	5	5	5	5	5	5	5	5	5	5	5	5	5	5	5

Transition rates:

	1A	2A	3A	4A	5A	1B	2B	3B	4B	5B	1C	2C	3C	4C	5C	1D	2D	3D	4D	5D
1A	-	**12**	**15**	-	**19**	20	-	-	-	-	23	-	-	-	-	26	-	-	-	-
2A	**6**	-	**16**	-	-	-	20	-	-	-	-	23	-	-	-	-	26	-	-	-
3A	**7**	**13**	-	**18**	-	-	-	20	-	-	-	-	23	-	-	-	-	26	-	-
4A	-	-	**17**	-	-	-	-	-	20	-	-	-	-	23	-	-	-	-	26	-
5A	**8**	**14**	-	-	-	-	-	-	-	20	-	-	-	-	23	-	-	-	-	26
1B	9	-	-	-	-	-	**12**	**15**	-	**19**	24	-	-	-	-	27	-	-	-	-
2B	-	9	-	-	-	**6**	-	**16**	-	-	-	24	-	-	-	-	27	-	-	-
3B	-	-	9	-	-	**7**	**13**	-	**18**	-	-	-	24	-	-	-	-	27	-	-
4B	-	-	-	9	-	-	-	**17**	-	-	-	-	-	24	-	-	-	-	27	-
5B	-	-	-	-	9	**8**	**14**	-	-	-	-	-	-	-	24	-	-	-	-	27
1C	10	-	-	-	-	21	-	-	-	-	-	**12**	**15**	-	**19**	28	-	-	-	-
2C	-	10	-	-	-	-	21	-	-	-	**6**	-	**16**	-	-	-	28	-	-	-
3C	-	-	10	-	-	-	-	21	-	-	**7**	**13**	-	**18**	-	-	-	28	-	-
4C	-	-	-	10	-	-	-	-	21	-	-	-	**17**	-	-	-	-	-	28	-
5C	-	-	-	-	10	-	-	-	-	21	**8**	**14**	-	-	-	-	-	-	-	28
1D	11	-	-	-	-	22	-	-	-	-	25	-	-	-	-	-	**12**	**15**	-	**19**
2D	-	11	-	-	-	-	22	-	-	-	-	25	-	-	-	**6**	-	**16**	-	-
3D	-	-	11	-	-	-	-	22	-	-	-	-	25	-	-	**7**	**13**	-	**18**	-
4D	-	-	-	11	-	-	-	-	22	-	-	-	-	25	-	-	-	**17**	-	-
5D	-	-	-	-	11	-	-	-	-	22	-	-	-	-	25	**8**	**14**	-	-	-
